# Late-life semaglutide treatment slows ageing and extends lifespan in female mice

**DOI:** 10.1038/s41586-026-10940-7

**Published:** 2026-09-02

**Authors:** Yufan Feng, Marine Barthez, Yifei Wang, Yibing Chen, Huixian Qiu, Chih-Ling Wang, Kartoosh Heydari, Melaine Delcroix, Lene Juel Rasmussen, Vilhelm A. Bohr, Danica Chen

**Affiliations:** 1 Department of Metabolic Biology and Nutrition, University of California, Berkeley, CA USA; 2 Cancer Research Laboratory, University of California, Berkeley, CA USA; 3 Department of Cellular and Molecular Medicine, University of Copenhagen, Copenhagen, Denmark; 4 Section on DNA Repair, National Institute on Aging, Baltimore, MD USA; 5 Center for Healthy Aging, University of Copenhagen, Copenhagen, Denmark

**Keywords:** Ageing, Preclinical research

## Abstract

Pharmacological glucagon-like peptide-1 receptor (GLP-1R) activation reduces food intake and is an effective therapy for type 2 diabetes and obesity^1^. The use of GLP-1 medicines has revealed pleiotropic beneficial effects beyond glucose and weight control^2–5^, but little is known about the underlying basis of the pleiotropic effects. Here, treatment of 20-month-old female C57BL/6 mice with the GLP-1R agonist semaglutide for 3 months improved physiological function, attenuated hallmarks of ageing and modulated nutrient sensors and conserved genetic regulators of ageing. Continued treatment extended mouse lifespan. These effects parallel key features of calorie restriction, a dietary intervention that slows ageing, extends lifespan and alleviates a wide spectrum of ageing-associated diseases^6^. In a longitudinal study in direct comparison to matched calorie restriction, semaglutide treatment preserved baseline function and recapitulated many functional benefits of calorie restriction by attenuating age-associated decline, while also producing improvements above baseline and more favourable trajectories than calorie restriction in exploratory drive, spatial memory and glucose control. Together, these findings demonstrate that GLP-1R activation initiated late in life slows ageing and extends lifespan in female mice, supporting its function as a calorie restriction mimetic and providing a mechanistic framework that may help to explain its broad beneficial effects while revealing effects beyond those attributable to reduced calorie intake.

## Abstract (web summary)

Semaglutide treatment for 3 months in female mice improved physiological function, attenuated hallmarks of ageing and modulated nutrient sensors and conserved genetic regulators of ageing.

## Main

Calorie restriction, typically defined as reduced calorie intake without malnutrition, slows ageing and extends lifespan across species^6^. This dietary regimen also improves physiology and ameliorates a wide spectrum of seemingly unrelated diseases, such as metabolic diseases, neurodegenerative diseases, cancer and immune disorders^6^. Calorie restriction engages several evolutionarily conserved signalling pathways, such as sirtuins and insulin–IGF1 signalling, to elicit a beneficial response^7–9^. However, it is difficult to practice sustained calorie restriction. There is a need to identify calorie restriction mimetics that capture the health and longevity benefits and are suitable for human applications.

GLP-1 is secreted from gut endocrine cells in response to food ingestion, potentiates glucose-dependent insulin secretion and inhibits food intake^10^. Pharmacological GLP-1 receptor (GLP-1R) activation is an effective therapy for type 2 diabetes and obesity^1^. The use of GLP-1 medicines has revealed pleiotropic beneficial effects beyond glucose and weight control, such as reduction of heart, kidney and liver diseases, and neurodegeneration^2–5^. Little is known about the underlying basis of the pleiotropic effects. As GLP-1 administration inhibits food intake through brain GLP-1R activation^11^, we hypothesized that GLP-1 medicines might function as a mimetic of calorie restriction that slows ageing and alleviates various ageing-associated diseases. The effects of calorie restriction are influenced by individual characteristics, including factors such as age and health condition^12–14^. These considerations raise the question of how long-term use of GLP-1 medicines influences the ageing process and lifespan, particularly when treatment is initiated late in life.

## Lifespan

**Fig. 1:**
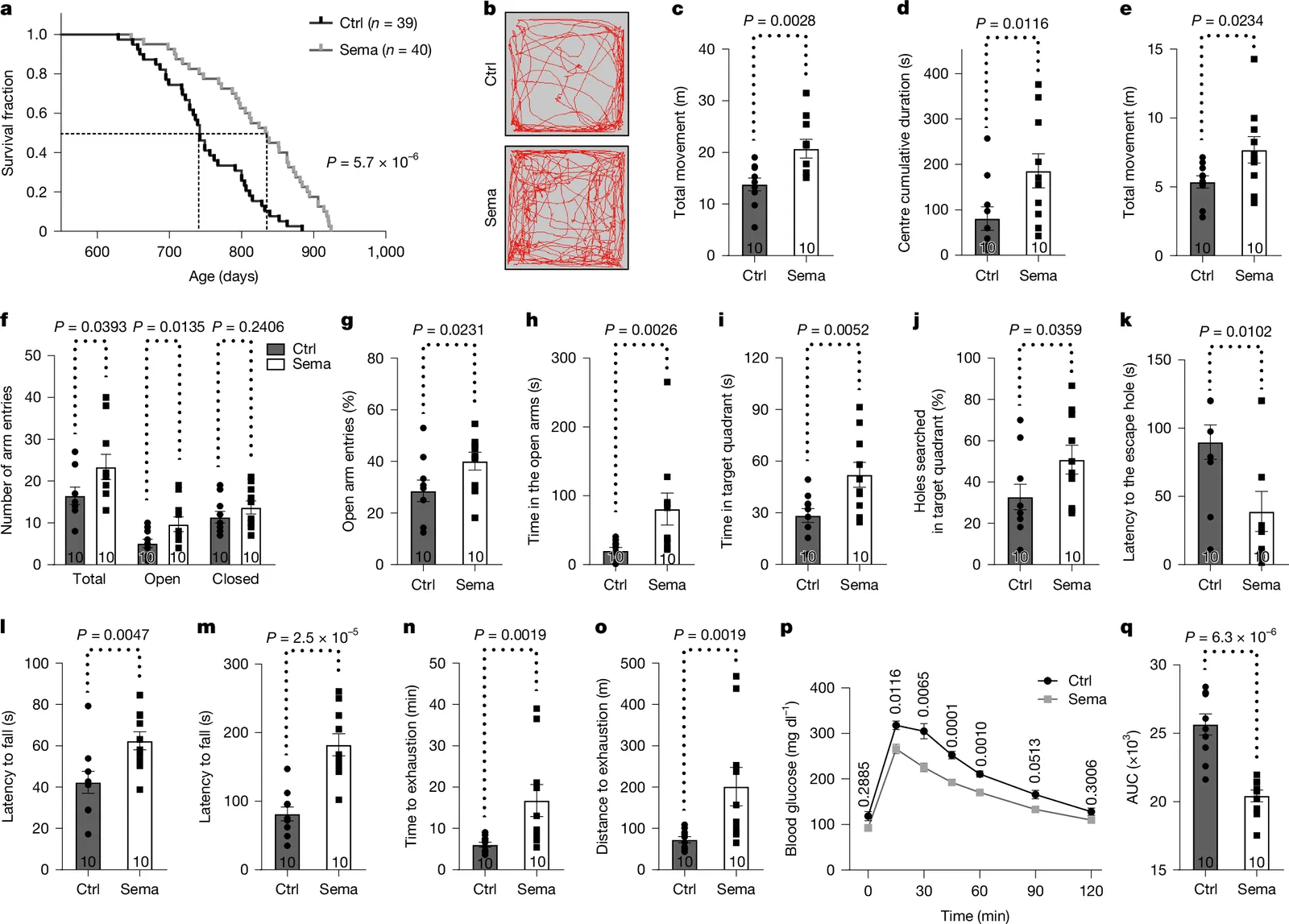
GLP-1R activation late in life extends lifespan and improves physiological function. **a**, 20-month-old female C57BL/6 mice were treated with vehicle (control, Ctrl) or semaglutide (sema) for the duration of the lifespan. Kaplan–Meier survival curves. The dashed lines indicate the median lifespan. **b**–**q**, Comparison of 20-month-old female C57BL/6 mice treated with vehicle or semaglutide for 3 months. **b**–**d**, Representative traces (**b**), total movement (**c**) and centre cumulative duration (**d**) in the open-field test. **e**–**h**, Total movement (**e**), the number of arm entries (**f**), the percentage of open arm entries (**g**) and time in the open arms (**h**) in the elevated plus maze test. **i**–**k**, Time in target quadrant (**i**), the percentage of holes searched in target quadrant (**j**) and the latency to the escape hole (**k**) in the Barnes maze test. **l**, The latency to fall in the rotarod test. **m**, The latency to fall in the inverted screen test. **n**,**o**, Time (**n**) and distance to exhaustion (**o**) in the treadmill exhaustion test. **p**,**q**, Blood glucose (**p**) and area under the curve (AUC) (**q**) in the glucose-tolerance test. Data are mean ± s.e.m.* P* values are shown in the figures and are provided as source data. The statistical tests used are described in the source data. Sample sizes (the numbers of mice) are shown in the figures. Source data

**Extended Data Fig. 1:**
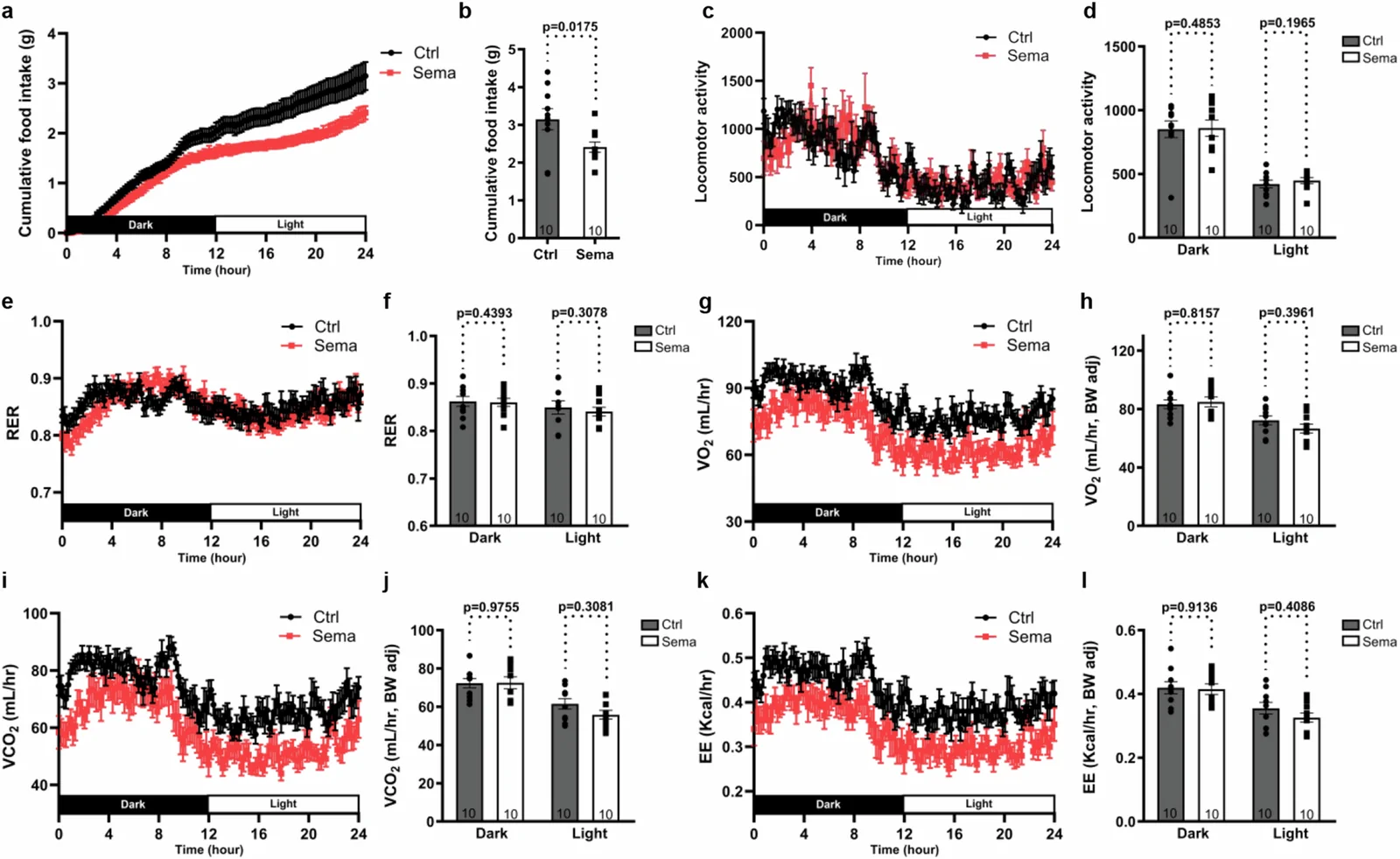
Effects of GLP-1R activation late in life on metabolic cage measures. Comparison of 20-month-old female C57BL/6 mice treated with vehicle or semaglutide for 3 months in metabolic cage. a, b, Cumulative food intake (a) and summarized (b). c, d, Locomotor activity (c) and summarized by light and dark cycles (d). e, f, Respiratory exchange ratio (e) and summarized by light and dark cycles (f). g, h, Oxygen consumption absolute value (g) and summarized by light and dark cycles after adjustment using ANCOVA with body weight as a covariate (h). i, j, Carbon dioxide production absolute value (i) and summarized by light and dark cycles after adjustment using ANCOVA with body weight as a covariate (j). k, l, Energy expenditure absolute value (k) and summarized by light and dark cycles after adjustment using ANCOVA with body weight as a covariate (l). Data are shown as mean ± SEM. P values are included in figures and source data. Statistical tests are included in source data. Sample sizes (the numbers of mice) are included in figures. Source data

**Extended Data Fig. 2:**
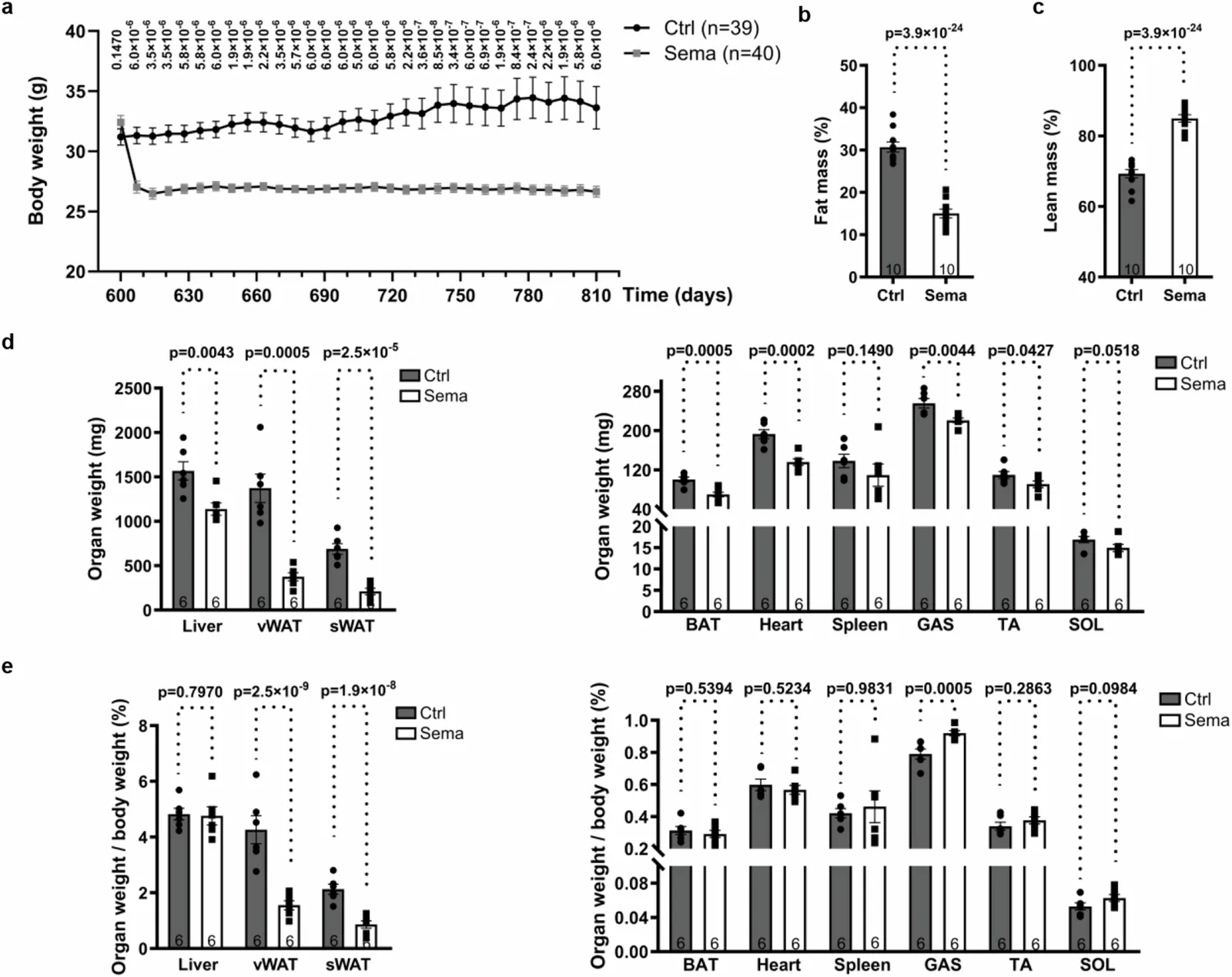
GLP-1R activation late in life reduces body weight and body fat. 20-month-old female C57BL/6 mice were treated with vehicle or semaglutide. a, Body weight of mice in the lifespan study. b, c, The percentage of fat mass (b) and lean mass (c). d, e, Organ weights (d) and indexed organ weights (e). Data are shown as mean ± SEM. P values are included in figures and source data. Statistical tests are included in source data. Sample sizes (the numbers of mice) are included in figures. Source data

**Extended Data Fig. 3:**
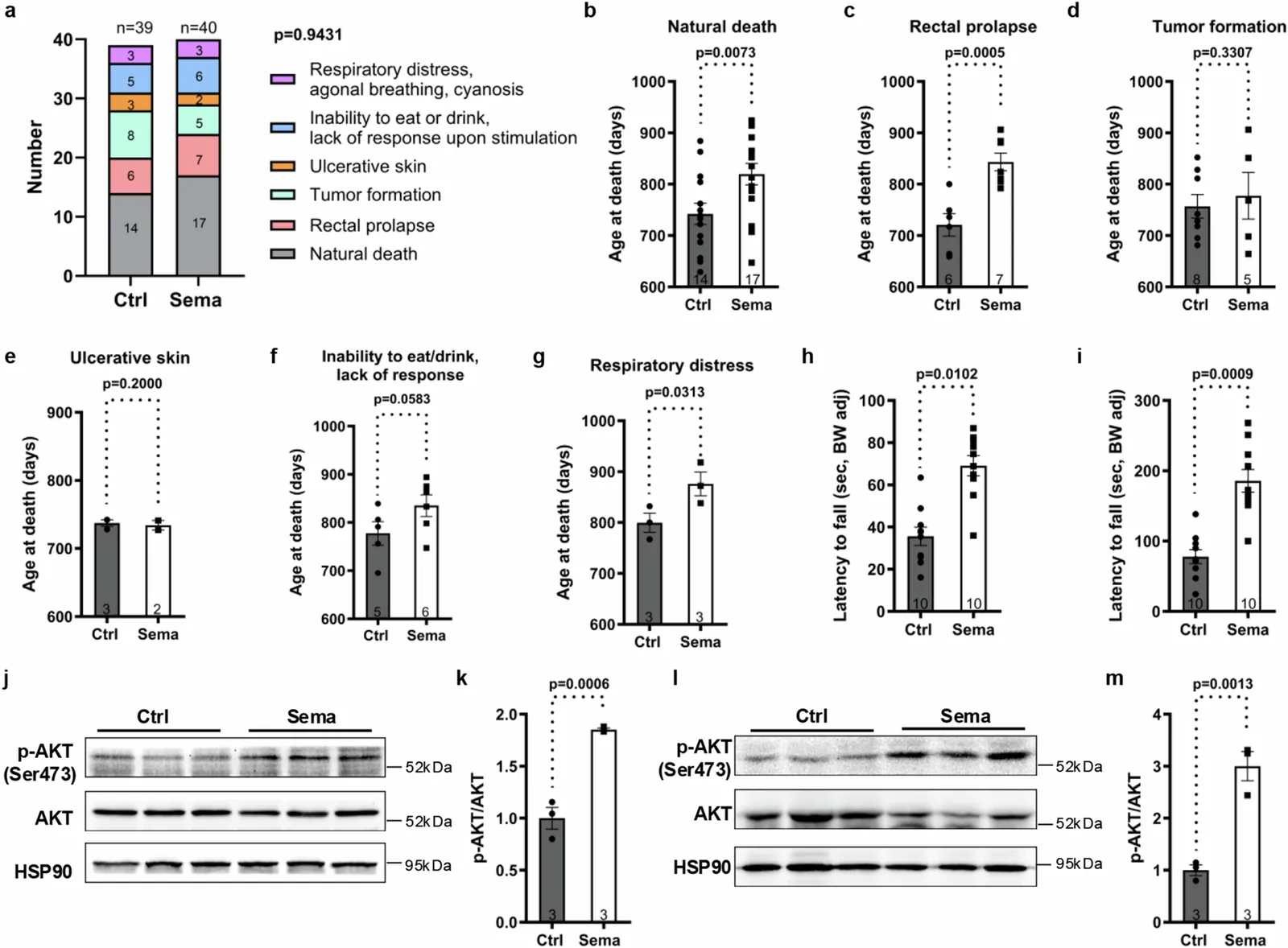
GLP-1R activation late in life extends lifespan and improves physiological function. 20-month-old female C57BL/6 mice were treated with vehicle or semaglutide. a, Distribution of endpoint categories in the lifespan study. b-g, Age at death for the indicated endpoint categories in the lifespan study. h, Latency to fall in the rotarod test after adjustment using ANCOVA with body weight as a covariate. i, Latency to fall in the inverted screen test after adjustment using ANCOVA with body weight as a covariate. j-m, Western blot analyses and quantification for phosphorylated Akt in the liver (j, k) and the vWAT (l, m). Data are shown as mean ± SEM. P values are included in figures and source data. Statistical tests are included in source data. Sample sizes (the numbers of mice) are included in figures. Source data

We treated 20-month-old female C57BL/6 mice daily with either vehicle or semaglutide, a GLP-1R agonist, through subcutaneous injection. For lifespan study, treatment continued for the duration of the lifespan. For physiological, molecular and cellular studies, a separate cohort was treated for 3 months. Semaglutide treatment reduced food intake by 24% (Extended Data Fig. 1a,b), but did not significantly alter locomotor activity (Extended Data Fig. 1c,d), respiratory exchange ratio (Extended Data Fig. 1e,f; an indicator of fuel utilization), oxygen consumption (Extended Data Fig. 1g,h), carbon dioxide production (Extended Data Fig. 1i,j) and energy expenditure (Extended Data Fig. 1k,l) after adjustment using analysis of covariance (ANCOVA) with body weight as a covariate. As described previously^15–17^, mice in the control group maintained body weight after 20 months of age (Extended Data Fig. 2a). There was no difference in body weight between groups before treatment (Extended Data Fig. 2a), and semaglutide treatment reduced body weight and tissue weight (Extended Data Fig. 2a–e) as reported previously^1^. The weight loss predominantly came from body fat loss, as semaglutide treatment did not reduce the percentage of weight for most tissues except for the white adipose tissues, resulting in a reduced percentage of fat mass and increased percentage of lean mass (Extended Data Fig. 2b–e). The lifespan of the control group was within the reported range for C57BL/6 mice^13,18–22^ (Fig. 1a). Notably, semaglutide-treated mice showed significantly longer lifespans (median lifespan, 742 days (control) and 834 days (semaglutide); Fig. 1a). The distribution of end-point categories was consistent with the literature^16^, and did not differ significantly between groups (Extended Data Fig. 3a). Age at death appeared to be delayed by semaglutide across several categories unrelated to tumour (Extended Data Fig. 3b–g), consistent with a broad delay in age-associated physiological deterioration.

## Physiological function

Ageing leads to physiological decline, including reduced locomotor activity and exploratory behaviour in a novel environment^23^, decreased motor coordination^22^, poor muscle function^24^, metabolic dysregulation^25^ and cognitive decline^26^. Semaglutide-treated aged mice showed increased locomotor and exploratory activities in a novel environment, as demonstrated by increased total movement in the open-field test and the elevated plus maze test, increased centre cumulative duration in the open-field test and increased entries to the open arms in the elevated plus maze test (Fig. 1b–h). In the Barnes maze test, semaglutide-treated mice showed increased time in the target quadrant, increased holes searched in the target quadrant and reduced latency to the escape hole, indicative of improved cognition (Fig. 1i–k). Semaglutide-treated mice showed improved motor function based on the rotarod test (Fig. 1l) and improved muscle function, as evidenced by increased latency to fall in the inverted screen test (Fig. 1m) and increased time and distance to exhaustion in the treadmill exhaustion test (Fig. 1n,o). To determine whether improvements in rotarod and inverted screen performance were independent of body weight, we performed ANCOVA with body weight as a covariate. After adjustment for body weight, the semaglutide group retained a significant increase in latency to fall compared with the controls (Extended Data Fig. 3h,i). Semaglutide treatment also improved metabolic homeostasis in aged mice, as semaglutide-treated mice cleared glucose more effectively in the glucose-tolerance test (Fig. 1p,q). Insulin sensitivity was improved, as evidenced by increased phosphorylation of protein kinase B (PKB, also known as AKT) in metabolic tissues (Extended Data Fig. 3j–m). Together, these data suggest that GLP-1R activation late in life improves physiological function.

## Hallmarks of ageing

**Fig. 2:**
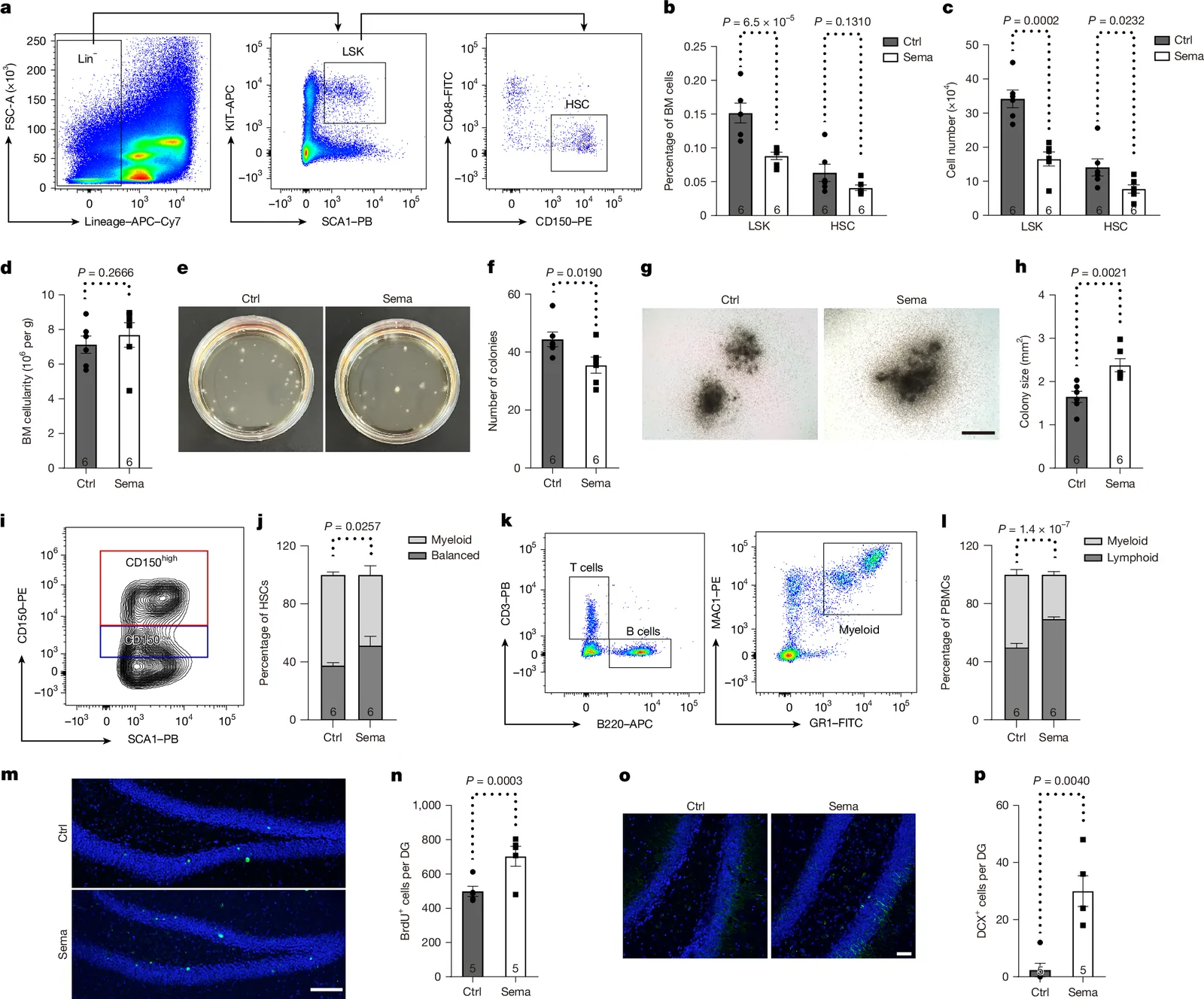
GLP-1R activation late in life ameliorates stem cell ageing. Comparison of 20-month-old female C57BL/6 mice treated with vehicle or semaglutide for 3 months. **a**, The gating strategy of HSCs in the bone marrow. LSK, Lin^−^SCA1^+^KIT^+^ cells; HSC, Lin^−^SCA1^+^KIT^+^CD48^−^CD150^+^ cells. APC, allophycocyanin; Cy7, cyanine 7; FITC, fluorescein isothiocyanate; PB, pacific blue; PE, phycoerythrin. **b**,**c**, The frequency (**b**) and number (**c**) of HSCs in the bone marrow (BM). **d**, Bone marrow cellularity normalized to body weight. **e**,**f**, Representative images (**e**) and quantification of the number of colonies (**f**) for the colony-forming unit assay. **g**,**h**, Representative images for colonies (**g**) and quantification of colony size (**h**) for the colony-forming unit assay. Scale bar, 1 mm. **i**,**j**, Gating strategy (**i**) and quantification (**j**) of myeloid-biased HSCs (Lin^−^KIT^+^SCA1^+^CD135^−^CD34^−^CD150^high^) and lineage-balanced HSCs (Lin^−^KIT^+^SCA1^+^CD135^−^CD34^−^CD150^low^) in the bone marrow. **k**,**l**, Gating strategy (**k**) and quantification (**l**) of lineage differentiation (B cells, B220^+^; T cells, CD3^+^; myeloid cells, GR1^+^MAC1^+^) in the peripheral blood mononuclear cells (PBMCs). **m**,**n**, Immunostaining (**m**) and quantification (**n**) of BrdU^+^ cells in the dentate gyrus (DG). Scale bar, 100 µm. **o**,**p**, Immunostaining (**o**) and quantification (**p**) of DCX^+^ cells in the dentate gyrus. Scale bar, 50 µm. Data are mean ± s.e.m. *P* values are included in figures and source data. Statistical tests are included in source data. Sample sizes (the numbers of mice) are shown in the figures. Source data

Ageing is associated with well-characterized hallmarks of ageing, such as stem cell attrition, inflammation, cellular senescence, mitochondrial dysfunction, loss of proteostasis and genomic instability^27^. We examined the effects of semaglutide treatment on the established hallmarks of ageing. Maintenance of the haematopoietic system throughout the adult life relies on the persistence of haematopoietic stem cells (HSCs). HSCs are capable of self-renewing to give rise to daughter stem cells and of differentiating to give rise to all the blood cell types including the lymphoid and myeloid lineages. During ageing, the number of phenotypically defined HSCs is increased, while aged HSCs have diminished regenerative capacity and differentiation potential biased toward the myeloid lineage^28–31^. Semaglutide-treated aged mice showed reduced phenotypically defined enriched (Lin^−^SCA1^+^KIT^+^, LSK cells) and highly enriched (Lin^−^SCA1^+^KIT^+^CD150^+^CD48^−^) HSCs in the bone marrow (Fig. 2a–c). Despite a reduced number of HSCs, semaglutide-treated mice had comparable bone marrow cellularity (Fig. 2d), suggesting an increased regenerative capacity per cell. Consistent with reduced number of HSCs and increased regenerative capacity in semaglutide-treated mice, the number of colony-forming cells in the bone marrow was reduced (Fig. 2e,f), while the colony size was bigger (Fig. 2g,h). Semaglutide treatment reduced the percentage of myeloid-biased HSCs in the bone marrow (Fig. 2i,j) and reduced myeloid-biased differentiation in the peripheral blood (Fig. 2k,l). Thus, GLP-1R activation late in life alleviates HSC ageing.

The dentate gyrus of the hippocampus is a brain region involved in learning, memory formation and spatial coding. Neural stem cells (NSCs) in the subgranular zone of the dentate gyrus generate new granule cells throughout life and this neurogenesis process contributes to learning and memory formation^32^. Ageing results in a decline in NSCs, neurogenesis and cognitive function^26,33^. Adult hippocampal neurogenesis is disrupted in patients affected by several neurodegenerative disorders^34^. Semaglutide treatment increased the numbers of 5-bromo-2′-deoxyuridine-positive (BrdU^+^) cells (Fig. 2m,n), and newly differentiated doublecortin-positive (DCX^+^) neurons (Fig. 2o,p) in the dentate gyrus, suggesting that GLP-1R activation late in life ameliorates NSC ageing and promotes neurogenesis in the dentate gyrus, contributing to enhanced cognition and amelioration of neurodegeneration (Fig. 1i–k).

**Extended Data Fig. 4:**
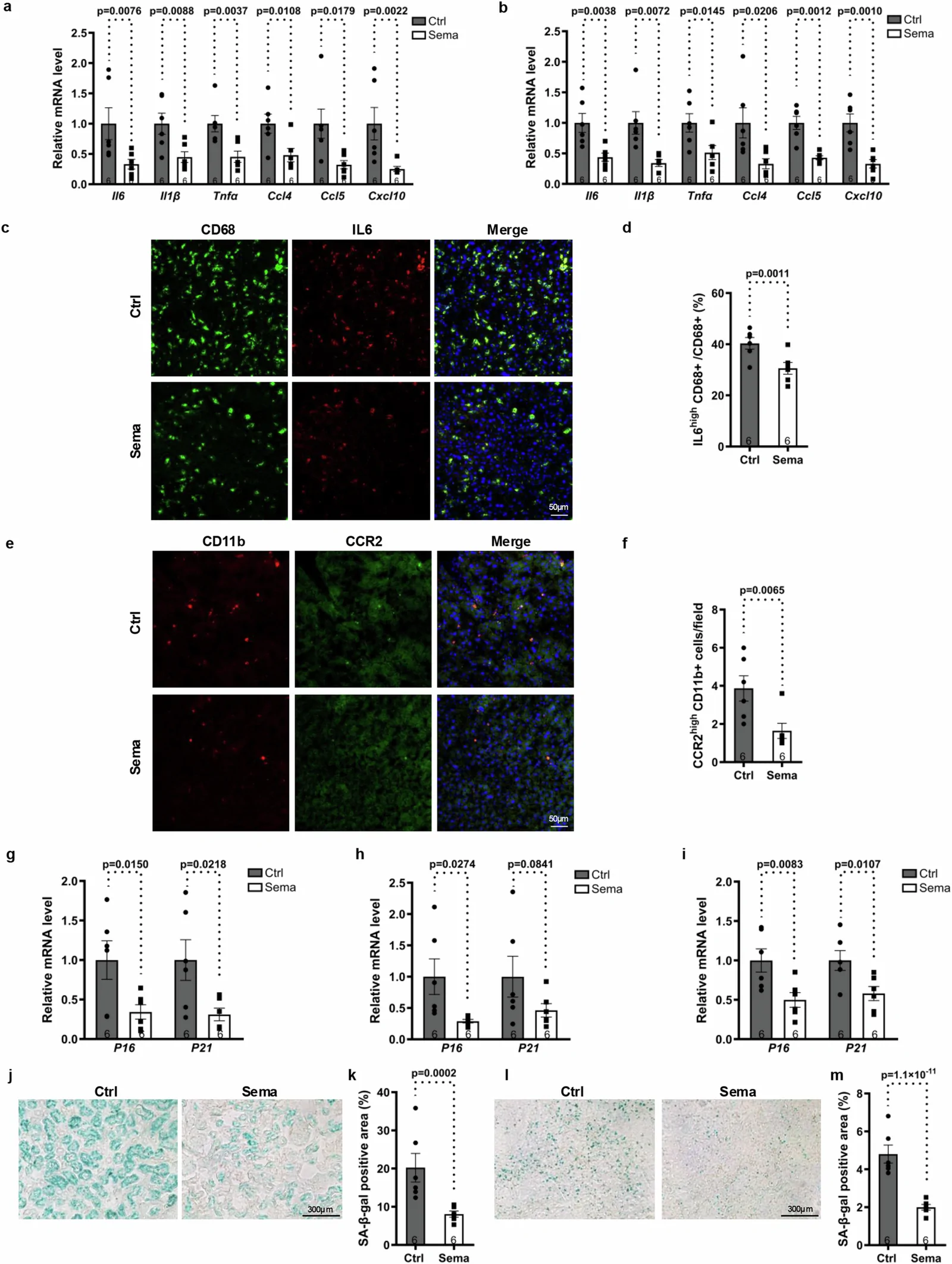
GLP-1R activation late in life reduces aging-associated inflammation and cellular senescence. Comparison of 20-month-old female C57BL/6 mice treated with vehicle or semaglutide for 3 months. a, b, Quantitative real-time PCR analyses of pro-inflammatory cytokines in the liver (a) and the muscle (b). c, d, Representative images (c) and quantification of immunostaining for CD68^+^ cells that express high levels of IL-6 (d) of liver sections. Blue: DAPI. Green: CD68. Red: IL-6. Scale bar: 50 μm. e, f, Representative images (e) and quantification of immunostaining for CCR2^high^CD11b^+^ cells (f) of liver sections. Blue: DAPI. Green: CCR2. Red: CD11b. Scale bar: 50 μm. g-i, Quantitative real-time PCR analyses of p16 and p21 in the liver (g), the vWAT (h), and the spleen (i). j-m, Representative images of SA-β-gal staining and quantification in the kidney (j, k) and the spleen (l, m) sections. Scale bar: 300 μm. Data are shown as mean ± SEM. P values are included in figures and source data. Statistical tests are included in source data. Sample sizes (the numbers of mice) are included in figures. Source data

**Extended Data Fig. 5:**
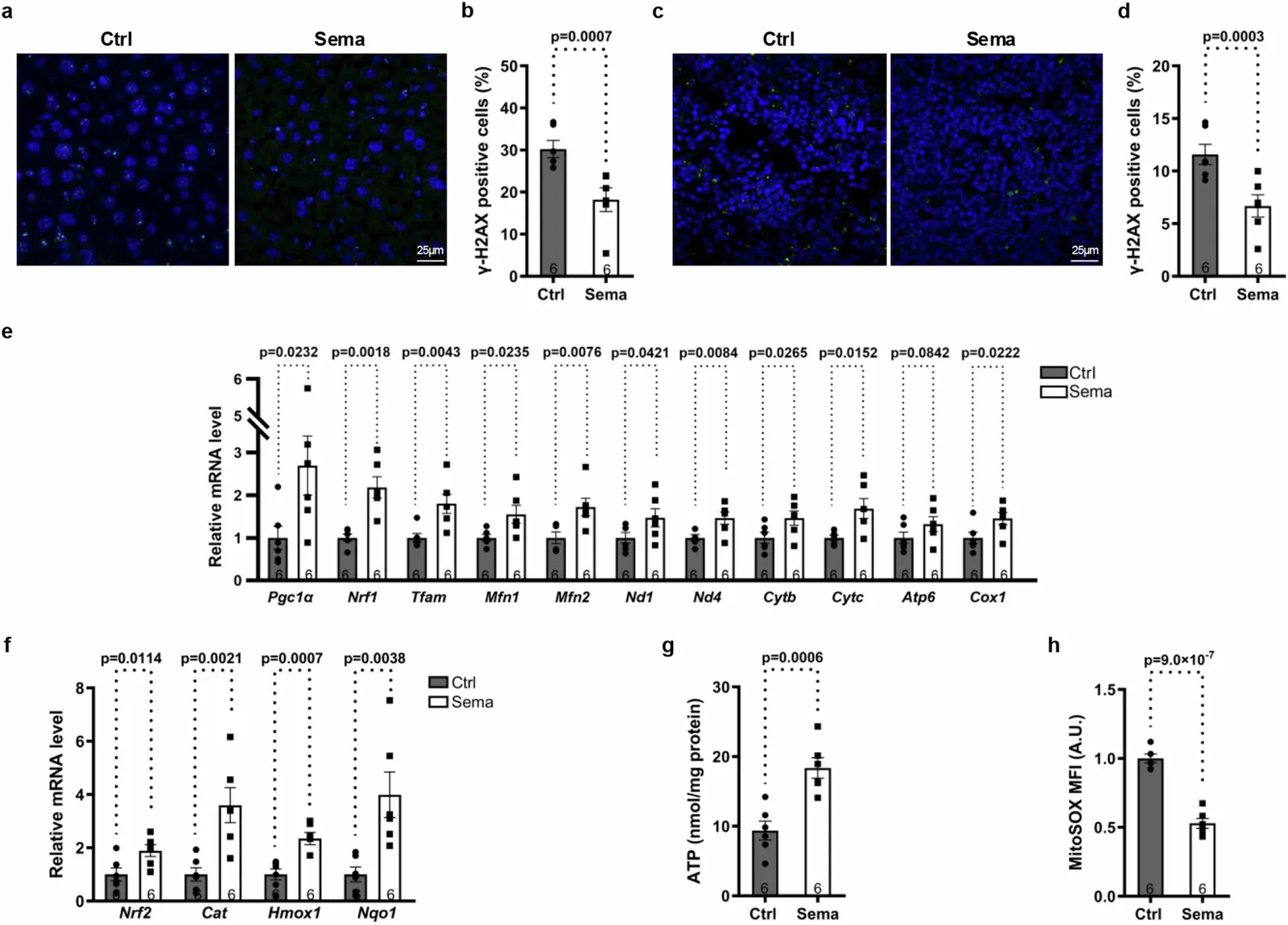
GLP-1R activation late in life improves genomic stability, mitochondrial function, and the oxidative stress response. Comparison of 20-month-old female C57BL/6 mice treated with vehicle or semaglutide for 3 months. a-d, Representative immunostaining images and quantification of γ-H2AX^+^ cells in the liver (a, b) and the spleen (c, d) sections. Blue: DAPI. Green: γ-H2AX. Scale bar: 25 μm. e, f, Quantitative real-time PCR analyses of mitochondrial genes in the muscle (e) and the oxidative stress response genes in the vWAT (f). g, Quantification of ATP in the muscle. h, Quantification of flow cytometry analysis of MitoSOX staining of HSCs. Data are shown as mean ± SEM. P values are included in figures and source data. Statistical tests are included in source data. Sample sizes (the numbers of mice) are included in figures. Source data

Semaglutide treatment reduced ageing-associated inflammation, manifested by increased expression levels of inflammatory cytokines and tissue infiltration of macrophages^35^. In semaglutide-treated mice, the expression of inflammatory cytokines was reduced (Extended Data Fig. 4a,b). In the tissues of mice treated with semaglutide, the percentage of CD68^+^ cells expressing high levels of IL-6 was reduced (Extended Data Fig. 4c,d) and the number of Ly6C^high^ monocytes that differentiate into pro-inflammatory macrophages (CD11b^+^CCR2^high^) was also reduced^36^ (Extended Data Fig. 4e,f). Semaglutide treatment also reduced cellular senescence, based on the well-established markers, such as the expression of p16 and p21 (Extended Data Fig. 4g–i) and senescence-associated β-galactosidase (SA-β-gal) staining^37^ (Extended Data Fig. 4j–m). Genomic stability was improved by semaglutide treatment, as the number of cells positive for the phosphorylated form of histone variant H2AX (γ-H2AX) was reduced^38^ (Extended Data Fig. 5a–d). Semaglutide also increased the expression of mitochondria-related genes (Extended Data Fig. 5e) and the oxidative-stress-response genes (Extended Data Fig. 5f), increased ATP content (Extended Data Fig. 5g) and reduced the levels of reactive oxygen species (Extended Data Fig. 5h), indicating improved mitochondrial function and oxidative stress response.

**Extended Data Fig. 6:**
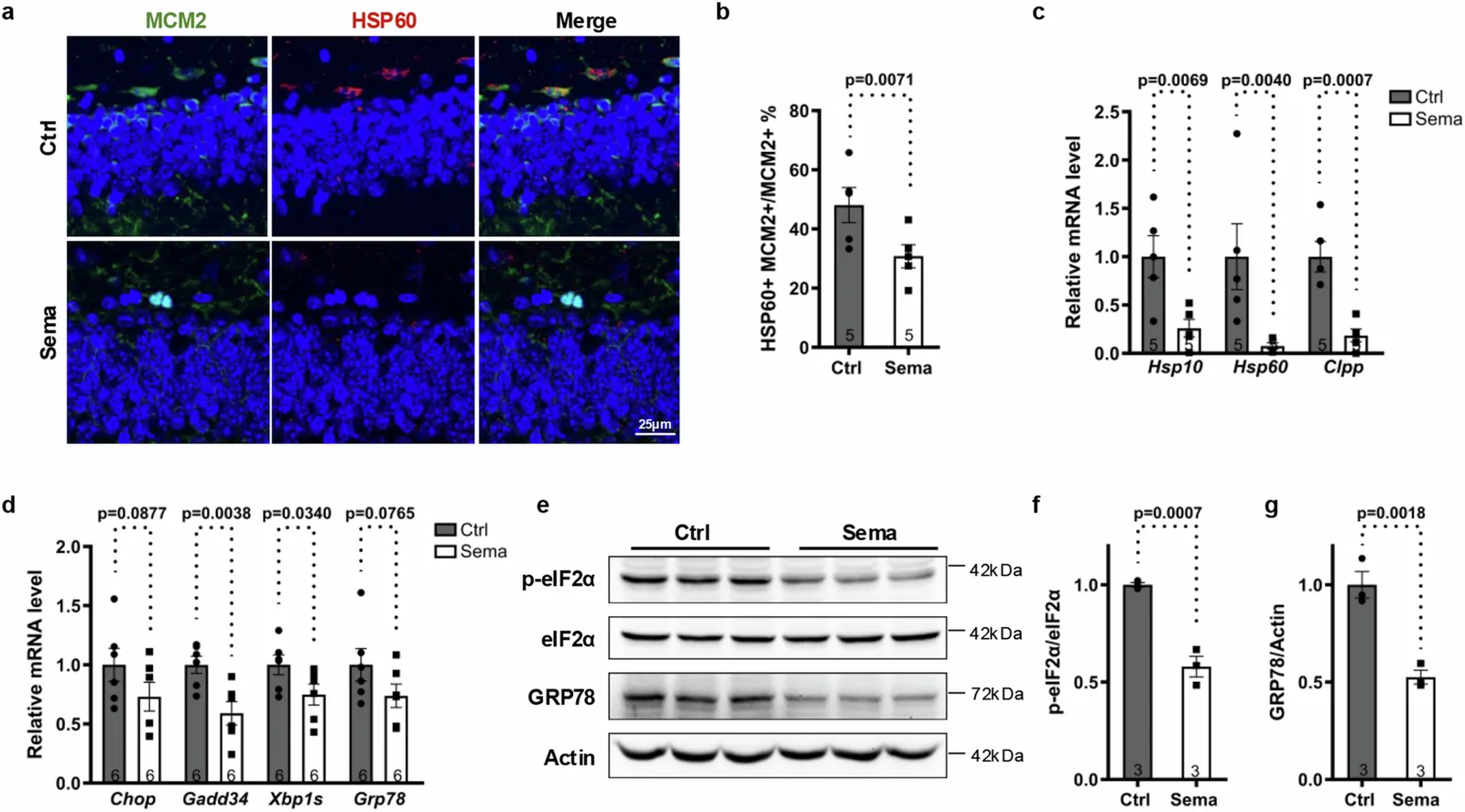
GLP-1R activation late in life improves proteostasis. Comparison of 20-month-old female C57BL/6 mice treated with vehicle or semaglutide for 3 months. a, b, Representative images (a) and quantification (b) of immunostaining for MCM2+ cells that express HSP60 in the dentate gyrus. Blue: DAPI. Red: HSP60. Green: MCM2. Scale bar: 25 µm. c, Quantitative real-time PCR analyses of the UPR^mt^ genes in HSCs. d, Quantitative real-time PCR analyses of the UPR^ER^ genes in the liver. e-g, Western blot analyses (e) and quantification for phosphorylated eIF2α (f) and GRP78 (g) in the liver. Data are shown as mean ± SEM. P values are included in figures and source data. Statistical tests are included in source data. Sample sizes (the numbers of mice) are included in figures. Source data

Cellular protein homeostasis, or proteostasis, is maintained by an array of protein quality-control mechanisms. The unfolded protein response (UPR) is activated in response to the accumulation of unfolded proteins in the mitochondria (UPR^mt^) or the endoplasmic reticulum (UPR^ER^), resulting in suppression of protein translation and increased production of chaperones and proteases to help cells return to proteostasis. The mitochondrial protein folding stress is increased in HSCs and NSCs during ageing^29,33^, and was suppressed by semaglutide treatment, as evidenced by the expression of mitochondrial chaperones and proteases^29,33^ (Extended Data Fig. 6a–c). Endoplasmic reticulum stress is increased in the liver during aging^39^, and was suppressed by semaglutide treatment, as indicated by the expression of the endoplasmic reticulum-stress-responsive genes and phosphorylation of eIF2α, which controls translation in response to stress^40,41^ (Extended Data Fig. 6d–g).

**Fig. 3:**
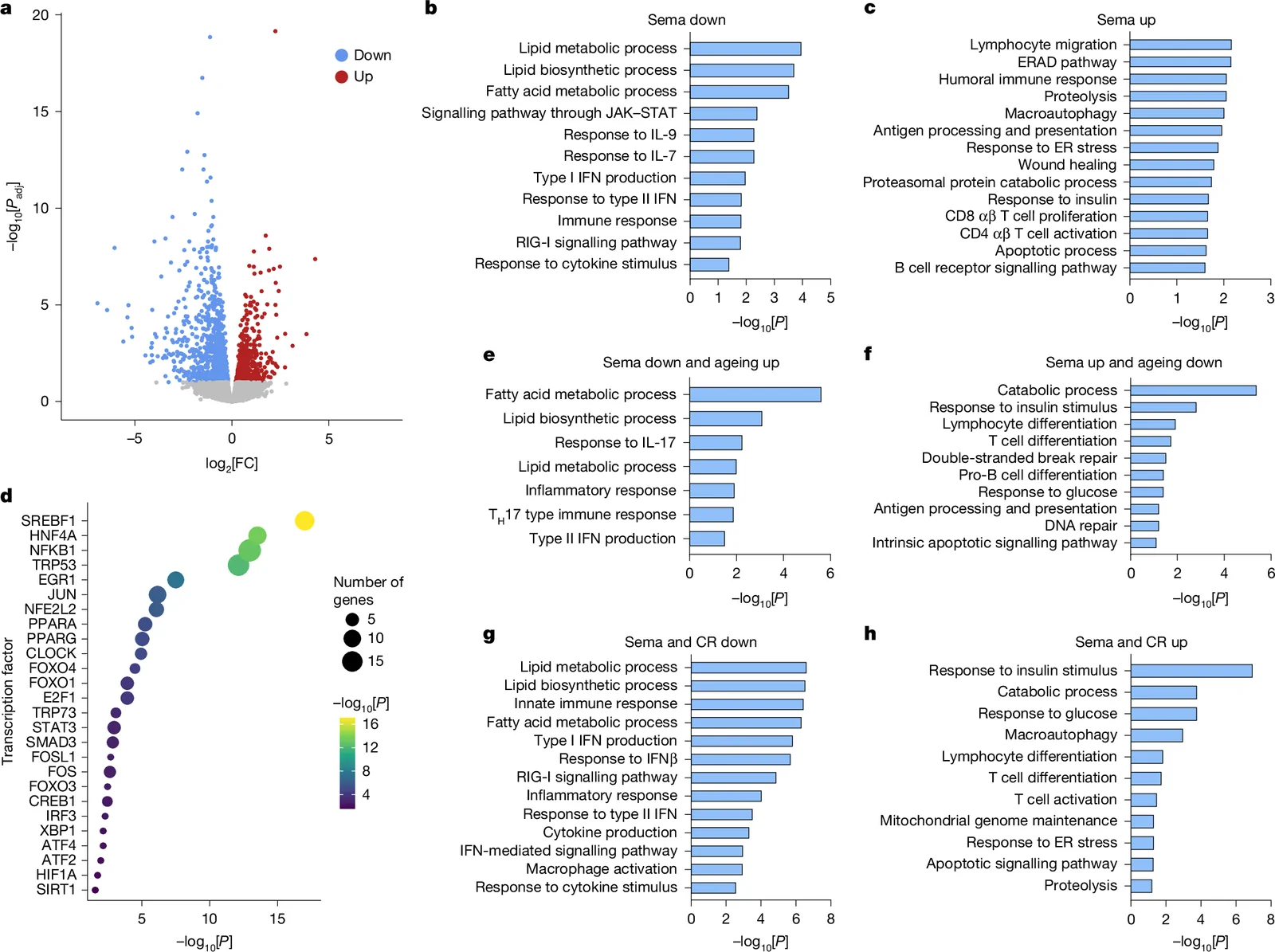
GLP-1R activation late in life alleviates the hallmarks of ageing and mimics the calorie restriction response. RNA-seq analysis of the livers of 20-month-old female C57BL/6 mice treated with vehicle or semaglutide for 3 months. *n* = 5 mice per group. **a**, Summary of the DEGs (red, upregulated in semaglutide-treated mice; blue, downregulated in semaglutide-treated mice; adjusted *P* (*P*_adj_) < 0.1). Wald test with Benjamini–Hochberg correction. FC, fold change. **b**,**c**, Gene Ontology (GO) pathway analysis for genes downregulated (**b**) and upregulated (**c**) in the livers of semaglutide-treated mice. Statistical analysis was performed using Fisher’s exact test. ER, endoplasmic reticulum; ERAD, ER-associated degradation; IFN, interferon. **d**, Transcription factor binding prediction analysis for DEGs. Statistical analysis was performed using Fisher’s exact tests. **e**,**f**, GO pathway analysis for genes upregulated in the liver with age and downregulated by semaglutide (**e**) and downregulated in the liver with age and upregulated by semaglutide (**f**). Statistical analysis was performed using the hypergeometric test. T_H_17, T helper 17. **g**,**h**, GO pathway analysis for genes downregulated (**g**) and upregulated (**h**) in the liver by calorie restriction (CR) and semaglutide. Statistical analysis was performed using the hypergeometric test. For **e**–**h**, data for the DEGs by ageing and calorie restriction were adapted from ref. ^42^ under a CC BY 4.0 licence.

RNA-sequencing (RNA-seq) analysis confirmed that semaglutide treatment reduced the hallmarks of ageing (Fig. 3a and Supplementary Table 1). Pathway analysis of differentially expressed genes (DEGs) revealed that semaglutide downregulated lipid metabolism and inflammatory responses while upregulating adaptive immune response, insulin response and proteostasis (Fig. 3b,c). Transcription factor binding prediction analysis for DEGs suggested that semaglutide-induced transcriptional changes were influenced by key transcription factors that regulate lipid and glucose metabolism (*Srebf1*,* Hnf4a*,* Ppara*,* Pparg* and* Hif1a*), inflammation (*Nfkb1*, *Egr1*,* Stat3* and* Irf3*), apoptosis (*Trp53* and* Trp73*), proteostasis (*Xbp1*, *Atf4*), stress response (*Jun*,* Fosl1*,* Fos* and *Nfe2l2*) and longevity (*Foxo4*,* Foxo1*, *Foxo3* and* Sirt1*) (Fig. 3d). Comparing semaglutide-regulated genes in old mice with ageing-regulated changes revealed that genes that increased with age and were suppressed by semaglutide were enriched in inflammation and lipid metabolism, while genes that decreased with age and were induced by semaglutide were enriched in adaptive immune response, DNA repair and glucose/insulin response^42^ (Fig. 3e,f). Furthermore, comparing semaglutide’s effects with calorie restriction, we observed a shared impact: genes suppressed by both calorie restriction and semaglutide were enriched in inflammation and lipid metabolism, while genes induced by both treatments were enriched in adaptive immune response, glucose/insulin response and proteostasis^42^ (Fig. 3g,h). Together, these data indicate that GLP-1R activation late in life mimics the beneficial effects of calorie restriction and effectively alleviates the hallmarks of ageing.

## Genetic regulators of ageing

**Fig. 4:**
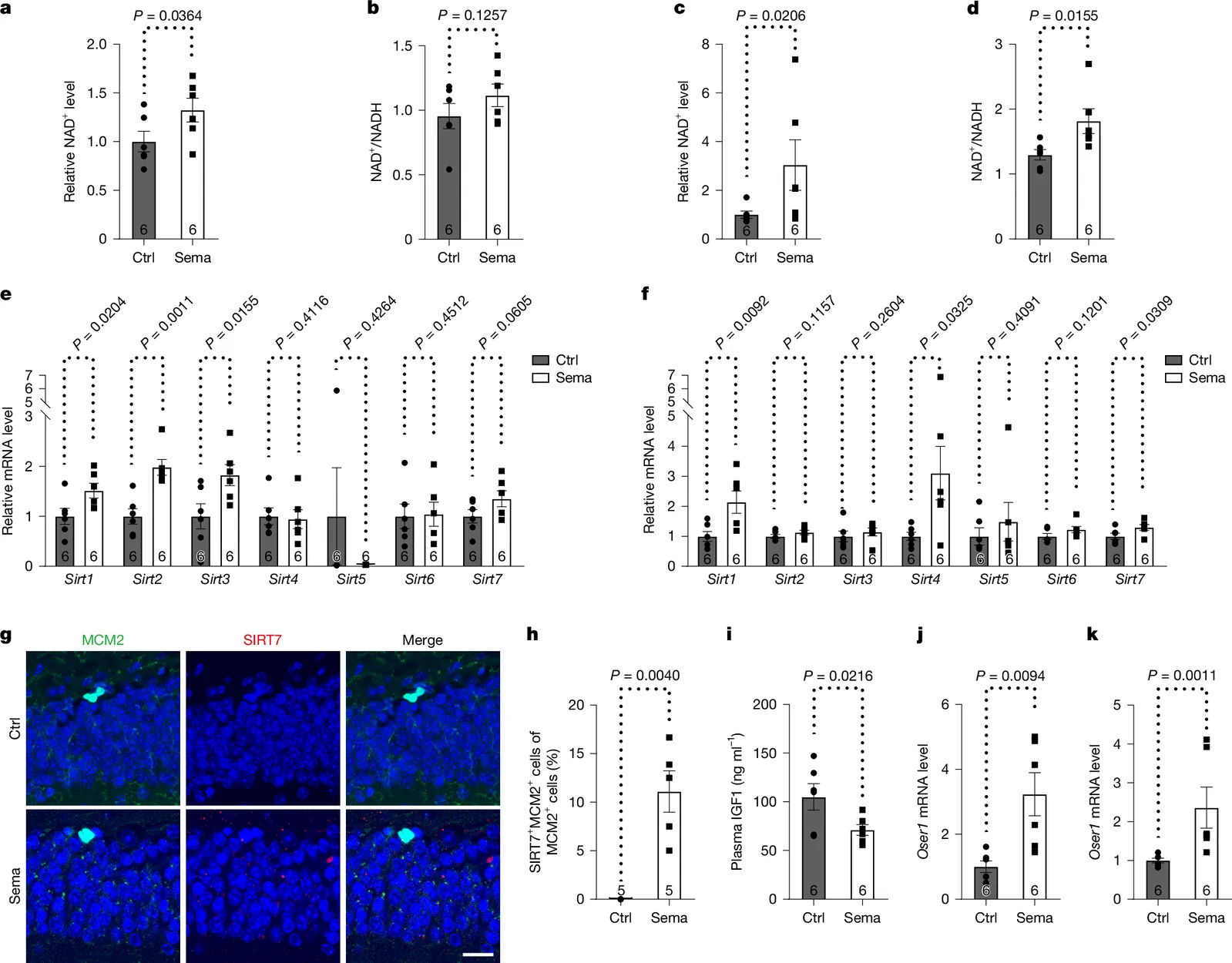
GLP-1R activation late in life modulates the genetic regulators of ageing. Comparison of 20-month-old female C57BL/6 mice treated with vehicle or semaglutide for 3 months. **a**–**d**, Relative NAD^+^ levels (**a**,**c**) and the ratio of NAD^+^/NADH (**b**,**d**) in the liver (**a**,**b**) and the muscle (**c**,**d**). **e**,**f**, Quantitative PCR with reverse transcription (RT–qPCR) analyses of *Sirt1–7* in the liver (**e**) and the muscle (**f**). **g**,**h**, Immunostaining (**g**) and quantification (**h**) of MCM2^+^ cells expressing SIRT7 in the dentate gyrus. Blue, DAPI; red, SIRT7; green, MCM2. Scale bar, 25 µm. **i**, Enzyme-linked immunosorbent assay (ELISA) analyses of IGF1 levels in the plasma. **j**,**k**, RT–qPCR analysis of *Oser1* in the liver (**j**) and the muscle (**k**). Data are mean ± s.e.m. *P* values are included in figures and source data. The statistical tests used are described in source data. Sample sizes (the numbers of mice) are shown in the figures. Source data

Ageing is regulated by conserved genetic factors, including the sirtuin family of nicotinamide adenine dinucleotide (NAD^+^)-dependent deacetylases and the insulin–IGF1 pathway^43^. During ageing, the NAD^+^ levels are reduced^31^ and the expression of sirtuins is also reduced^25,28–30,33,44^, while calorie restriction reduces the levels of IGF1^45^ and induces the expression of sirtuins, which mediate aspects of the calorie restriction response^8,9^. Semaglutide treatment increased the levels of NAD^+^ (Fig. 4a–d), induced the expression of several sirtuins (Fig. 4e–h) and reduced the levels of IGF1 (Fig. 4i). Transcription-factor-binding prediction analysis for DEGs also revealed that semaglutide-induced transcriptional changes were influenced by SIRT1 and FOXOs—transcription factors downstream of the insulin–IGF1 pathway (Fig. 3d). Semaglutide induced the expression of *Oser1*, encoding a conserved FOXO-regulated protein that increases lifespan in multiple species^46^ (Fig. 4j,k). These results are consistent with the notion that GLP-1R activation late in life functions as a calorie restriction mimetic that impinges on the nutrient sensors and genetic regulators of ageing.

## Comparison with calorie restriction

**Extended Data Fig. 7:**
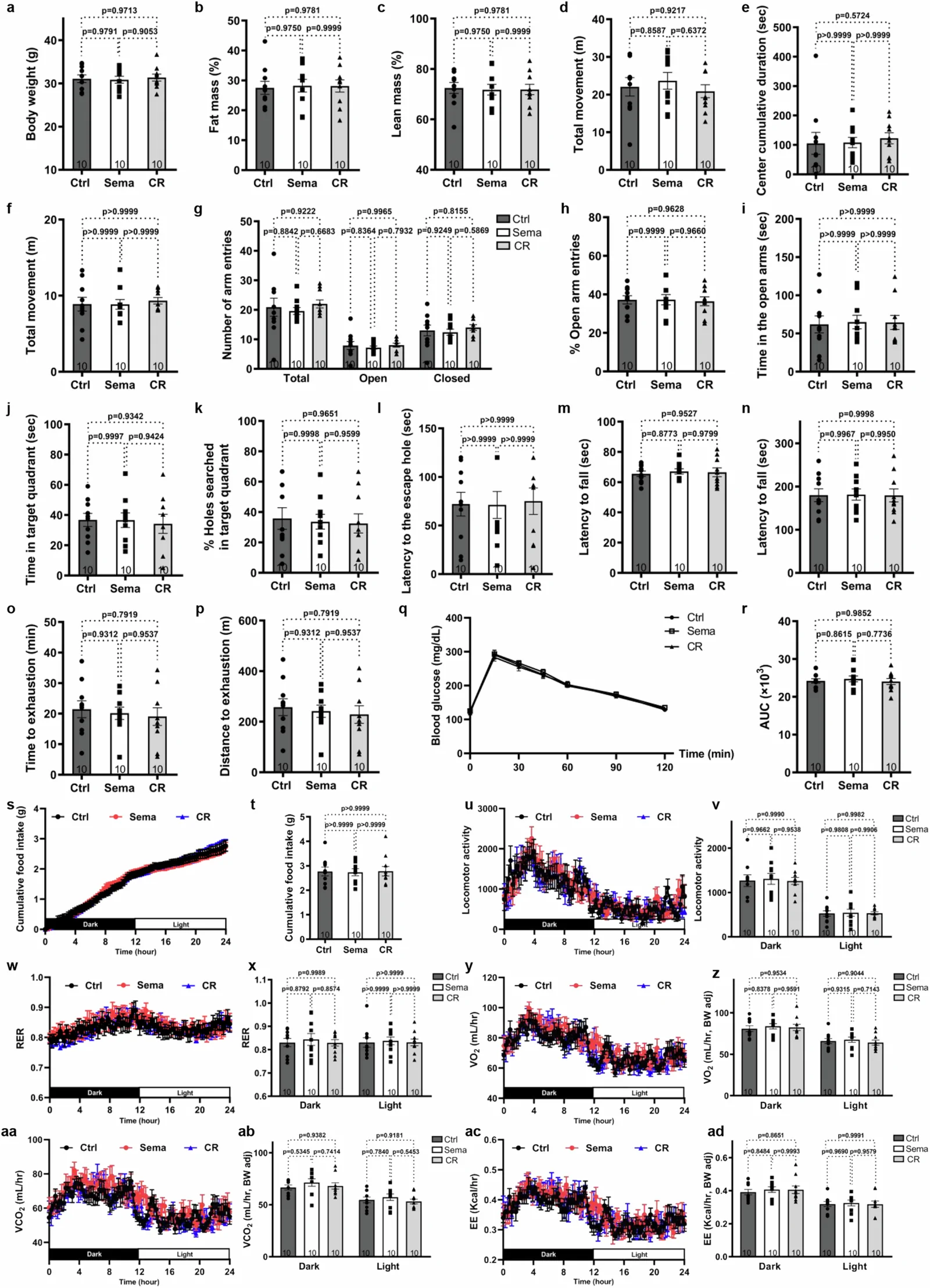
Baseline physiological measures in the longitudinal study. A longitudinal study of 20-month-old female C57BL/6 mice treated with vehicle, semaglutide, or calorie restriction for 5 months. Data shown are baseline physiological measures. a, Body weight. b, c, The percentage of fat mass (b) and lean mass (c). d, e, Total movement (d) and centre cumulative duration (e) in the open field test. f-i, Total movement (f), number of arm entries (g), the percentage of open arm entries (h), time in the open arms (i) in the elevated plus maze test. j-l, Time in target quadrant (j), percentage of holes searched in target quadrant (k), latency to the escape hole (l) in the Barnes maze test. m, Latency to fall in the rotarod test. n, Latency to fall in the inverted screen test. o, p, Time (o) and distance to exhaustion (p) in the treadmill exhaustion test. q, r, Blood glucose (q) and the area under the curve (AUC) (r) in the glucose tolerance test. s-ad, Measurements in metabolic cage. s, t, Cumulative food intake (s) and summarized (t). u, v, Locomotor activity (u) and summarized by light and dark cycles (v). w, x, Respiratory exchange ratio (w) and summarized by light and dark cycles (x). y, z, Oxygen consumption absolute value (y) and summarized by light and dark cycles after adjustment using ANCOVA with body weight as a covariate (z). aa, ab, Carbon dioxide production absolute value (aa) and summarized by light and dark cycles after adjustment using ANCOVA with body weight as a covariate (ab). ac, ad, Energy expenditure absolute value (ac) and summarized by light and dark cycles after adjustment using ANCOVA with body weight as a covariate (ad). Data are shown as mean ± SEM. P values are included in figures and source data. Statistical tests are included in source data. Sample sizes (the numbers of mice) are included in figures. Source data

**Extended Data Fig. 8:**
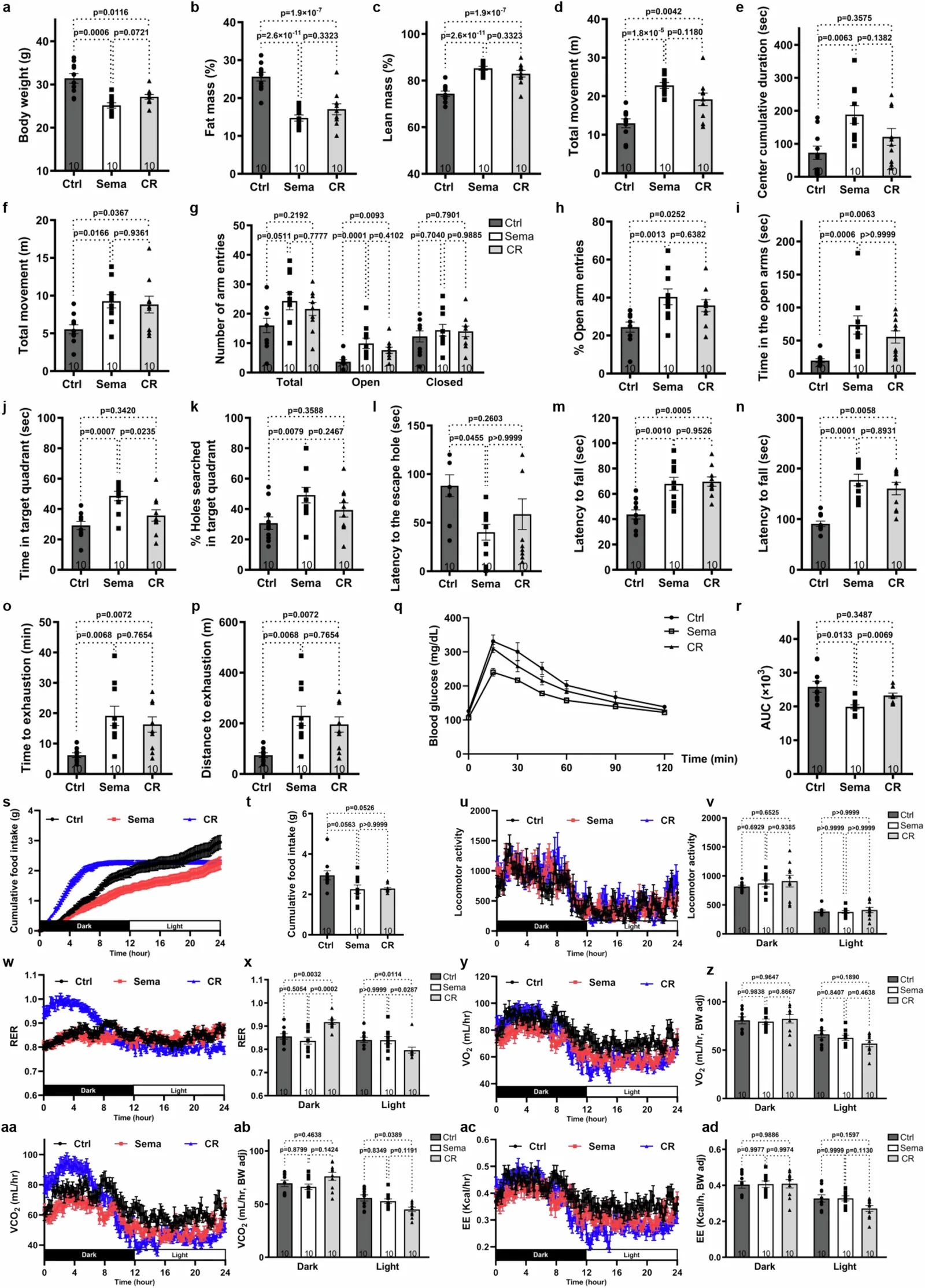
Physiological measures after 2 months of treatment in the longitudinal study. A longitudinal study of 20-month-old female C57BL/6 mice treated with vehicle, semaglutide, or calorie restriction for 5 months. Data shown are physiological measures after 2 months of treatment. a, Body weight. b, c, The percentage of fat mass (b) and lean mass (c). d, e, Total movement (d) and centre cumulative duration (e) in the open field test. f-i, Total movement (f), number of arm entries (g), the percentage of open arm entries (h), time in the open arms (i) in the elevated plus maze test. j-l, Time in target quadrant (j), percentage of holes searched in target quadrant (k), latency to the escape hole (l) in the Barnes maze test. m, Latency to fall in the rotarod test. n, Latency to fall in the inverted screen test. o, p, Time (o) and distance to exhaustion (p) in the treadmill exhaustion test. q, r, Blood glucose (q) and the area under the curve (AUC) (r) in the glucose tolerance test. s-ad, Measurements in metabolic cage. s, t, Cumulative food intake (s) and summarized (t). u, v, Locomotor activity (u) and summarized by light and dark cycles (v). w, x, Respiratory exchange ratio (w) and summarized by light and dark cycles (x). y, z, Oxygen consumption absolute value (y) and summarized by light and dark cycles after adjustment using ANCOVA with body weight as a covariate (z). aa, ab, Carbon dioxide production absolute value (aa) and summarized by light and dark cycles after adjustment using ANCOVA with body weight as a covariate (ab). ac, ad, Energy expenditure absolute value (ac) and summarized by light and dark cycles after adjustment using ANCOVA with body weight as a covariate (ad). Data are shown as mean ± SEM. P values are included in figures and source data. Statistical tests are included in source data. Sample sizes (the numbers of mice) are included in figures. Source data

**Extended Data Fig. 9:**
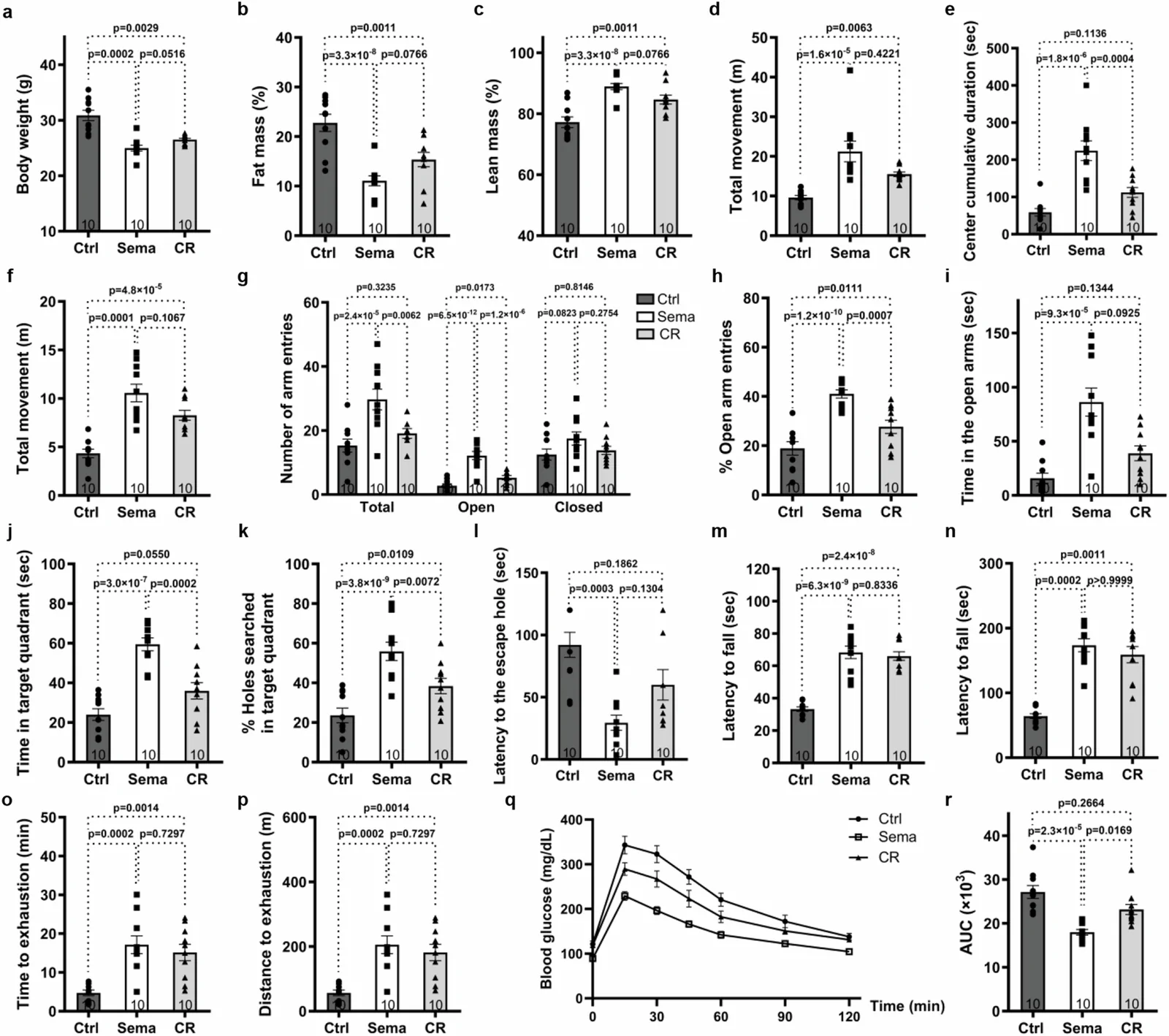
Physiological measures after 4 months of treatment in the longitudinal study. A longitudinal study of 20-month-old female C57BL/6 mice treated with vehicle, semaglutide, or calorie restriction for 5 months. Data shown are physiological measures after 4 months of treatment. a, Body weight. b, c, The percentage of fat mass (b) and lean mass (c). d, e, Total movement (d) and centre cumulative duration (e) in the open field test. f-i, Total movement (f), number of arm entries (g), the percentage of open arm entries (h), time in the open arms (i) in the elevated plus maze test. j-l, Time in target quadrant (j), percentage of holes searched in target quadrant (k), latency to the escape hole (l) in the Barnes maze test. m, Latency to fall in the rotarod test. n, Latency to fall in the inverted screen test. o, p, Time (o) and distance to exhaustion (p) in the treadmill exhaustion test. q, r, Blood glucose (q) and the area under the curve (AUC) (r) in the glucose tolerance test. Data are shown as mean ± SEM. P values are included in figures and source data. Statistical tests are included in source data. Sample sizes (the numbers of mice) are included in figures. Source data

We next compared directly semaglutide treatment with matched calorie restriction for slowing ageing-associated physiological decline. We treated 20-month-old female C57BL/6 mice daily with vehicle, semaglutide or 24% calorie restriction for 5 months, and assessed physiological measures at the baseline (Extended Data Fig. 7) and after 2 (Extended Data Fig. 8) or 4 months (Extended Data Fig. 9) of treatment. No differences were observed among groups at the baseline (Extended Data Fig. 7). Although mice treated with semaglutide and calorie restriction had a similar daily food intake, their feeding patterns differed markedly (Extended Data Fig. 8s,t). Calorie-restricted mice consumed their daily food allotment soon after feeding and then underwent a prolonged fasting period, whereas semaglutide-treated mice consumed food more gradually throughout the day, consistent with appetite suppression (Extended Data Fig. 8s). Correspondingly, calorie-restricted mice showed an increase in locomotor activity late in the light cycle when anticipating the next feeding, consistent with foraging behaviour, which was not observed in semaglutide-treated mice (Extended Data Fig. 8u). Calorie-restricted mice showed the expected dynamic changes in respiratory exchange ratio, with high values after feeding and lower values during the fasting phase (Extended Data Fig. 8w,x). Moreover, calorie-restricted mice showed comparable adjusted oxygen consumption, adjusted carbon dioxide production, adjusted energy expenditure during the dark cycle, but lower daytime adjusted carbon dioxide production and trends toward lower daytime adjusted oxygen consumption and adjusted energy expenditure (Extended Data Fig. 8y,z,aa,ab,ac,ad), consistent with the literature^12^. These results indicate that semaglutide and calorie restriction produce a similar reduction in overall calorie intake through distinct mechanisms and physiological states. Calorie restriction is associated with hunger-driven temporal behavioural and metabolic adaptations, whereas semaglutide reduces intake in the context of suppressed appetite and is therefore not accompanied by the same feeding-associated rhythms.

**Fig. 5:**
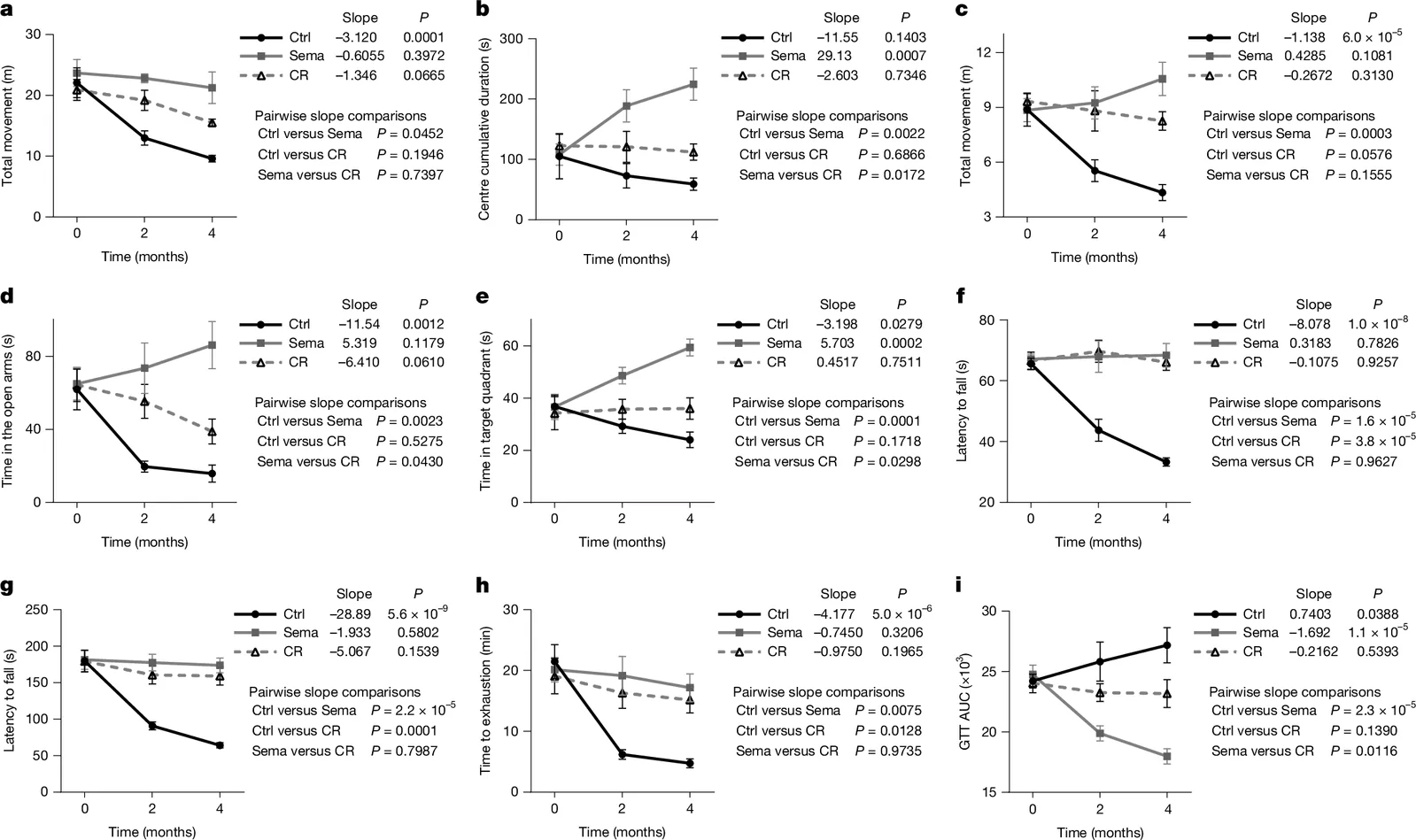
Direct comparison of GLP-1R activation and calorie restriction late in life. Comparison of 20-month-old female C57BL/6 mice treated with vehicle, semaglutide or calorie restriction for 5 months. Mice were assessed at the baseline or after 2 or 4 months of treatment. **a**,**b**, Total movement (**a**) and centre cumulative duration (**b**) in the open-field test. **c**,**d**, Total movement (**c**) and time in the open arms (**d**) in the elevated plus maze test. **e**, Time in target quadrant in the Barnes maze test. **f**, The latency to fall in the rotarod test. **g**, The latency to fall in the inverted screen test. **h**, The time to exhaustion in the treadmill exhaustion test.** i**, The glucose-tolerance test. Data are mean ± s.e.m. *P* values are included in figures and source data. The statistical tests used are described in the source data.* n* = 10 mice per group. Source data

**Extended Data Fig. 10:**
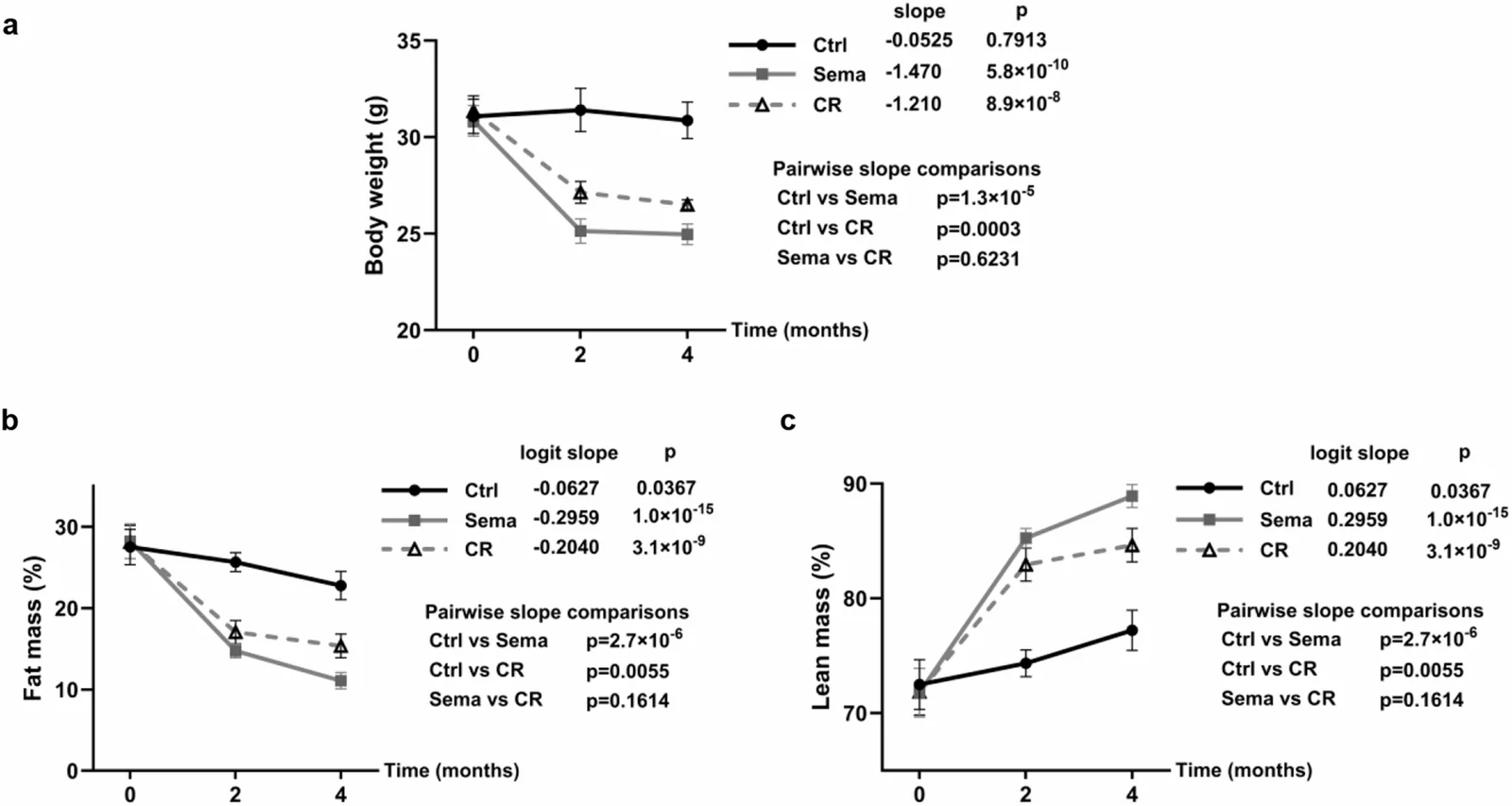
Direct comparison of GLP-1R activation and calorie restriction late in life. Comparison of 20-month-old female C57BL/6 mice treated with vehicle, semaglutide, or calorie restriction for 5 months. Mice were assessed at the baseline or after 2 or 4 months of treatment. a, Body weight. b, c, The percentage of fat mass (b) and lean mass (c). Data are shown as mean ± SEM. P values are included in figures and source data. Statistical tests are included in source data. n = 10 mice per group. Source data

Both semaglutide treatment and calorie restriction led to comparable body weight and fat loss (Extended Data Fig. 10a–c). Control mice exhibited progressive declines in locomotor activity and exploratory behaviour in novel environments, motor coordination, muscle function, glucose control and spatial memory over the course of the longitudinal study (Fig. 5). Semaglutide-treated mice showed significantly different trajectories from control mice across measures (Fig. 5). Semaglutide treatment preserved baseline function and produced trajectories comparable to those of calorie-restricted mice for total locomotor activity in the open-field (Fig. 5a) and elevated plus maze (Fig. 5c) tests, rotarod performance (Fig. 5f), inverted screen performance (Fig. 5g) and treadmill endurance (Fig. 5h). Notably, semaglutide treatment led to improvements above the baseline, as well as to more favourable trajectories in contrast to calorie restriction, in open-field centre time (Fig. 5b), Barnes maze performance (Fig. 5e) and glucose tolerance (Fig. 5i). These findings indicate that GLP-1R activation late in life recapitulates many functional benefits of calorie restriction by attenuating age-associated decline, while conferring additional benefits in exploratory drive, spatial memory and glucose control, suggesting effects beyond reduced calorie intake alone.

## Discussion

Preclinical studies in disease mouse models^47–50^ and clinical studies^1–5^ have revealed pleiotropic beneficial effects of GLP-1R activation, including improved glucose and weight control, and reduced cardiovascular, renal, hepatic and neurodegenerative disease burden. We found that GLP-1R activation late in life improved physiological function and extended lifespan in an ageing mouse model (20-month-old female C57BL/6) treated with a defined semaglutide regimen that reduced food intake by 24% (Fig. 1). Female mice were selected to minimize confounding effects of male aggression and injury, consistent with previous long-term ageing studies^12^. Within the end points monitored in the study, we did not observe adverse effects attributable to semaglutide. While GLP-1 medicines are widely used clinically, whether GLP-1R activation modulates ageing trajectories and lifespan in humans will require long-term clinical studies designed to evaluate ageing-related outcomes in older populations.

Our findings establish that GLP-1R activation late in life alleviates broadly ageing-associated decline and phenocopies the molecular and physiological benefits of calorie restriction. In aged female C57BL/6 mice, semaglutide treatment extended lifespan, improved physiological function (Fig. 1 and Extended Data Figs. 2 and 3), reduced hallmarks of ageing (such as stem cell attrition, inflammation, cellular senescence, genomic instability, mitochondrial dysfunction and loss of proteostasis; Figs. 2 and 3 and Extended Data Figs. 4–6) and modulated the genetic regulators of ageing and nutrient sensors in the same manner as calorie restriction (Fig. 4). The restoration of NSCs and neurogenesis in aged mice was particularly marked (Fig. 2), considering the emerging evidence that GLP-1 medicines have beneficial effects on neurodegeneration^5,50^. Direct comparison of semaglutide treatment with matched calorie restriction further showed comparable effects across several aspects of ageing-associated physiological decline (Fig. 5a,c,f,g,h), consistent with the idea that GLP-1 medicines can act as calorie restriction mimetics. As ageing is the biggest risk factor for numerous chronic diseases and calorie restriction slows ageing and ameliorates a broad spectrum of ageing-associated diseases, our findings raise the possibility that GLP-1 medicines may influence a wide array of seemingly unrelated diseases by slowing ageing.

While some semaglutide effects are similar to calorie restriction, there are mechanistic and functional differences. Calorie restriction was accompanied by hunger-driven behavioural and metabolic adaptations, whereas semaglutide suppressed food intake without eliciting the same compensatory feeding-associated metabolic rhythms (Extended Data Fig. 8s–ad). Our study also reveals that GLP-1R activation late in life exerts effects beyond those attributable to reduced calorie intake alone (Fig. 5b,e,i). Whereas calorie restriction generally maintained exploratory drive, spatial memory and glucose control near the baseline levels, semaglutide treatment significantly improved these outcomes above baseline, raising the possibility that GLP-1 medicines may not only slow but also reverse aspects of ageing-associated functional decline.

## Methods

### Mice

C57BL/6 mice (female, aged 20 months) were obtained from the National Institute on Aging. Mice were acclimatized for a week after arrival at the facility and before starting experimental treatments or baseline measurements. As described previously^12^, group housing is standard practice for calorie-restriction studies. Mice were group housed under a 12 h–12 h light–dark cycle at 20–26 °C and 30–70% humidity. Control and semaglutide-treated groups had ad libitum access to water and standard laboratory chow diet (LabDiet, Rodent Diet 5053). Calorie-restricted mice were provided with unlimited access to water and measured amount of food daily. Competition for food was minimized by placing food directly into the bottom of the cage, allowing individual mice to get a pellet. The degree of restriction was based on the mean reduction in food intake induced by semaglutide and not adjusted over time. Mice received daily subcutaneous injection of 10 nmol per kg semaglutide or an equal volume of saline. For the lifespan study, 39 mice received saline and 40 mice received semaglutide for the duration of the lifespan. For physiological, molecular and cellular studies, a separate cohort was treated for 3 months. Ten pairs for physiological assessments, five pairs for neural stem cell studies requiring tissue fixation, and six pairs for other molecular and cellular analyses. For longitudinal study, a separate cohort was treated with saline, semaglutide or calorie restriction for 5 months (10 mice per group). Measurements were assessed at the baseline, after 2 or 4 months of treatment.

For subcutaneous injection, we followed the standard operating protocol established by the animal care committee at the University of California, Berkeley. In brief, subcutaneous injections were performed daily using a 28-gauge needle in the dorsal subcutaneous region. To reduce discomfort, a new needle was used for each animal. To minimize local tissue irritation and the risk of chronic inflammation, the operator systematically rotated injection sites across the dorsal surface, ensuring no single site was used repeatedly in consecutive days. Animals were monitored daily for local reactions, including erythema, swelling, ulceration or tissue damage. No adverse effects were observed at any timepoint. On-site veterinarians were overseeing health status checks. All animal procedures were performed in accordance with the animal care committee at the University of California, Berkeley.

### Lifespan

Mouse lifespan was determined according to previous studies^12^. In brief, mice were examined daily for survival and signs of illness. Mice found dead were noted at daily inspection. Severely moribund mice were killed and recorded. A mouse was considered severely moribund if it showed one of the following clinical signs: (1) tumour formation; (2) untreatable wounds; (3) untreatable skin ulceration or abscesses; (4) severe rectal prolapse; (5) inability to eat or drink or any condition that interferes with the ability to eat or drink, lack of response after stimulation; (6) severe dehydration; (7) respiratory distress, agonal breathing, cyanosis; (8) paralysis/paresis; (9) uncontrollable haemorrhage; (10) unrelievable, progressive hypothermia; (11) body condition score <2 out of 5. Following established practice^12^, both mice euthanized or found dead were represented as deaths in the survival curves. No mice were removed from the study for reasons unrelated to age-associated decline and censored in the survival analysis.

### Body composition

Body composition of mice was measured using an EchoMRI-100V Body Composition Analyzer (EchoMRI). Fat mass and lean mass data were collected.

### Open-field test

As described previously^33^, mice were acclimatized to the testing room under normal light for 1 h before testing. Mice were placed at the centre of a plastic chamber (50 × 50 cm) and allowed to move freely for 15 min. Mouse activity was recorded using a digital video camera and analysed with EthoVision (XT14, Noldus Information Technology). The central zone of the chamber was defined by the distance to the wall equivalent to the length of the mouse.

### Elevated plus maze

As described previously^33^, mice were acclimatized to the testing room under red light for 1 h before testing. The maze consisted of two open arms and two closed arms. Mice were placed at the centre of the elevated plus maze, facing one of the closed arms, and allowed to explore freely for 10 min. Mouse activity was recorded using a digital video camera and the videos were analysed with EthoVision.

### Barnes maze test

As described previously^35^, the Barnes maze test was conducted using a 92 cm diameter circular acrylic platform with 20 evenly spaced holes (5 cm in diameter), one of which was the target hole with an escape box. The test included three phases: habituation (day 1), training (days 2–3) and probe trials (day 5). Mice were acclimatized to the behavioural testing room for 1 h before each session. On day 1, mouse was placed under a clear beaker at the maze centre for 30 s with noise (65 dB, 1 Hz metronome), and was guided toward the target hole and allowed 3 min to enter the escape box. If unsuccessful, mouse was placed directly into the box for 1 min with the noise turned off. On day 2–3, the mouse was placed under an opaque beaker for 10 s before release. After removal, noise was initiated and the mouse was given 2 min to enter the escape box. If unsuccessful, it was guided to the target hole by a clear beaker and given 3 min to escape into the box. If unsuccessful, mouse was placed directly into the box for 1 min with the noise turned off. On day 5, a single 2 min probe trial was conducted with noise turned on but without the escape box to assess spatial memory. All trials were recorded using a digital video camera and the videos (day 5) were analysed with EthoVision.

### Rotarod test

As described previously^22^, mice were put on a 3-cm-diameter rotating rod with an elevation of 44.5 cm (Rotamex-5, Columbus Instruments). During training days (days 1–3), mice were given habituated trials at a constant speed at 4 rpm for 60–300 s. On the test day (day 4), the mice were acclimatized to the testing room for 30 min before the experiment. The accelerated rotarod test consisted three trials where the speed increased from 4 to 40 rpm in 300 s. Each trial was separated by a 1 h interval. The fall latency was automatically recorded with infrared sensors, and the average fall latency was calculated.

### Inverted screen test

As described previously^35^, mice were acclimatized to the testing room for 30 min before the experiment. Mice were placed at the centre of the wire cage lid, then inverted and suspended 40–50 cm above a padded surface. Timing commenced once the mice assumed a fully inverted position. The latency to fall was recorded. Each mouse underwent three trials with a 1 h interval between trials, and the average fall latency was calculated.

### Treadmill exhaustion test

As described previously^24^, mice were placed onto the treadmill (Columbus Instruments, Exer-6M Open Treadmill). The mice were food deprived for 2 h before the training (days 1 and 2) and test (day 4). On day 1, mice were habituated on the stationary treadmill for 30 s, then run for 10 min with a stepwise increase in speed: 5 m min^−1^ (0–2.5 min), 6 m min^−1^ (2.5–5 min) and 8 m min^−1^ (5–10 min). On day 2, the protocol was repeated with speeds of 5 m min^−1^ (0–2.5 min), 7 m min^−1^ (2.5–5 min) and 10 m min^−1^ (5–10 min). On day 4, the mice were habituated for 30 s, and started running at 12 m min^−1^ for 40 min. The speed was then increased by 1 m min^−1^ every 10 min. Exhaustion was defined as the inability to resume running for at least 20 s despite gentle prodding. Time and distance were recorded.

### Glucose-tolerance test

As described previously^25^, mice were fasted for 14 h with free access to water. Blood glucose levels were measured from tail vein blood using a CONTOUR NEXT glucometer. Following the baseline measurement, mice received an intraperitoneal injection of 2 g per kg body weight d-glucose. Blood glucose levels were then measured.

### Metabolic cage

Metabolic parameters were measured using the Oxymax Comprehensive Lab Animal Monitoring System (CLAMS; Columbus Instruments).

### RNA-seq analysis

Total RNA of liver samples was extracted using the RNeasy Mini Kit (Qiagen). Poly(A)-enriched RNA-seq libraries were prepared by Novogene and sequenced on the NovaSeq X Plus (Illumina) system. Data analysis was processed on the Galaxy public server (https://usegalaxy.org). In brief, after removing low-quality reads and adaptor sequences, reads were aligned to the mouse genome (mm10) using HISAT2 (Galaxy v.2.2.1). Gene-level raw counts were generated using HTSeq (Galaxy v.2.0.5). Differential gene expression was analysed using DESeq2 (Galaxy v.2.11.40.8). Volcano plots were generated using the Volcano Plot tool (Galaxy v.0.0.7). Pathway enrichment analysis was performed using GSEAPy (v.1.0.6). Transcription factor enrichment analysis of DEGs with *P*_adj _< 0.1 was performed using TRRUST. Overlap between DEGs induced by semaglutide, aging^42^ or calorie restriction^42^ was processed for GO enrichment analysis using the enrichGO function in clusterProfiler (v.4.16.0).

### RT–qPCR

As described previously^40^, total RNA was extracted using TRIzol reagent (Invitrogen). cDNA was synthesized using the qScript cDNA SuperMix (Quanta Biosciences). Gene expression was determined by qPCR using the Eva qPCR SuperMix kit (BioChain Institute) on the ABI StepOnePlus system. All data were normalized to actin expression. The primer sequences are described in Supplementary Table 2.

### NAD^+^ and NAD^+^/NADH detection

Tissues were homogenized using PBS/bicarbonate/0.5% DTAB buffer. NAD^+^ and NADH levels were quantified using NAD/NADH-Glo (Promega). Acid-treated samples were used to measure NAD^+^, and base-treated samples were used to detect NADH. Luminescence intensity was measured using the SpectraMax i3 plate reader (Molecular Devices).

### ATP quantification

ATP was quantified using ATP Assay Kit (Sigma-Aldrich). The protein concentration was determined using the BCA Protein Assay (Thermo Fisher Scientific). ATP levels were normalized to protein content.

### Plasma IGF1 levels

As described previously^41^, plasma samples were pretreated with acid–ethanol extraction solution to release IGF1 from binding proteins. IGF1 concentrations were quantified using the mouse IGF1 ELISA Kit (Invitrogen).

### Western blotting

As described previously^41^, tissues were homogenized using RIPA buffer containing protease and phosphatase inhibitors. Protein concentration was determined by BCA Protein Assay Kit. Equal amounts of protein were separated by 12% SDS–PAGE gels and transferred to nitrocellulose membranes (Bio-Rad). After blocking with 5% BSA for 1 h at room temperature, the membranes were incubated overnight at 4 °C with primary antibodies: phospho-AKT (Ser473) antibody (CST, 9271, 1:1,000), AKT antibody (CST, 9272, 1:1,000), HSP90 antibody (CST, 4877, 1:1,000), p-eIF2α (Ser52) polyclonal antibody (Invitrogen, 44-728G, 1:1,000), eIF2α antibody (CST, 9722, 1:1,000), GRP78 antibody (Santa Cruz, 166490, 1:1,000), β-actin antibody (Santa Cruz, 47778, 1:2,000). The next day, membranes were incubated with horseradish-peroxidase-conjugated secondary antibodies (BioLegend, 406401, 405306, 1:4,000) for 2 h at room temperature. Bands were visualized with enhanced chemiluminescence substrate (PerkinElmer, NEL103001EA) using iBright CL1500 Imaging System (Invitrogen). Quantitative analysis was performed using Fiji/ImageJ (v.1.54p).

### Immunostaining

Cryosections were fixed, permeabilized, blocked and incubated with primary antibodies: anti-CD68 antibody (BioLegend, 137001, 1:200), anti-IL-6 antibody (CST, 12912S, 1:200), PE-CD11b antibody (BioLegend, 101208, 1:200), FITC-CCR2 antibody (BioLegend, 150607, 1:100), anti-γ-H2AX antibody (CST, 2577, 1:200) at 4 °C overnight, followed by incubation with secondary antibodies to the CD68, IL-6 and γ-H2AX antibodies (Thermo Fisher Scientific, SA5-10018 (1:500), A-11036 (1:500), A32731 (1:2,000)) at room temperature for 2 h. DAPI was used for nuclear staining. Images were taken on the Zeiss LSM 880 confocal microscope. Five fields per mouse were randomly selected for analysis. Fiji/ImageJ was used for image quantification.

For staining of brain sections^26,33^, mice received intraperitoneal injections of BrdU (50 mg per kg body weight) once daily for 3 days and were euthanized on day 4. Mice were perfused with 10 ml of PBS containing 10 U ml^−1^ heparin, followed by 40 ml of PBS with 4% paraformaldehyde. The brains were carefully dissected and post-fixed overnight at 4 °C in PBS with 4% paraformaldehyde. The brains were transferred into PBS with 15% sucrose at 4 °C overnight, then moved into PBS with 30% sucrose at 4 °C overnight. Brains were sectioned coronally at 40 μm using cryomicrotome (Leica) and stored in cryoprotective medium. For immunostaining, brain sections were pretreated with 2 N HCl at 37 °C for 30 min (for BrdU), then blocked at room temperature for 2 h and incubated at 4 °C overnight with the following primary antibodies: anti-BrdU (Abcam, ab6326, 1:500), anti-DCX (Abcam, ab18723, 1:750), anti-HSP60 (CST, 12165, 1:500), anti-MCM2 (BD Biosciences, 610700, 1:250) and anti-SIRT7 (21st Century Biochemical, custom antibody, 1:500). The next day, the sections were incubated with the corresponding secondary antibodies (Thermo Fisher Scientific, A11006, A32731, A32733 and A-11029; 1:1,000).

For quantification, immunopositive cells in the granule cell and subgranular cell layer of the dentate gyrus were counted on every sixth coronal hemibrain section. The average number of positive cells per section was calculated and then multiplied by the total number of sections and multiplied by 2 to estimate the total cell counts for both dentate gyri. Double-positive cells were identified based on the co-localization of HSP60 or SIRT7 and MCM2 signals encircling the same DAPI-stained nucleus. The proportion of activated NSCs expressing HSP60 or SIRT7 was calculated as the ratio of HSP60^+^MCM2^+^ or SIRT7^+^MCM2^+^ double-positive cells to the total number of MCM2^+^ cells.

### SA-β-gal staining

SA-β-gal staining was conducted using the Senescence beta-Galactosidase Staining kit (CST, 9860). In brief, cryosections were fixed with the supplied fixative solution for 15 min at room temperature. After fixation and washing, the sections were immersed in a fresh β-gal staining solution and incubated at 37 °C overnight. Images were taken on the Zeiss Axio Imager M2 microscope. Five fields per mouse were randomly selected for analysis. Fiji/ImageJ was used for image quantification.

### Colony-forming unit assay

In total, 2 × 10^4^ bone marrow cells were resuspended in MethoCult GF M3434 medium (StemCell Technologies) and cultured in cell incubator. Colony-forming units (CFUs) were quantified on day 12. Bright-field images were taken on the Zeiss Axio Imager M2 microscope, and colony areas were measured using Fiji/ImageJ.

### Flow cytometry and cell sorting

For HSC analysis, bone marrow cells were obtained in staining media (PBS with 2% FBS) by crushing the long bones, and stained with APC/Cy7-conjugated lineage antibodies, KIT-APC, SCA1-PB, CD48-FITC and CD150-PE antibodies (BioLegend, 101226, 108424, 116223, 100222, 100414, 100714, 103224, 105812, 108120, 103404, 115904; 1:100) for 20 min at 4 °C. Lineage antibodies included MAC1 (CD11b), GR1 (Ly-6G/C), Ter119 (Ly-76), CD3, CD4, CD8a (Ly-2) and B220 (CD45R) (BioLegend). For analysing lineage-biased HSCs, bone marrow cells were stained with PerCP/Cy5.5-conjugated lineage antibodies, KIT-APC/Cy7, SCA1-PB, CD150-PE, CD135-APC and CD34-FITC antibodies (BioLegend, 101228, 108428, 116228, 100218, 100434, 100734, 103236, 105826, 108120, 115904, 135310, eBioscience, 11-0341-81; 1:100) for 20 min at 4 °C. For MitoSOX analysis, cells were incubated with 5 µM MitoSOX Red Mitochondrial Superoxide Indicator (Thermo Fisher Scientific) for 30 min at 37 °C in the dark after HSC staining (BioLegend, 101226, 108424, 116223, 100222, 100414, 100714, 103224, 105812, 108120, 115916; 1:100).

For lineage differentiation in the peripheral blood, blood was collected through the submandibular vein into EDTA-treated tubes (BD), and lysed with 500 µl of 1× BD FACS lysing solution (BD) for 5 min at room temperature. Lysis was stopped with 3 ml of PBS, and stained with MAC1-PE, GR1-FITC, B220-APC and CD3-PB (BioLegend, 101208, 108406, 103212, 100214; 1:100) for 20 min at 4 °C. All data were acquired using the LSRFortessa flow cytometer (BD) and analysed using FlowJo (v.10.9.0).

For HSC sorting, bone marrow cells were lysed with ACK buffer and enriched for KIT^+^ cells with KIT microbeads (Miltenyi Biotec). KIT-enriched cells were stained with APC-Cy7-conjugated lineage antibodies, KIT-APC, SCA1-PB, CD48-FITC and CD150-PE antibodies (BioLegend) for 20 min at 4 °C, and stained with propidium iodide (0.5 µg ml^−1^). Live HSCs were sorted using the FACSAria sorter (BD).

### Statistical analysis

The number of mice chosen for each experiment was based on the principle that the minimal number of mice is used to have sufficient statistical power and is comparable to published literature for the same assay performed. Mice were randomized to groups. Data collection and analysis of mice and tissue samples were performed by investigators blinded to the treatment of the animals, except for data collection for calorie-restricted mice, as their feeding regimens were distinguishable. Measurements were taken from distinct samples. Experiments in the study were repeated twice, except for Fig. 3. Findings in Fig. 3 were validated by experiments assessing hallmarks of ageing, genetic regulators of ageing and nutrient sensors in Figs. 2 and 4 and Extended Data Figs. 3–6. GraphPad Prism 10, Excel (v.16.84) and jamovi (v.2.7.26) were used for statistical analyses. Data were tested for normality using the Shapiro–Wilk test. Normally distributed data were tested for homoscedasticity using *F*-test or Brown–Forsythe test. For two-group continuous data, non-normally distributed data were analysed using the Mann–Whitney *U*-test. Student’s *t*-tests were used for data with equal variances and Welch’s* t*-test was used for data with unequal variances. For three-group continuous data, Kruskal–Wallis tests were used for non-normally distributed data, followed by Dunn’s multiple-comparison test, one-way ANOVA for normally distributed data with equal variance, followed by Tukey’s multiple-comparison test, and Welch’s ANOVA for normally distributed data with unequal variances, followed by Games–Howell’s multiple-comparison test. Covariate-adjusted analyses were performed using ANCOVA. Count data were analysed using generalized linear models with Poisson or negative binomial distribution when there was overdispersion. Percentage data were analysed using beta regression. GTT data were analysed using two-way repeated-measures ANOVA followed by Sidak-adjusted pairwise comparisons between groups at each timepoint. Lifespan data were analysed using the log-rank test. Categorical distribution data were analysed using the Fisher–Freeman–Halton exact test. Weekly body weight data were analysed using linear mixed-effects model, followed by Holm-adjusted pairwise comparisons between groups at each timepoint. Other longitudinal continuous data were analysed using linear mixed-effects models to assess ageing trajectories. Slopes for each group were determined and pairwise comparisons of slopes were performed with Tukey adjustment. Longitudinal percentage data were analysed using *β* mixed-effects models to assess ageing trajectories, with pairwise comparisons of slopes performed using Tukey adjustment. Data are presented as means and the error bars represent the s.e.m.

### Reporting summary

Further information on research design is available in the Nature Portfolio Reporting Summary linked to this article.

## Online content

Any methods, additional references, Nature Portfolio reporting summaries, source data, extended data, supplementary information, acknowledgements, peer review information; details of author contributions and competing interests; and statements of data and code availability are available at https://doi.org/10.1038/s41586-026-10940-7.

## Supplementary information

Supplementary Figure 1 Uncropped gels and blots.

Reporting Summary

Supplementary Table 1 RNA-seq analysis of the livers of 20-month-old female C57BL/6 mice treated with vehicle or semaglutide for 3 months.

Supplementary Table 2 PCR primers.

Peer Review File

## Source data

Source Data Fig. 1

Source Data Fig. 2

Source Data Fig. 4

Source Data Fig. 5

Source Data Extended Data Fig. 1

Source Data Extended Data Fig. 2

Source Data Extended Data Fig. 3

Source Data Extended Data Fig. 4

Source Data Extended Data Fig. 5

Source Data Extended Data Fig. 6

Source Data Extended Data Fig. 7

Source Data Extended Data Fig. 8

Source Data Extended Data Fig. 9

Source Data Extended Data Fig. 10

## Data Availability

Sequencing data have been deposited at the Gene Expression Omnibus (GSE338091). Uncropped gels and blots are provided in Supplementary Fig. 1. Source data are provided with this paper.
