# Supplementary material for: Late-life semaglutide treatment slows ageing and extends lifespan in female mice
Source: Nature. 2026 Sep 2;657(8131):469–76. doi: 10.1038/s41586-026-10940-7 (PMC13558073; doi:10.1038/s41586-026-10940-7)
Supplement: Supplementary file 1 [file 41586_2026_10940_MOESM1_ESM.pdf]

nature portfolio

https://doi.org/10.1038/s41586-026-10940-7

Supplementary information

Late-life semaglutide treatment slows ageing and extends lifespan in female mice

In the format provided by the authors and unedited

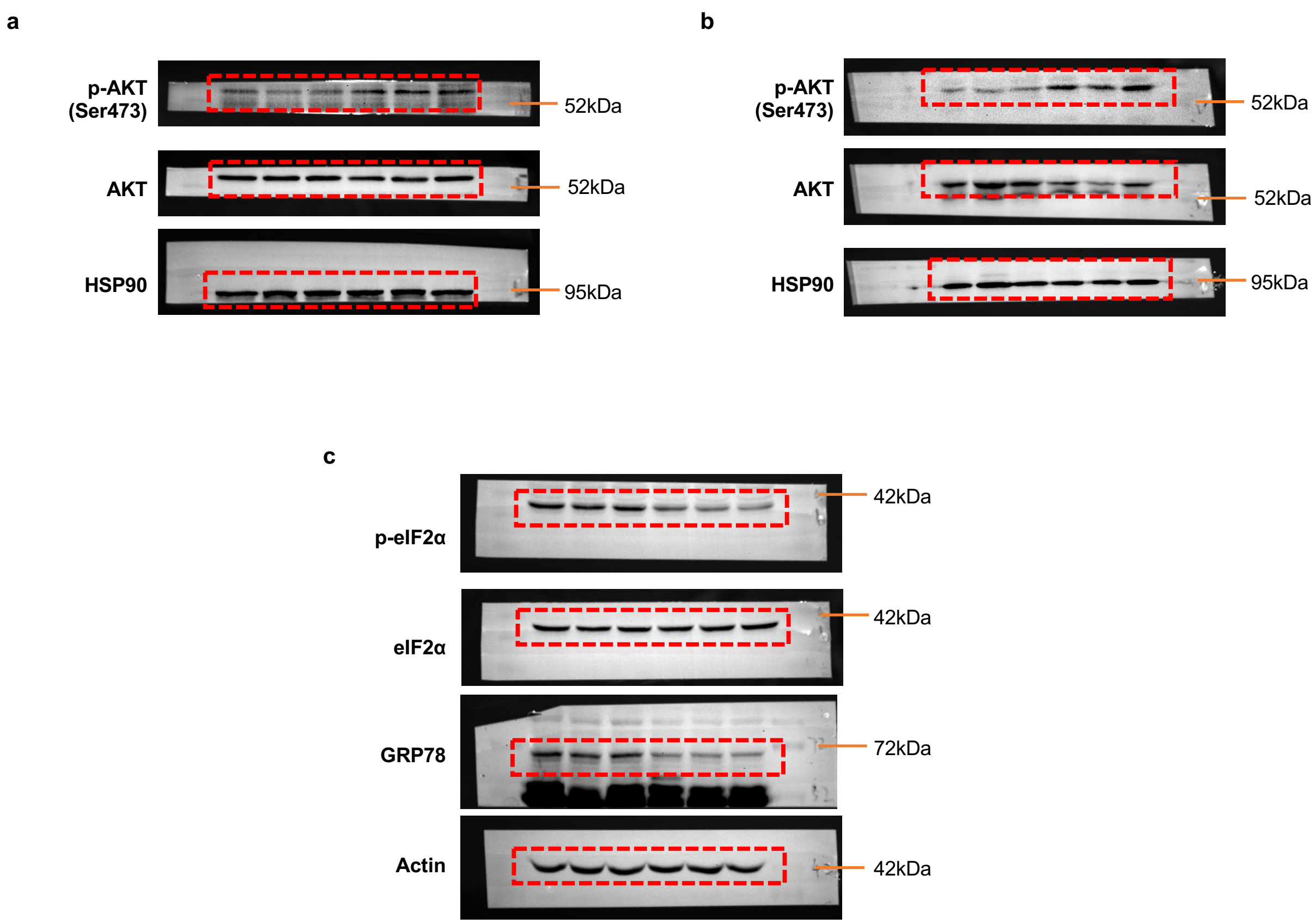

Supplementary Figure 1. Uncropped gels and blots.
a. Extend Data Figure 3j. HSP90 is loading control.
b. Extend Data Figure 3l. HSP90 is loading control.
c. Extend Data Figure 6e. Actin is loading control for GRP78, sample processing control for eIF2α.
