# Supplementary material for: Late-life semaglutide treatment slows ageing and extends lifespan in female mice
Source: Nature. 2026 Sep 2;657(8131):469–76. doi: 10.1038/s41586-026-10940-7 (PMC13558073; doi:10.1038/s41586-026-10940-7)
Supplement: Supplementary file 2 [file 41586_2026_10940_MOESM2_ESM.pdf]

Corresponding author(s): Danica Chen

Last updated by author(s): Jul 12, 2026

Reporting Summary

Nature Portfolio wishes to improve the reproducibility of the work that we publish. This form provides structure for consistency and transparency in reporting. For further information on Nature Portfolio policies, see our Editorial Policies and the Editorial Policy Checklist.

Statistics

For all statistical analyses, confirm that the following items are present in the figure legend, table legend, main text, or Methods section.

n/a	Confirmed	
☐	☒	The exact sample size (*n*) for each experimental group/condition, given as a discrete number and unit of measurement
☐	☒	A statement on whether measurements were taken from distinct samples or whether the same sample was measured repeatedly
☐	☒	The statistical test(s) used AND whether they are one- or two-sided<br>*Only common tests should be described solely by name; describe more complex techniques in the Methods section.*
☐	☒	A description of all covariates tested
☐	☒	A description of any assumptions or corrections, such as tests of normality and adjustment for multiple comparisons
☐	☒	A full description of the statistical parameters including central tendency (e.g. means) or other basic estimates (e.g. regression coefficient) AND variation (e.g. standard deviation) or associated estimates of uncertainty (e.g. confidence intervals)
☐	☒	For null hypothesis testing, the test statistic (e.g. *F*, *t*, *r*) with confidence intervals, effect sizes, degrees of freedom and *P* value noted<br>*Give P values as exact values whenever suitable.*
☒	☐	For Bayesian analysis, information on the choice of priors and Markov chain Monte Carlo settings
☒	☐	For hierarchical and complex designs, identification of the appropriate level for tests and full reporting of outcomes
☒	☐	Estimates of effect sizes (e.g. Cohen's *d*, Pearson's *r*), indicating how they were calculated

Our web collection on statistics for biologists contains articles on many of the points above.

Software and code

Policy information about availability of computer code

Data collection

EchoMRI™-100V Body Composition Analyzer (EchoMRI™).
Rotamex-5 (Columbus Instruments).
Exer-6M Open Treadmill (Columbus Instruments).
CONTOUR NEXT glucometer.
Oxymax Comprehensive Lab Animal Monitoring System (CLAMS; Columbus Instruments).
NovaSeq X Plus (Illumina).
ABI StepOnePlus system.
SpectraMax i3 plate reader (Molecular Devices)
iBright CL1500 Imaging System (Invitrogen).
Zeiss LSM 880 confocal microscope
Zeiss Axio Imager M2 microscope.
LSRFortessa Flow Cytometer (BD).
FACSAria sorter (BD).

Data analysis	Ethovision (XT14, Noldus Information Technology).<br>HISAT2 (Galaxy Version 2.2.1).<br>HTSeq (Galaxy Version 2.0.5).<br>DESeq2 (Galaxy Version 2.11.40.8).<br>Volcano Plot (Galaxy Version 0.0.7).<br>GSEAPy (v1.0.6).<br>TRRUST.<br>clusterProfiler (v4.16.0).<br>FIJI/ImageJ (v1.54p).<br>FlowJo(10.9.0).<br>GraphPad Prism 10.<br>Excel (16.84).<br>jamovi (2.7.26).

For manuscripts utilizing custom algorithms or software that are central to the research but not yet described in published literature, software must be made available to editors and reviewers. We strongly encourage code deposition in a community repository (e.g. GitHub). See the Nature Portfolio guidelines for submitting code & software for further information.

Data

Policy information about availability of data

All manuscripts must include a data availability statement. This statement should provide the following information, where applicable:

- Accession codes, unique identifiers, or web links for publicly available datasets
- A description of any restrictions on data availability
- For clinical datasets or third party data, please ensure that the statement adheres to our policy

Sequencing data are deposited at the Gene Expression Omnibus (GSE338091). Source data are provided with this paper. Uncropped gels and blots are provided in Supplementary Figure 1.

Research involving human participants, their data, or biological material

Policy information about studies with human participants or human data. See also policy information about sex, gender (identity/presentation), and sexual orientation and race, ethnicity and racism.

Reporting on sex and gender	N/A
Reporting on race, ethnicity, or other socially relevant groupings	N/A
Population characteristics	N/A
Recruitment	N/A
Ethics oversight	N/A

Note that full information on the approval of the study protocol must also be provided in the manuscript.

Field-specific reporting

Please select the one below that is the best fit for your research. If you are not sure, read the appropriate sections before making your selection.

☒ Life sciences ☐ Behavioural & social sciences ☐ Ecological, evolutionary & environmental sciences

For a reference copy of the document with all sections, see nature.com/documents/nr-reporting-summary-flat.pdf

Life sciences study design

All studies must disclose on these points even when the disclosure is negative.

Sample size	No statistical tests were used to pre-determine sample sizes. The number of mice chosen for each experiment was based on the principle that the minimal number of mice is used to have sufficient statistical power and is comparable to published literature for the same assays performed.
Data exclusions	No data exclusion
Replication	The data points in the graphs represent biological replicates. Number of biological replicates is described in the figures.
Randomization	Assessment of mice was randomized.

Blinding

Data collection and analysis of mice and tissue samples were performed by investigators blinded to the treatment of the animals, except for data collection for calorie restricted mice, because their feeding regimens were distinguishable.

Reporting for specific materials, systems and methods

We require information from authors about some types of materials, experimental systems and methods used in many studies. Here, indicate whether each material, system or method listed is relevant to your study. If you are not sure if a list item applies to your research, read the appropriate section before selecting a response.

Materials & experimental systems

n/a	Involved in the study	
☐	☒	Antibodies
☒	☐	Eukaryotic cell lines
☒	☐	Palaeontology and archaeology
☐	☒	Animals and other organisms
☒	☐	Clinical data
☒	☐	Dual use research of concern
☒	☐	Plants

Methods

n/a	Involved in the study	
☒	☐	ChIP-seq
☐	☒	Flow cytometry
☒	☐	MRI-based neuroimaging

Antibodies

Antibodies used

Mac1 APC/Cy7, BioLegend, Cat#101226, clone M1/70, dilution 1:100
Gr1 APC/Cy7 BioLegend, Cat#108424, clone RB6-8C5, dilution 1:100
TER-119 APC/Cy7, BioLegend, Cat#116223, clone TER-119, dilution 1:100
CD3 APC/Cy7, BioLegend, Cat#100222, clone 17A2, dilution 1:100
CD4 APC/Cy7, BioLegend, Cat#100414, clone GK1.5, dilution 1:100
CD8a APC/Cy7, BioLegend, Cat#100714, clone 53-6.7, dilution 1:100
B220 APC/Cy7, BioLegend, Cat#103224, clone RA3-6B2, dilution 1:100
c-Kit APC, BioLegend, Cat#105812, clone 2B8, dilution 1:100
Sca-1 PB, BioLegend, Cat#108120, clone D7, dilution 1:100
CD48 FITC, BioLegend, Cat#103404, clone HM48-1, dilution 1:100
CD150 PE, BioLegend, Cat#115904, clone TC15-12F12.2, dilution 1:100
Mac1 PerCP/Cy5.5, BioLegend, Cat#101228, clone M1/70, dilution 1:100
Gr1 PerCP/Cy5.5, BioLegend, Cat#108428, clone RB6-8C5, dilution 1:100
TER-119 PerCP/Cy5.5, BioLegend, Cat#116228, clone TER-119, dilution 1:100
CD3 PerCP/Cy5.5, BioLegend, Cat#100218, clone 17A2, dilution 1:100
CD4 PerCP/Cy5.5, BioLegend, Cat# 100434, clone GK1.5, dilution 1:100
CD8a PerCP/Cy5.5, BioLegend, Cat#100734, clone 53-6.7, dilution 1:100
B220 PerCP/Cy5.5, BioLegend, Cat#103236, clone RA3-6B2, dilution 1:100
c-Kit APC/Cy7, BioLegend, Cat#105826, clone 2B8, dilution 1:100
CD135 APC, BioLegend, Cat#135310, clone A2F10, dilution 1:100
CD34 FITC, eBioscience™, Cat#11-0341-81, clone RAM34, dilution 1:100
Mac1 PE, BioLegend, Cat#101208, clone M1/70, dilution 1:100 for HSCs, 1:200 for liver
Gr1 FITC, BioLegend, Cat#108406, clone RB6-8C5, dilution 1:100
B220 APC, BioLegend, Cat#103212, clone RA3-6B2, dilution 1:100
CD3 PB, BioLegend, Cat#100214, clone 17A2, dilution 1:100
CD150 Alexa Fluor 488, BioLegend, Cat#115916, clone TC15-12F12.2, dilution 1:100
Anti-BrdU Antibody, Abcam, Cat# ab6326, clone BU1/75 (ICR1), dilution1:500
Goat anti-Rat IgG (H+L) Cross-Adsorbed Secondary Antibody, Alexa Fluor 488, Thermo Fisher Scientific, Cat# A-11006, dilution1:1000
anti-Doublecortin Antibody, Abcam, Cat# ab18723, dilution1:750
Goat anti-Rabbit IgG (H+L) Highly Cross-Adsorbed Secondary Antibody, Alexa Fluor Plus 488, Thermo Fisher Scientific, Cat# A32731, dilution1:1000 for brain sections and 1:2000 for liver sections.
anti-SIRT7, 21st Century Biochemical custom antibody, dilution1:500
Goat anti-Rabbit IgG (H+L) Highly Cross-Adsorbed Secondary Antibody, Alexa Fluor Plus 647, Thermo Fisher Scientific, Cat#A32733, dilution1:1000
anti-BM28 (MCM2), BD Biosciences, Cat#610700, clone 46/BM28, dilution1:250
Goat anti-Mouse IgG (H+L) Highly Cross-Adsorbed Secondary Antibody, Alexa Fluor™ 488, Thermo Fisher Scientific, Catalog # A-11029, dilution 1:1000
anti-HSP60, CST, Cat#12165, clone D6F1, dilution1:500
anti-CD68 Antibody, BioLegend, Cat#137001, clone FA-11, dilution1:200
Goat anti-Rat IgG (H+L) Cross-Adsorbed Secondary Antibody, DyLight 488, Thermo Fisher Scientific, Cat# SA5-10018, dilution1:500
anti-IL-6 Antibody, CST, Cat#12912S, clone D5W4V, dilution1:200
Goat anti-Rabbit IgG (H+L) Highly Cross-Adsorbed Secondary Antibody, Alexa Fluor 568, Thermo Fisher Scientific, Cat# A-11036, dilution1:500
FITC anti-CD192 (CCR2) Antibody, BioLegend, Cat#150607, clone SA203G11, dilution1:100
Phospho-Histone H2AX (Ser139) Antibody, CST, Cat#2577, dilution1:200
phospho-Akt (Ser473) Antibody, CST, Cat#9271, dilution1:1000
Akt Antibody, CST, Cat#9272, dilution1:1000

HSP90 Antibody, CST, Cat#4877, clone C45G5, dilution1:1000
Phospho-EIF2S1 (Ser52) polyclonal Antibody, Invitrogen, Cat#44-728G, dilution1:1000
eIF2 alpha Antibody, CST, Cat#9722, dilution1:1000
β-Actin Antibody, Santa Cruz, Cat#47778, clone C4, dilution1:2000
HRP Donkey anti-rabbit IgG (minimal x-reactivity) Antibody, Biolegend, Cat#406401, clone Poly4064, dilution1:4000
GRP78 Antibody, Santa Cruz, Cat#sc-166490, clone E-4, dilution1:1000
HRP Goat anti-mouse IgG (minimal x-reactivity) Antibody, Biolegend, Cat#405306, clone Poly4053, dilution1:4000

Validation

All antibodies are from commercially available sources except for SIRT7 and have been validated and reported in publications (Mohrin, Science 201S; Luo, Cell Reports 2019, Ohkubo, Cell reports 2022,Wang, Cell metabolism 2023).

Animals and other research organisms

Policy information about studies involving animals; ARRIVE guidelines recommended for reporting animal research, and Sex and Gender in Research

Laboratory animals

20-month-old C57BL/6 female mice were obtained from the National Institute on Aging. Mice were group-housed on a 12:12 h light:dark cycle at 20-26°C and 30-70% humidity. Control and semaglutide-treated groups had ad libitum access to water and standard laboratory chow diet (LabDiet, Rodent Diet 5053). Calorie restricted mice were provided with unlimited access to water and measured amount of food daily. On-site veterinarians were overseeing health status checks. All animal procedures were in accordance with the animal care committee at the University of California, Berkeley.

Wild animals

This study did not use wild animals.

Reporting on sex

Experiments were done in female mice.

Field-collected samples

This study did not involve samples collected from the field.

Ethics oversight

All animal procedures were in accordance with the animal care committee at the University of California, Berkeley.

Note that full information on the approval of the study protocol must also be provided in the manuscript.

Plants

Seed stocks

N/A

Novel plant genotypes

N/A

Authentication

N/A

Flow Cytometry

Plots

Confirm that:

☒ The axis labels state the marker and fluorochrome used (e.g. CD4-FITC).

☒ The axis scales are clearly visible. Include numbers along axes only for bottom left plot of group (a 'group' is an analysis of identical markers).

☒ All plots are contour plots with outliers or pseudocolor plots.

☒ A numerical value for number of cells or percentage (with statistics) is provided.

Methodology

Sample preparation

Bone marrow cells were obtained by crushing the long bones with staining media (sterile PBS without calcium and magnesium supplemented with 2% FBS). Cells were stained with antibodies at 4°C for 20 minutes. Samples were washed with staining media before flow cytometry analysis.

Instrument

LSRFortessa Flow Cytometer (BD)

Software

FlowJo (10.9.0)

Cell population abundance	HSCs are very rare cell population, around 0.01% of bone marrow cells.
Gating strategy	We first used FSCA/FSCW to gate singlets and then used FSC/SSC to gate non debris. Single color controls were used for compensation. FMO panels were used for gating. Gating strategy was included in Figures.

☒ Tick this box to confirm that a figure exemplifying the gating strategy is provided in the Supplementary Information.
