# Supplementary material for: Late-life semaglutide treatment slows ageing and extends lifespan in female mice
Source: Nature. 2026 Sep 2;657(8131):469–76. doi: 10.1038/s41586-026-10940-7 (PMC13558073; doi:10.1038/s41586-026-10940-7)
Supplement: Supplementary file 3 [file 41586_2026_10940_MOESM5_ESM.pdf]

nature portfolio

Peer Review File

Late-life Semaglutide Treatment Slows Aging and Extends Lifespan in Female Mice

Corresponding Author: Professor Danica Chen

Any redactions in this file are there to maintain patient confidentiality, the confidentiality of unpublished data, or to remove third-party material.

This file contains all reviewer reports in order by version, followed by all author rebuttals in order by version.

Version 1:

Reviewer comments:

Referee #1

(Remarks to the Author)
This paper describes studies of a GLP-1 receptor agonist, semaglutide, on parameters of aging in mice. The underlying hypothesis driving this work is that effects of semaglutide to reduce feeding mimics the effects of dietary caloric restriction that has been shown to promote longevity. The principal findings reported are that: 1) semaglutide administration to 20 week old mice extended lifespan by ~ 14 % compared to control treated mice; 2) semaglutide mice performed significantly better in tests of aging such as locomotor activity, muscle strength, and cognition; 3) semaglutide improved glucose tolerance and respiratory capacity; 4) semaglutide had salutary effects on established cellular and tissue markers of aging including stem cell dynamics, proteostasis, inflammation and mitochondrial function. The authors conclude from these results that semaglutide phenocopies the effects of caloric restriction to extend the lifespan and healthspan of mice.

This is an interesting set of observations on a timely and potentially impactful topic. GLP-1-based drugs are available and more refined versions with greater potency and ease of use are in development. These compounds are highly effective for treating diabetes, mitigate several co-morbidities of obesity such as NAFLD and obstructive sleep apnea, and are now the most potent weight loss drugs on the market. The demonstration that chronic treatment with semaglutide extends lifespan and quality adds a novel and important therapeutic application. The data presented in this paper make this demonstration in a convincing manner. The experiments are straightforward, and most have been used in previous studies of aging and caloric restriction. The sample sizes are adequate and the key effect sizes (e.g. longevity, weight loss) are meaningful. This is a paper that will have broad interest.

The short-coming of this research is that these outcomes are mostly predictable. It is well known that semaglutide reduces food intake and body weight in rodents, humans and non-human primates. Thus, it is not surprising that medical caloric restriction has many features of dietary caloric restriction. In fact, it would have been surprising if semaglutide did not share many hallmarks of chronic food restriction. The study is essentially descriptive, although the phenomenon in question is interesting. It seems likely that many of the outcome measures here are a function of negative energy balance, although it is possible that some were mediated directly at the GLP-1 receptor in specific tissues; this question is not explored. Different approaches could have added depth to the results by trying to identify differences between semaglutide and caloric restriction, e.g. a matched calory restriction comparator, or even the use of doses of semaglutide that do not cause substantial weight loss.

As a minor point it was difficult to find a description of the semaglutide dose and treatment schedule in the text.

[redacted]

Referee #2

(Remarks to the Author)
This study shows that daily subcutaneous injection of semaglutide (10 nmol/kg) in 20-month-old C57BL/6J female mice over three months modulates a range of core molecular, cellular, and functional metabolic biomarkers, indicating its potential to

alleviate various hallmarks of aging. However, this requires further validation because many conclusions are based solely on mRNA expression levels, and functional products are not always measured. By continuing semaglutide injections until the end of the experimental period (about 28 months of age, which is the age at which the last control saline-injected animal died—quite young for this sex and strain), the authors demonstrate that this GLP-1 receptor agonist may extend median lifespan by approximately 13% in this sex and strain.

This work builds on earlier findings that show similar beneficial effects for semaglutide or other GLP-1 receptor activators, potentially indicating that this compound can also extend lifespan under "normal aging," unlike previous studies that demonstrated this effect in mouse models of disease.
Overall, the manuscript is written clearly and is well-organized. The experiments designed, techniques used, and general methodology are solid. However, several aspects need revision, clarification, or expansion, both in terms of experimental or analytical validation and discussion enrichment, to effectively test the author's hypothesis and support their main conclusion that semaglutide can serve as a calorie restriction mimetic, slowing aging and extending lifespan. These issues are outlined below.

General comments

1. It is recommended to define all abbreviations used throughout the manuscript at their first mention, regardless of how common they might be in the literature.

2. It is recommended to indicate the sex and strain of the mice used in each and all figure legends.

Title section

1. The phrase "Slows Aging" may be too broad for this article because no longitudinal or baseline measures (before starting experimental treatments) have been taken for any variables except body weight. Additionally, healthspan cannot be assessed through endpoint analysis since it doesn't allow determination of when impairments begin. Furthermore, only "old" C57BL/6J female mice were used in this study, so the results cannot be generalized to other sexes, strains, or age groups. Therefore, the title should be rephrased to more accurately reflect what was actually studied.

Abstract section

1. It is common practice to exclude references in the abstract of original articles, as their focus should be on what was done, the main results, and the conclusions, rather than on citing other work. I recommend removing references 1-11 from the abstract.

2. Please indicate the sex, strain, age, and number of mice used in this study, as well as the duration of treatment with semaglutide.

Introduction section

1. In the third sentence, the authors state: "Calorie restriction engages several evolutionarily conserved signaling pathways, such as sirtuins and insulin/IGF-1 signaling, to elicit a beneficial response." Please review the spelling of the word "illicit" (Did you mean "elicit"?).

Results section

1. At the beginning of the results section, the authors state: "We treated old (20 months old) mice daily with or without semaglutide, a GLP-1R agonist." Please specify what "treated" means (such as subcutaneous injection), as well as the sex and strain of the mice, and how long the animals received the semaglutide injections.

2. Fig. 1a: The median and maximum lifespan of control saline-injected animals is quite short for this sex and strain (https://doi.org/10.1007/s11357-024-01226-9; https://doi.org/10.1016/j.cmet.2021.08.013). Could the daily injections for approximately 260 days influence their normal lifespan? Alternatively, might this result be due to other factors, such as genetic or environmental conditions related to the facility where the experiment was conducted? Regardless, the lifespan results should be discussed in the context of existing literature. Ideally, the lifespan of the control saline-injected group should be compared with that of an intact control group kept under the same conditions but without saline injections. Additionally, the methods section describes conditions that classify an animal as severely moribund, justifying euthanasia. Please specify (1) how many animals experienced each of these conditions, (2) how these causes of death were distributed among the experimental groups (were they evenly distributed?), and (3) the ages at which these causes of death occurred. If animals that died from these reasons were included in the lifespan analysis, clarify whether they were censored or not. If censored, explain how this is represented in the Kaplan-Meier curves and accounted for in survival estimates. Also, state the statistical test used for survival analysis.

3. Subsection "GLP-1R activation extends healthspan" and Fig. 1. As noted above, healthspan cannot be assessed through endpoint analysis and requires longitudinal data; otherwise, it is impossible to determine when the onset of impairments occurs. In addition, a major general limitation of the experimental design of this study is that baseline measures have not been performed. This precludes one from distinguishing whether the treatment with semaglutide actually delayed the onset of the impairments observed in the control animals and/or whether the animals in the semaglutide group were different from

the control ones before initiating the treatment. At most, with this experimental design, the authors can state that "X" variable was improved by the treatment with semaglutide for 3 months (and acknowledging the limitation that they do not have baseline measurements). Therefore, both the title of the subsection "GLP-1R activation extends healthspan" and the last sentence of that subsection in which the authors state "Together, these data suggest that semaglutide treatment extends healthspan" Statement "semaglutide treatment extends healthspan", should be revised to accurately reflect their results.

4. Regarding the results of the behavioral test presented in Fig. 1. Could the increases in locomotor and exploratory behaviors in the semaglutide-treated mice be related with foraging behavior?

5. Fig. 1: Were the same 10 animals measured in the tests shown in Fig. 1b-q? Was this a separate group of animals or were they a subgroup of the animals used in the lifespan analysis? How were the animals selected if they were a subgroup of the animals used in the lifespan analysis? What was the total number of animals used in this study, including all tests performed and sample collection? Please describe this information in detail in methods or/and results section.

6. Fig. S1a: The authors show that after ~10 days on treatment, a reduction in body weight is observed in the semaglutide-treated animals compared to the control group. Was food consumption inhibited or reduced by the treatment with semaglutide? Please provide the food consumption trajectories in each experimental treatment, as this information is critical to understand the extent to which the reduction in body weight is explained by reduced feed intake or other mechanisms and, thus, assess the feasibility of the authors' hypothesis regarding that semaglutide can act as a calorie restriction mimetic. Ideally a pair-fed group or weight matched group should be involved to ascertain whether the effect is a drug effect or "simply" a caloric restriction effect.

7. Fig. S1a: Could the authors provide the body weight trajectories of each experimental group before starting the treatment? Please indicate if the trajectories of body weight correspond to all animals included in the lifespan analysis or if it is a subgroup of animals. It is also important to note that for the age-range shown in this figure (600-780 days of age), it would be expected that control animals start to show a modest decline in body weight (https://doi.org/10.1016/j.cmet.2021.08.013). Discussing these results in light of the available literature for C57BL/6 female mice is strongly recommended. Also, why do the authors choose to show the body weight trajectories in that age-range instead of the entire age-range covered in the lifespan analysis (600-860 days of age)? Why isn't there a statistical analysis associated with these body weight trajectories?

8. Fig. 1l,m: In parallel with the analyses of the absolute values of the rotarod and grip strength (sic) tests currently shown, please provide these data analyzed using ANCOVA including body weight as a covariate in the estimation of performance on these tests. This will inform whether the physical performance improvement in the semaglutide group is independent or mediated by body weight/lean mass.

9. Fig. S2: Based on the VO2 and energy expenditure values provided, mice treated with semaglutide would have increased metabolism. However, since the values are presented as ratios to body weight (Kcal/h/kg or ml/h/kg), the leaner semaglutide-treated animals may show apparently higher EE and VO2 only because of how this ratio is calculated and not because they are actually consuming more oxygen. Also, from a statistical standpoint, using the ratio Energy Expenditure/Body Weight can introduce bias if the relation between EE and BW includes an intercept different than zero (i.e., if it is not purely linear). Thus, it is recommended to present the absolute values of these outcomes (Kcal/h or ml/h) in parallel with data adjusted by lean mass (physiologically more relevant than body weight) or body weight as covariates through an appropriate statistical method such as ANCOVA (see for example DOI https://doi.org/10.1038/s41586-024-08026-3). Such method allows to accurately compare experimental groups with different body weight or lean mass without introducing the bias of the method that uses the ratios. On the other hand, in addition to the trajectories of food consumption, please provide the carbon dioxide production levels, the respiratory exchange ratio and locomotor activity obtained with the CLAMS system, since these are fundamental measures to interpret the energy balance and type of substrate being used (fat vs carbohydrate). In addition, the findings emerging from these variables should be discussed more in depth, as they are critical for the authors' hypothesis regarding that semaglutide acts as a calorie restriction mimetic. How do these results align to those observed by indirect calorimetry under calorie restriction? (see for example: https://doi.org/10.1038/s41586-024-08026-3; https://doi.org/10.1016/j.cmet.2023.05.003).

10. Several statements and conclusions regarding the mechanisms and effects proposed to be modulated by semaglutide are based on mRNA expression only (e.g., "GLP-1R activation improves mitochondrial function"). Modulation of gene expression levels by semaglutide is an important result indicative of its mechanism of action, but without functional measures (protein levels, enzymatic activity, among others), it is not possible to make firm conclusions on that regard. For example, in Fig. S7, the increased mRNA levels of Pgc1a, Nrf1, Tfam, Mfn1, Mfn2, ND1, ND4, Cytb, Cytc, Atp6 y Cox1 are indicative of improved mitochondrial functon(in terms of biogenesis, dynamics, respiratory capacity, etc.). However, to firmly state that mitochondrial function is improved it is necessary to confirm these findings with functional measures such as respiratory activity, ATP production, protein levels, etc. The same is valid for Figure S8, where the increased mRNA levels of Nrf2, Cat, Hmox1 and Nqo1 in the semaglutide group is an indicator that the antioxidant response is active and potentially improved, but these transcriptomic measures need to be complemented with protein levels and enzymatic activity (e.g., CAT, HO-1, NQO1, reduction of ROS and markers of oxidative stress).

11. Minor comment: The color of the treatments in Fig. S5e,g is inverted with regards to the rest of the figures of the manuscript.

Discussion section

1. The findings of this study should be discussed more in depth, both in terms of similarities and discrepancies with previous studies on GLP-1 receptor agonists and regarding the authors' hypothesis that semaglutide acts as a calorie restriction mimetic.

2. Do the authors recognize any limitations in their study? Are there any adverse effects of semaglutide?

Methods section

1. In the statistical analysis section, the authors indicate: "Mice were randomized to groups and analysis of mice and tissue samples were performed by investigators blinded to the treatment of the animals. Measurements were taken from distinct samples." Please indicate if treatment groups had homogeneous body weights at randomization, the total number of animals used in the study, including all experiments, and the method to select the animals (if any) for the functional measures performed.

2. Please indicate in the methods section for how long the mice were acclimated to the same conditions after arrival at the facility and before starting the experimental treatments.

3. Please indicate in methods section how mice were housed (group-housed? single-housed?) as this factor greatly affects behavior towards food consumption and thermoregulation, which impact the overall energetic context, a key aspect for this study.

4. Please provide all details describing how subcutaneous injections were performed (and corresponding reference of the protocol), as their short- and long-term effect on local inflammation, tissue damage, and ulceration may not be trivial for several of the biomarkers measured in this study as well as from an ethical standpoint. Did the operator rotate the injection sites throughout the study?

5. What the authors call "Grip Strength Test" in literature is commonly referred to as "cage top test", "inverted screen test", "grid hanging test", "hanging test", "cage wire test" (see: DOI: 10.1126/science.aat3987; 10.1038/bonekey.2015.101; https://doi.org/10.1038/s41596-019-0256-1), among other synonyms. It is strongly recommended to refer to this test with one of these nomenclatures, since the "grip strength test" measures the force (itself) of the limbs obtained with a grip strength meter and, thus, it is not a latency to fall.

6. Most (if not all) statistical analyses in the manuscript were performed through t-tests, which require that the assumptions of normality and homoscedasticity are satisfied. Did the authors test these assumptions for each variable? How? If so, this has not been described in the statistical analysis section and should be provided, indicating by which method this was achieved. Visual inspection (e.g., Fig 1H, Fig. 4e-Sirt5, to name just two) and the nature of some variables (counts, percentages) suggest that the assumptions of this test could have been violated, and for such cases it is strongly recommended to perform estimations using models that are appropriate for the natural distribution of the variables (e.g., Poisson distribution for variables that are counts), tests that allow heterogeneous variances between groups (e.g., Welch's t-test?) or non-parametric tests (e.g., Mann–Whitney U, Wilcoxon signed-rank) as needed. In addition, please provide the exact p-values as well as the coefficients of the statistics used for each test.

Referee #3

(Remarks to the Author)
I co-reviewed this manuscript with one of the reviewers who provided the listed reports.

Version 3:

Reviewer comments:

Referee #1

(Remarks to the Author)
The authors have done a very nice experiment that strengthens the paper. They have responded convincingly to my previous critique.

[redacted]

Referee #2

(Remarks to the Author)
The revised manuscript has been substantially improved, and all concerns raised in the first round of review have been satisfactorily addressed. The authors have conducted additional experiments, clarified several aspects of the study, provided the necessary experimental and analytical details, improved the statistical analyses, and expanded the discussion of their findings.
Critically, the authors have now included an additional pair-feeding experiment (for phenotypic variable assessments, but not the lifespan analysis) consisting of a 24% calorie restriction (CR) group that closely matches the reduction in food intake

observed in semaglutide-treated mice. The degree of restriction appears to have been based on the mean reduction in food intake induced by semaglutide, rather than adjusted over time. If this is indeed the case, this methodological detail be explicitly described in the Methods section. This additional experiment strengthens the study by enabling a direct comparison between semaglutide treatment and caloric restriction, providing a more rigorous framework for evaluating the extent to which semaglutide recapitulates CR effects. The comparison demonstrates that late-life GLP-1R activation reproduces many of the functional benefits associated with CR by attenuating age-related physiological decline and also confers additional benefits across several outcomes, including exploratory behavior, spatial memory, and glucose homeostasis. These findings suggest that some of the effects of semaglutide extend beyond those expected from reduced caloric intake alone. Thus, it should be clearly stated that, while some semaglutide effects are similar to CR, there are other fundamental mechanistic and functional differences that are divergent, which need to be emphasized better, particularly in the discussion in the context of the CR-induced hunger/fasting, likely affecting some of the essential mechanistic underpinnings of CR through the hypothalamus and corticosterone-mediated signaling. Thus, semaglutide is not a true/perfect CR-mimetic.

Importantly, analyses of the CLAMS data, which are now considerably more comprehensive and appropriately interpreted, show that, despite producing an equivalent reduction in caloric intake, CR and semaglutide elicit distinct metabolic adaptations. Specifically, semaglutide preserves daytime metabolic rate, whereas pair-fed CR reduces daytime oxygen consumption, carbon dioxide production, and energy expenditure. These findings indicate that CR is accompanied by hunger-driven behavioral and metabolic adaptations, whereas semaglutide suppresses food intake without eliciting the same compensatory feeding-associated metabolic rhythms. This represents an important and interesting contribution to the field that should be highlighted in the manuscript.

The authors have also incorporated additional baseline and longitudinal assessments of several physiological outcomes, further supporting their conclusion that semaglutide delays the onset of age-related functional decline. Moreover, they complement the transcriptional analyses with functional readouts that strengthen the biological interpretation of candidate mechanisms modulated by semaglutide treatment. These new experiments are accompanied by an expanded discussion of the results.

Finally, the revised title is an improvement over the original. However, it would be more accurate to explicitly state that the findings were obtained in female mice, given the well-established sex differences in many of the physiological outcomes examined in this study. Therefore, it would be important to revise the title to: "GLP-1 Receptor Activation Late in Life Slows Aging and Extends Lifespan in Female Mice".

Referee #3

(Remarks to the Author)
I co-reviewed this manuscript with one of the reviewers who provided the listed reports.

Open Access This Peer Review File is licensed under a Creative Commons Attribution 4.0 International License, which permits use, sharing, adaptation, distribution and reproduction in any medium or format, as long as you give appropriate credit to the original author(s) and the source, provide a link to the Creative Commons license, and indicate if changes were made.
In cases where reviewers are anonymous, credit should be given to 'Anonymous Referee' and the source.

The images or other third party material in this Peer Review File are included in the article's Creative Commons license, unless indicated otherwise in a credit line to the material. If material is not included in the article's Creative Commons license and your intended use is not permitted by statutory regulation or exceeds the permitted use, you will need to obtain permission directly from the copyright holder.
To view a copy of this license, visit https://creativecommons.org/licenses/by/4.0/

18th September 2025

Dear Professor Chen, dear Danica

Your manuscript, "GLP-1 Receptor Activation Slows Aging and Extends Lifespan", has now been seen by 2 referees (plus a co-reviewer), whose comments are attached below. I regret that, in the light of their advice, we have decided that we cannot offer to publish your manuscript in Nature.

While the referees find your work of interest, they raise concerns, which cast doubt on the strength of the novel conclusions that can be drawn from the study. We feel that these reservations are sufficiently important as to preclude publication of the study in Nature.

I am sorry that we cannot be more positive on this occasion. We hope that you will find our referees' comments helpful when preparing your paper for submission elsewhere.

Yours sincerely

[redacted]
Senior Editor, Nature

Referees' comments:

Referee #1 (Remarks to the Author):

This paper describes studies of a GLP-1 receptor agonist, semaglutide, on parameters of aging in mice. The underlying hypothesis driving this work is that effects of semaglutide to reduce feeding mimics the effects of dietary caloric restriction that has been shown to promote longevity. The principal findings reported are that: 1) semaglutide administration to 20 week old mice extended lifespan by ~ 14 % compared to control treated mice; 2) semaglutide mice performed significantly better in tests of aging such as locomotor activity, muscle strength, and cognition; 3) semaglutide improved glucose tolerance and respiratory capacity; 4) semaglutide had salutary effects on established cellular and tissue markers of aging including stem cell dynamics, proteostasis, inflammation and mitochondrial function. The authors conclude from these results that semaglutide phenocopies the effects of caloric restriction to extend the lifespan and healthspan of mice.

This is an interesting set of observations on a timely and potentially impactful topic. GLP-1-based drugs are available and more refined versions with greater potency and ease of use are in development. These compounds are highly effective for treating diabetes, mitigate several co-morbidities of obesity such as NAFLD and obstructive

sleep apnea, and are now the most potent weight loss drugs on the market. The demonstration that chronic treatment with semaglutide extends lifespan and quality adds a novel and important therapeutic application. The data presented in this paper make this demonstration in a convincing manner. The experiments are straightforward, and most have been used in previous studies of aging and caloric restriction. The sample sizes are adequate and the key effect sizes (e.g. longevity, weight loss) are meaningful. This is a paper that will have broad interest.

Response: We thank the reviewer for their interest in our study and for describing it as “timely and potentially impactful”, noting it “adds a novel and important therapeutic application”, and that we “make this demonstration in a convincing manner”.

The short-coming of this research is that these outcomes are mostly predictable. It is well known that semaglutide reduces food intake and body weight in rodents, humans and non-human primates. Thus, it is not surprising that medical caloric restriction has many features of dietary caloric restriction. In fact, it would have been surprising if semaglutide did not share many hallmarks of chronic food restriction. The study is essentially descriptive, although the phenomenon in question is interesting. It seems likely that many of the outcome measures here are a function of negative energy balance, although it is possible that some were mediated directly at the GLP-1 receptor in specific tissues; this question is not explored. Different approaches could have added depth to the results by trying to identify differences between semaglutide and caloric restriction, e.g. a matched calory restriction comparator, or even the use of doses of semaglutide that do not cause substantial weight loss.

Response: We thank the reviewer for their thoughtful comment. While the pro-longevity effects of dietary calorie restriction are well established, the benefits are not universal. The effects of calorie restriction are influenced by individual characteristics, including factors such as age and health condition (Francesco, Nature 2024; Mitchell, Cell Metabolism 2016; Ingram & de Cabo, Ageing Res Rev 2017; Forster, FASEB J 2003; Lipman, Aging Clin Exp Res 1995).Therefore, the long-term consequences of GLP-1R activation cannot be assumed, especially when treatment is initiated late in life. Our study directly addresses this outstanding question. We have included this clarification in the introduction.

We thank the reviewer’s suggestion to compare directly GLP-1R activation and matched calorie restriction. We performed a longitudinal study in 20-month-old mice that were treated with vehicle, semaglutide, or matched (24%) calorie restriction for 5 months, and assessed at baseline and after 2 and 4 months of treatment. Both semaglutide treatment and calorie restriction led to comparable body weight and fat loss (Data below A-C) (Figure S10A-C).

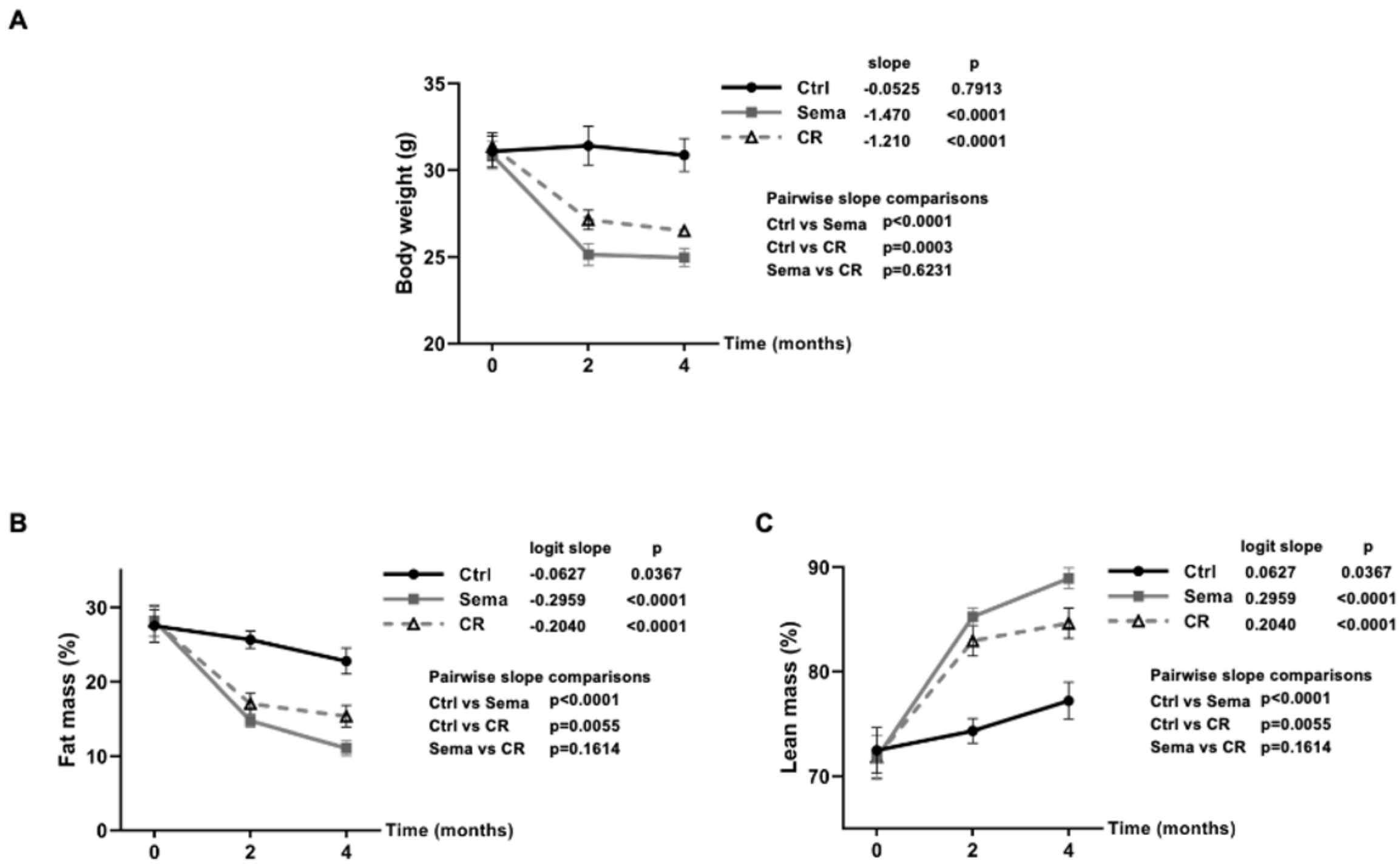

Comparison of 20-month-old female C57BL/6 mice treated with vehicle, semaglutide, or calorie restriction for 5 months. Mice were assessed at the baseline or after 2 or 4 months of treatment. n=10.
A, Body weight.
B, C, The percentage of fat mass (B) and lean mass (C) in the Echo MRI test.

Control mice exhibited progressive declines in locomotor activity and exploratory behavior in novel environments, motor coordination, muscle function, glucose control, and spatial memory over the course of the longitudinal study (Data below A-I)(Figure 5). Semaglutide treated mice showed significantly different trajectories from control mice across measures (Data below A-I)(Figure 5). Semaglutide treatment preserved baseline function and produced trajectories comparable to those of calorie restricted mice for total locomotor activity in the open field (Data below A)(Figure 5A) and elevated plus maze tests (Data below C)(Figure 5C), rotarod performance (Data below F)(Figure 5F), inverted screen performance (Data below G)(Figure 5G), and treadmill endurance (Data below H)(Figure 5H). Notably, semaglutide treatment led to improvements above baseline, and to more favorable trajectories than calorie restriction, in open field center time (Data below B)(Figure 5B), Barnes maze performance (Data below E)(Figure 5E), and glucose tolerance (Data below I)(Figure 5I). These findings indicate that GLP-1R activation late in life recapitulates many functional benefits of calorie restriction by attenuating age-associated decline, while conferring additional benefits in exploratory drive, spatial memory, and glucose control, suggesting effects beyond reduced calorie intake alone.

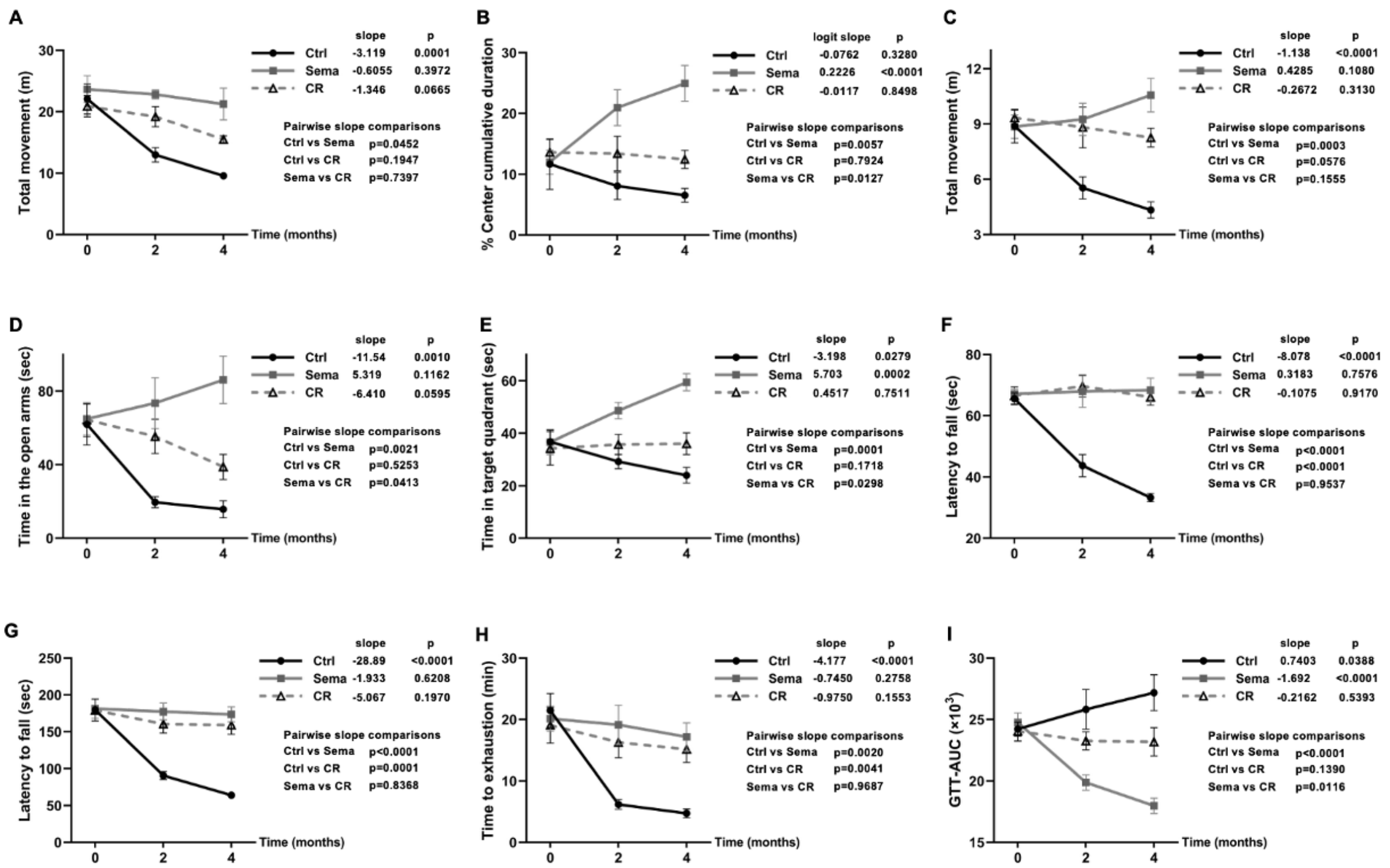

Comparison of 20-month-old female C57BL/6 mice treated with vehicle, semaglutide, or calorie restriction for 5 months. Mice were assessed at the baseline or after 2 or 4 months of treatment. n=10.
A, B, Total movement (A) and the percentage of center cumulative duration (B) in the open field test.
C, D, Total movement (C) and time in the open arms (D) in the elevated plus maze test.
E, Time in target quadrant in the Barnes maze test.
F, Latency to fall in the rotarod test.
G, Latency to fall in the inverted screen test.
H, Time to exhaustion in the treadmill exhaustion test.
I, The glucose tolerance test.

As a minor point it was difficult to find a description of the semaglutide dose and treatment schedule in the text.

Response: We have included this information in “Methods” under “Mice”: “Mice received daily subcutaneous injection of 10 nmol/kg semaglutide or an equal volume of saline”.

[redacted]

Referee #2 (Remarks to the Author):

This study shows that daily subcutaneous injection of semaglutide (10 nmol/kg) in 20-month-old C57BL/6J female mice over three months modulates a range of core molecular, cellular, and functional metabolic biomarkers, indicating its potential to alleviate various hallmarks of aging. However, this requires further validation because many conclusions are based solely on mRNA expression levels, and functional products

are not always measured. By continuing semaglutide injections until the end of the experimental period (about 28 months of age, which is the age at which the last control saline-injected animal died—quite young for this sex and strain), the authors demonstrate that this GLP-1 receptor agonist may extend median lifespan by approximately 13% in this sex and strain.

This work builds on earlier findings that show similar beneficial effects for semaglutide or other GLP-1 receptor activators, potentially indicating that this compound can also extend lifespan under “normal aging,” unlike previous studies that demonstrated this effect in mouse models of disease.
Overall, the manuscript is written clearly and is well-organized. The experiments designed, techniques used, and general methodology are solid. However, several aspects need revision, clarification, or expansion, both in terms of experimental or analytical validation and discussion enrichment, to effectively test the author’s hypothesis and support their main conclusion that semaglutide can serve as a calorie restriction mimetic, slowing aging and extending lifespan. These issues are outlined below.

Response: We thank the reviewer for pointing out the novel findings of our study under “normal aging” and that “the experiments designed, techniques used, and general methodology are solid”. We appreciate the constructive suggestions that have significantly improved our study.

General comments

1. It is recommended to define all abbreviations used throughout the manuscript at their first mention, regardless of how common they might be in the literature.

Response: We have defined abbreviations, as recommended.

2. It is recommended to indicate the sex and strain of the mice used in each and all figure legends.

Response: We have included sex and strain of the mice in all figure legends, as recommended.

Title section

1. The phrase “Slows Aging” may be too broad for this article because no longitudinal or baseline measures (before starting experimental treatments) have been taken for any variables except body weight. Additionally, healthspan cannot be assessed through endpoint analysis since it doesn't allow determination of when impairments begin. Furthermore, only “old” C57BL/6J female mice were used in this study, so the results

cannot be generalized to other sexes, strains, or age groups. Therefore, the title should be rephrased to more accurately reflect what was actually studied.

Response: We thank the reviewer for raising these important points. We agree that claims regarding “slowing aging” should be supported by longitudinal measurements rather than endpoint analyses alone. To address this, we performed a longitudinal study in 20-month-old female C57BL/6 mice that were treated with saline, semaglutide, or matched (24%) calorie restriction for 5 months, and assessed at baseline and 2 and 4 months after intervention. No differences were observed among groups at baseline (Data below)(Figure S7).

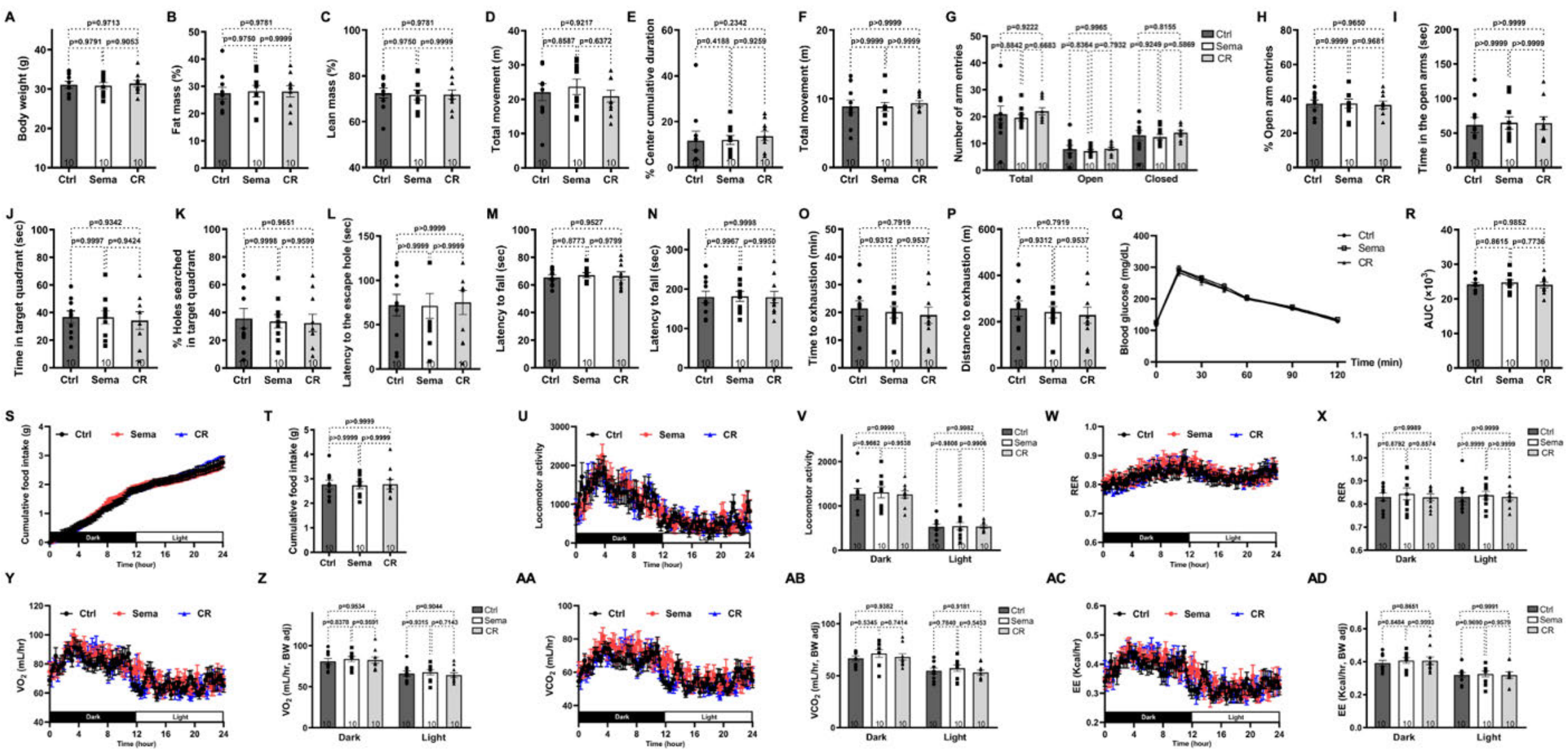

A longitudinal study of 20-month-old female C57BL/6 mice treated with vehicle, semaglutide, or calorie restriction for 5 months. Data shown are baseline physiological measures. n=10.
A, Body weight.
B, C, The percentage of fat mass (B) and lean mass (C) in the Echo MRI test.
D, E, Total movement (D) and the percentage of center cumulative duration (E) in the open field test.
F-I, Total movement (F), number of arm entries (G), the percentage of open arm entries (H), time in the open arms (I) in the elevated plus maze test.
J-L, Time in target quadrant (J), percentage of holes searched in target quadrant (K), latency to the escape hole (L) in the Barnes maze test.
M, Latency to fall in the rotarod test.
N, Latency to fall in the inverted screen test.
O, P, Time (O) and distance to exhaustion (P) in the treadmill exhaustion test.
Q, R, Blood glucose (Q) and the area under the curve (AUC) (R) in the glucose tolerance test.
S-AD, Measurements in metabolic cage.
S, T, Cumulative food intake (S) and summarized (T).
U, V, Locomotor activity (U) and summarized by light and dark cycles (V).
W, X, Respiratory exchange ratio (W) and summarized by light and dark cycles (X).
Y, Z, Oxygen consumption absolute value (Y) and summarized by light and dark cycles after adjustment using ANCOVA with body weight as a covariate (Z).
AA, AB, Carbon dioxide production absolute value (AA) and summarized by light and dark cycles after adjustment using ANCOVA with body weight as a covariate (AB).
AC, AD, Energy expenditure absolute value (AC) and summarized by light and dark cycles after adjustment using ANCOVA with body weight as a covariate (AD).

Control mice exhibited progressive declines in locomotor activity and exploratory behavior in novel environments, motor coordination, muscle function, glucose control, and spatial memory (Data below A-I)(Figure 5). Semaglutide treated mice showed significantly different longitudinal trajectories from controls across measures, with some outcomes maintained at baseline levels and others significantly improved above baseline (Data below A-I)(Figure 5). These findings indicate that late life GLP-1R activation attenuates age-associated physiological decline.

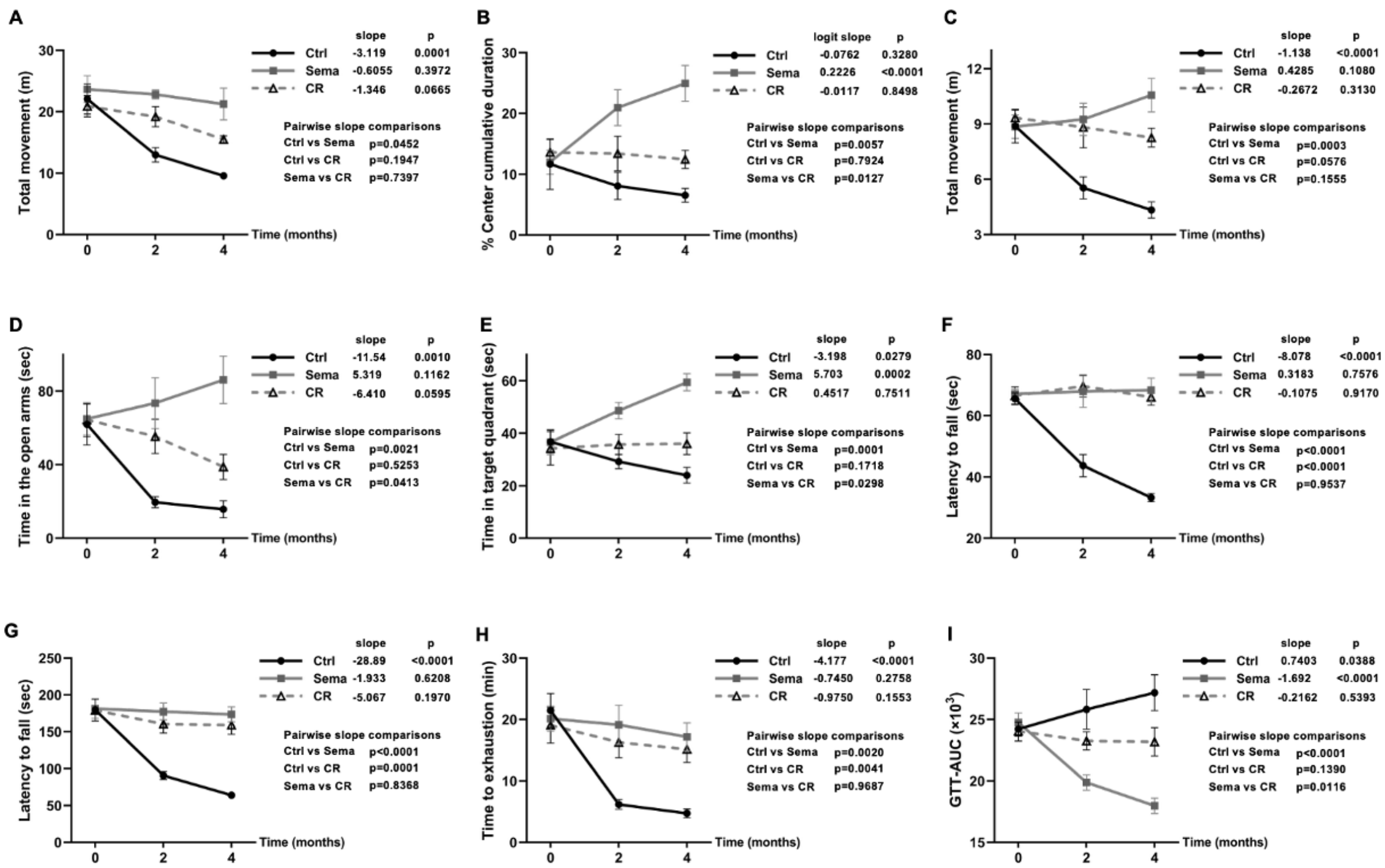

Comparison of 20-month-old female C57BL/6 mice treated with vehicle, semaglutide, or calorie restriction for 5 months. Mice were assessed at the baseline or after 2 or 4 months of treatment. n=10.
A, B, Total movement (A) and the percentage of center cumulative duration (B) in the open field test.
C, D, Total movement (C) and time in the open arms (D) in the elevated plus maze test.
E, Time in target quadrant in the Barnes maze test.
F, Latency to fall in the rotarod test.
G, Latency to fall in the inverted screen test.
H, Time to exhaustion in the treadmill exhaustion test.
I, The glucose tolerance test.

All experiments were conducted in aged (20 months old) female C57BL/6 mice. The study was designed to test whether GLP-1R activation, a putative calorie restriction mimetic, can modify physiological decline and lifespan when initiated at advanced age. Because the effects of calorie restriction are influenced by individual characteristics, including factors such as age and health condition (Francesco, Nature 2024; Mitchell, Cell Metabolism 2016; Ingram & de Cabo, Ageing Res Rev 2017; Forster, FASEB J 2003; Lipman, Aging Clin Exp Res 1995), it is critical to determine how long-term use of GLP-1 medicines influences the aging process and lifespan, particularly when treatment is initiated late in life. Our study directly addresses this question. We have revised the title to reflect the scope of the study, emphasizing late-life intervention in mice.

Female mice were selected to minimize confounding effects of male aggression and injury, consistent with prior long-term aging studies (Francesco, Nature 2024). The Abstract, Methods, Figure Legends, and Discussion now clearly specify strain, sex, and age to ensure appropriate interpretation of the findings, and the title focuses on the biological question.

Abstract section

1. It is common practice to exclude references in the abstract of original articles, as their focus should be on what was done, the main results, and the conclusions, rather than on citing other work. I recommend removing references 1-11 from the abstract.

Response: Indeed, most journals require abstracts without references. However, Nature formatting guideline (https://www.nature.com/nature/for-authors/formatting-guide) states "articles start with a fully referenced summary paragraph, ideally of no more than 200 words, which is separate from the main text and avoids numbers, abbreviations, acronyms or measurements unless essential".

2. Please indicate the sex, strain, age, and number of mice used in this study, as well as the duration of treatment with semaglutide.

Response: We have included this information in the abstract. "Here, treatment of 20-month-old female C57BL/6 mice with semaglutide, a GLP-1R agonist, for three months improved physiological function, attenuated hallmarks of aging, and modulated nutrient sensors and conserved genetic regulators of aging. Continued treatment extended lifespan."

Introduction section

1. In the third sentence, the authors state: "Calorie restriction engages several evolutionarily conserved signaling pathways, such as sirtuins and insulin/IGF-1 signaling, to elicit a beneficial response." Please review the spelling of the word "illicit" (Did you mean "elicit"?).

Response: We thank the reviewer for pointing out the typo. We have corrected it.

Results section

1. At the beginning of the results section, the authors state: "We treated old (20 months old) mice daily with or without semaglutide, a GLP-1R agonist." Please specify what "treated" means (such as subcutaneous injection), as well as the sex and strain of the mice, and how long the animals received the semaglutide injections.

Response: We have added this information in the Results section. "We treated 20-month-old female C57BL/6 mice daily with either saline or semaglutide, a GLP-1R agonist, via subcutaneous injection. For lifespan study, treatment continued for the duration of the lifespan. For physiological, molecular, and cellular studies, a separate cohort was treated for three months."

2. Fig. 1a: The median and maximum lifespan of control saline-injected animals is quite short for this sex and strain (https://doi.org/10.1007/s11357-024-01226-9; https://doi.org/10.1016/j.cmet.2021.08.013). Could the daily injections for

approximately 260 days influence their normal lifespan? Alternatively, might this result be due to other factors, such as genetic or environmental conditions related to the facility where the experiment was conducted? Regardless, the lifespan results should be discussed in the context of existing literature. Ideally, the lifespan of the control saline-injected group should be compared with that of an intact control group kept under the same conditions but without saline injections. Additionally, the methods section describes conditions that classify an animal as severely moribund, justifying euthanasia. Please specify (1) how many animals experienced each of these conditions, (2) how these causes of death were distributed among the experimental groups (were they evenly distributed?), and (3) the ages at which these causes of death occurred. If animals that died from these reasons were included in the lifespan analysis, clarify whether they were censored or not. If censored, explain how this is represented in the Kaplan-Meier curves and accounted for in survival estimates. Also, state the statistical test used for survival analysis.

Response: We thank the reviewer for their insightful comment. Direct comparison from the National Institute on Aging (NIA) Interventions Testing Program (ITP) shows that lifespans differ substantially among the three ITP test sites (Harrison, Aging Cell 2013). As an example, site-specific medians for male control UM-HET3 mice were 704 days (UT), 807 days (TJL/JAX), and 924 days (UM), a difference of 220 days between UT and UM. This paper explicitly discusses that site-specific factors cause these large inter-site differences.

In the literature, median lifespans for C57BL/6 mice also vary widely: 550 days (Conti, Science 2006), 713 days (Baker, Nature 2016), 741 days (Latorre-Pellicer, Nature 2016), 750 days (Qu, Nature 2025), 760 days (Zhang, Nature 2023), 785 days (Mitchell, Cell Metabolism 2016). In the studies cited by the reviewer, medians were 805 days (Palliyaguru, Cell Metabolism 2021) and 867 days (Luciano, Geroscience 2024). Our observed median lifespan (742 days) falls comfortably within this reported range. We have included this discussion in the manuscript.

The distribution of endpoint categories was consistent with the literature, with high incidents of rectal prolapse for aging female C57BL/6 mice (Pettan-Brewer, Pathobiology of aging and age-related diseases 2011; Ogiso, Experimental Animals 2025), and did not differ significantly between groups (p=0.9431)(Data below A)(Figure S3A). Age at death appeared to be delayed by semaglutide across several categories unrelated to tumor (Data below B-G)(Figure S3B-G), consistent with a broader delay in age-associated physiological deterioration.

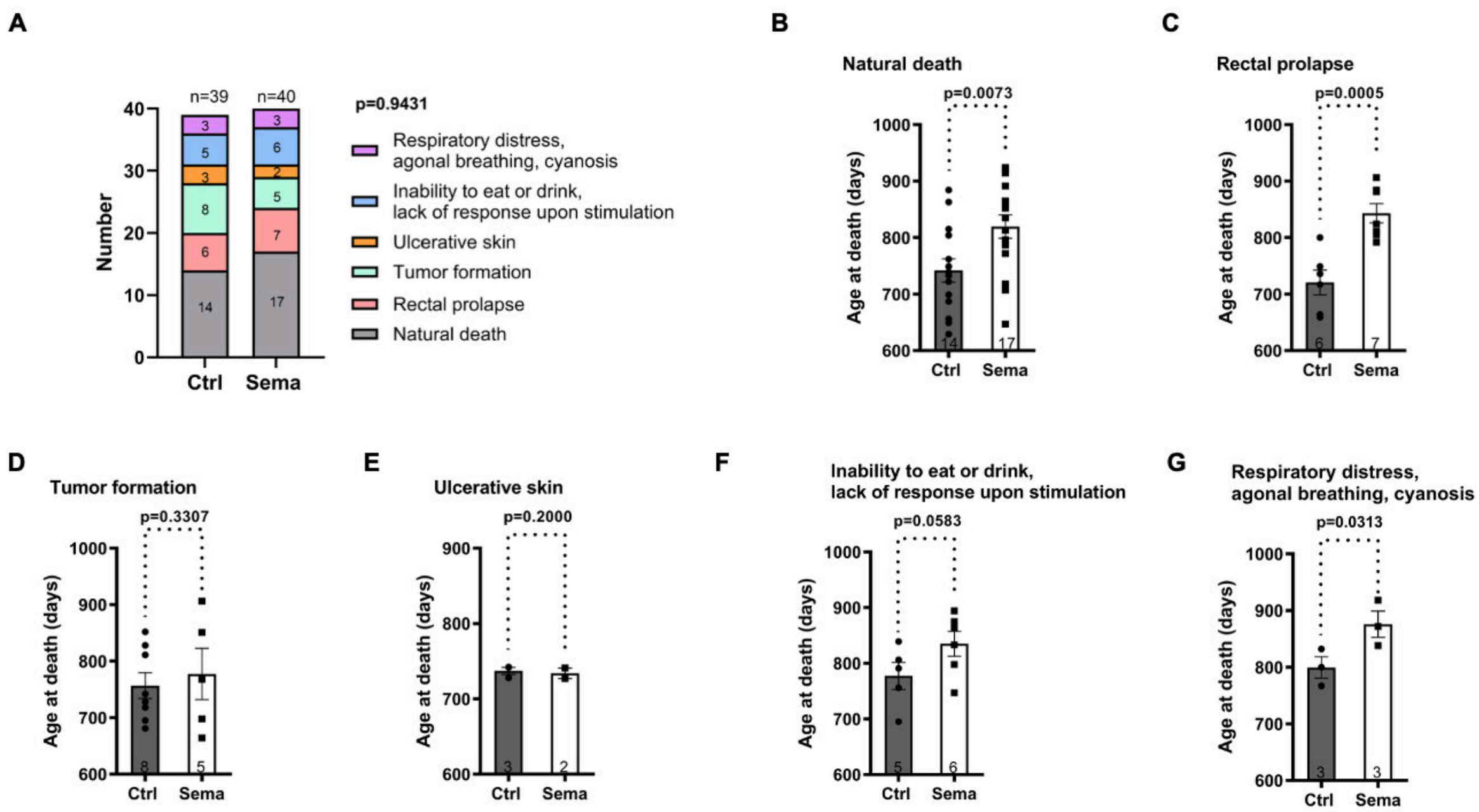

20-month-old female C57BL/6 mice were treated with vehicle or semaglutide.
A, Distribution of endpoint categories in the lifespan study. n=39,40.
B-G, Age at death for the indicated endpoint categories in the lifespan study. n=14, 17 (B), 6, 7 (C), 8, 5 (D), 3, 2 (E), 5, 6 (F), 3 (G).

We followed established practices for lifespan study. “Both mice euthanized or found dead were represented as deaths in the survival curves. Mice euthanized due to injuries unrelated to imminent death were treated as censored” (Francesco, Nature 2024). In our study, no mice were removed from the study for reasons unrelated to age-associated decline and censored in the survival analysis. Statistical significance was determined using the log-rank test. We have included this information in the Methods and Table S3 for statistical analyses.

3. Subsection “GLP-1R activation extends healthspan” and Fig. 1. As noted above, healthspan cannot be assessed through endpoint analysis and requires longitudinal data; otherwise, it is impossible to determine when the onset of impairments occurs. In addition, a major general limitation of the experimental design of this study is that baseline measures have not been performed. This precludes one from distinguishing whether the treatment with semaglutide actually delayed the onset of the impairments observed in the control animals and/or whether the animals in the semaglutide group were different from the control ones before initiating the treatment. At most, with this experimental design, the authors can state that “X” variable was improved by the treatment with semaglutide for 3 months (and acknowledging the limitation that they do not have baseline measurements). Therefore, both the title of the subsection “GLP-1R activation extends healthspan” and the last sentence of that subsection in which the authors state “Together, these data suggest that semaglutide treatment extends healthspan“ Statement “semaglutide treatment extends healthspan”, should be revised to accurately reflect their results.

Response: We thank the reviewer for raising these important points. We agree that it is important to perform longitudinal measurements rather than endpoint analyses alone. It is also critical to include baseline measurements. To address this, we performed a longitudinal study in 20-month-old female C57BL/6 mice that were treated with saline, semaglutide, or matched (24%) calorie restriction for 5 months, and assessed at baseline and after 2 and 4 months of intervention. No differences were observed among groups at baseline (Data below)(Figure S7).

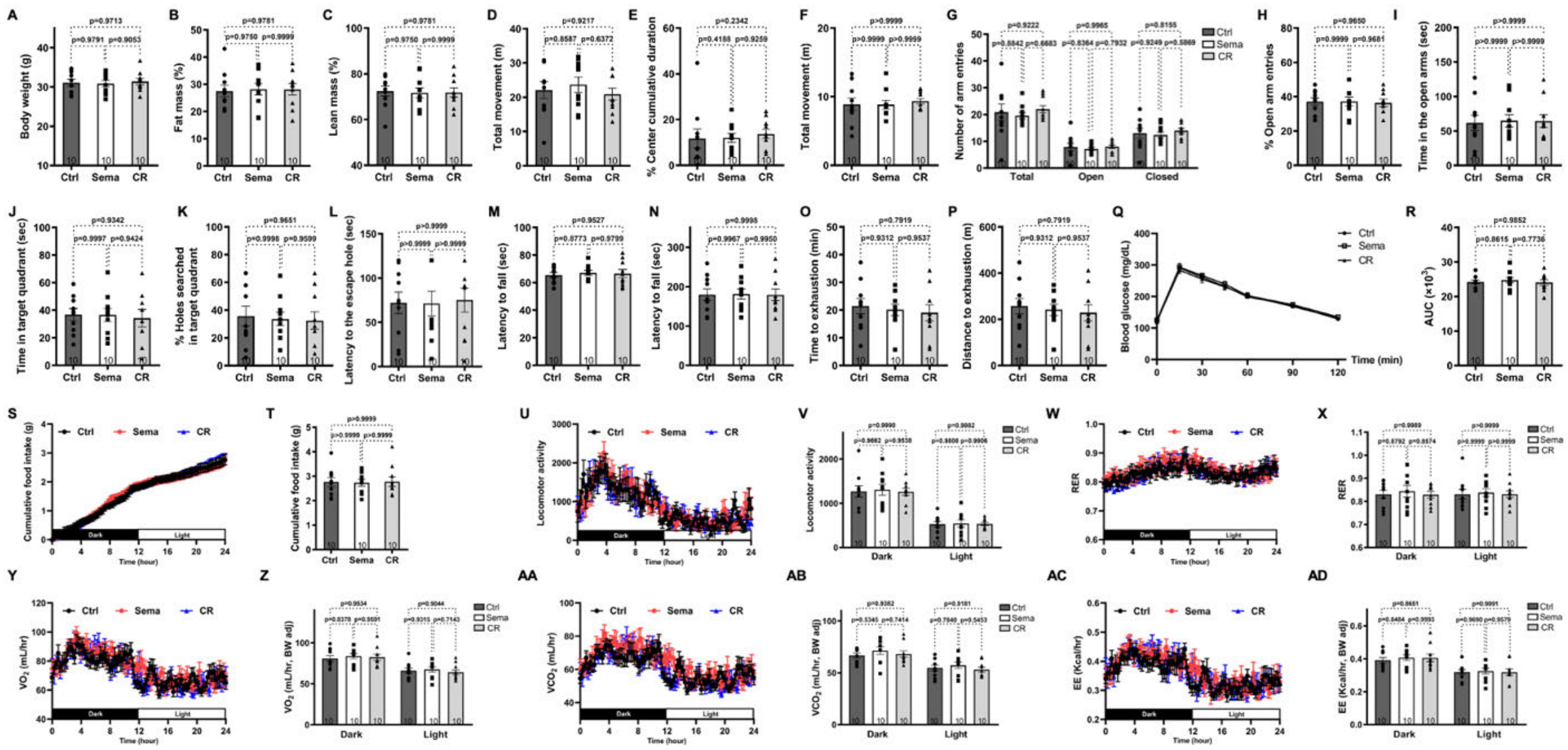

A longitudinal study of 20-month-old female C57BL/6 mice treated with vehicle, semaglutide, or calorie restriction for 5 months. Data shown are baseline physiological measures. n=10.
A, Body weight.
B, C, The percentage of fat mass (B) and lean mass (C) in the Echo MRI test.
D, E, Total movement (D) and the percentage of center cumulative duration (E) in the open field test.
F-I, Total movement (F), number of arm entries (G), the percentage of open arm entries (H), time in the open arms (I) in the elevated plus maze test.
J-L, Time in target quadrant (J), percentage of holes searched in target quadrant (K), latency to the escape hole (L) in the Barnes maze test.
M, Latency to fall in the rotarod test.
N, Latency to fall in the inverted screen test.
O, P, Time (O) and distance to exhaustion (P) in the treadmill exhaustion test.
Q, R, Blood glucose (Q) and the area under the curve (AUC) (R) in the glucose tolerance test.
S-AD, Measurements in metabolic cage.
S, T, Cumulative food intake (S) and summarized (T).
U, V, Locomotor activity (U) and summarized by light and dark cycles (V).
W, X, Respiratory exchange ratio (W) and summarized by light and dark cycles (X).
Y, Z, Oxygen consumption absolute value (Y) and summarized by light and dark cycles after adjustment using ANCOVA with body weight as a covariate (Z).
AA, AB, Carbon dioxide production absolute value (AA) and summarized by light and dark cycles after adjustment using ANCOVA with body weight as a covariate (AB).
AC, AD, Energy expenditure absolute value (AC) and summarized by light and dark cycles after adjustment using ANCOVA with body weight as a covariate (AD).

Control mice exhibited progressive declines in locomotor activity and exploratory behavior in novel environments, motor coordination, muscle function, glucose control, and spatial memory (Data below A-I)(Figure 5). Semaglutide treated mice showed significantly different longitudinal trajectories from controls across measures, with some outcomes maintained at baseline levels and others significantly improved above baseline (Data below A-I)(Figure 5). These findings indicate that late life GLP-1R activation attenuates age-associated physiological decline.

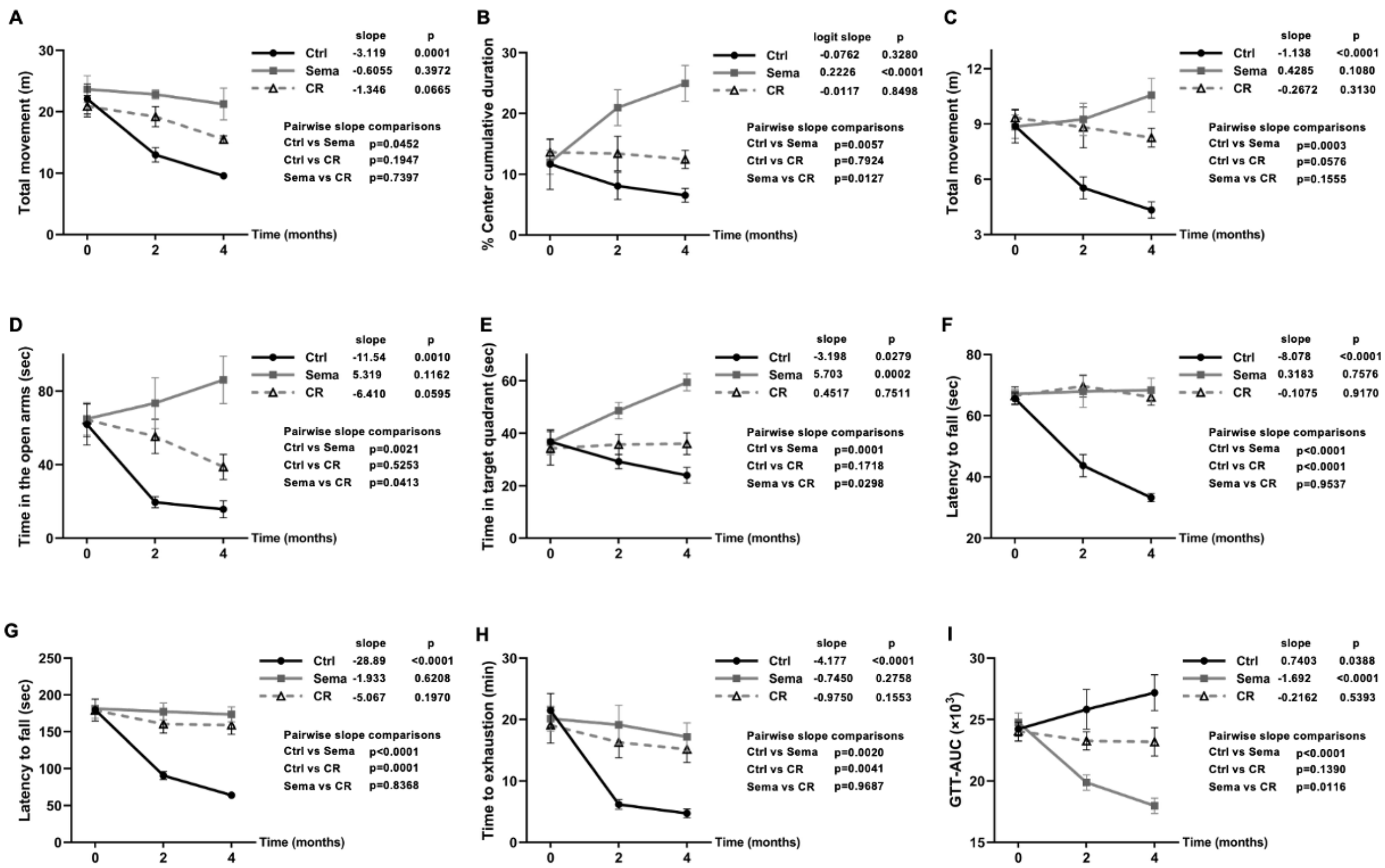

Comparison of 20-month-old female C57BL/6 mice treated with vehicle, semaglutide, or calorie restriction for 5 months. Mice were assessed at the baseline or after 2 or 4 months of treatment. n=10.
A, B, Total movement (A) and the percentage of center cumulative duration (B) in the open field test.
C, D, Total movement (C) and time in the open arms (D) in the elevated plus maze test.
E, Time in target quadrant in the Barnes maze test.
F, Latency to fall in the rotarod test.
G, Latency to fall in the inverted screen test.
H, Time to exhaustion in the treadmill exhaustion test.
I, The glucose tolerance test.

4. Regarding the results of the behavioral test presented in Fig. 1. Could the increases in locomotor and exploratory behaviors in the semaglutide-treated mice be related with foraging behavior?

Response: We appreciate the reviewer’s thoughtful question. If the increased locomotor/exploratory behaviors reflected foraging driven by hunger, we would expect it to be most evident in calorie restricted mice and to track with food anticipation. Consistent with this, calorie restricted mice typically consumed their daily food allotment soon after it was provided and then underwent a prolonged fasting period (Data below A)(Figure S8S) and showed increased activity later in the light cycle when anticipating the next feeding (Data below B)(Figure S8U), which is consistent with foraging behavior. In contrast, semaglutide-treated mice had suppressed appetite and consumed their daily intake more gradually across the day (Data below A)(Figure S8S), and they did not show the same end-of-day activity increase associated with food anticipation (Data below B)(Figure S8U). Thus, the activity pattern in semaglutide-treated mice is not consistent with a hunger-driven foraging explanation.

There is a distinction between general home-cage/metabolic-cage activity and behavior in novelty-based assays. While overall locomotor activity in the metabolic cage was comparable between groups (Data below C)(Figure S8V), both calorie restricted and semaglutide-treated mice showed increased activity in the open field and elevated plus maze (10–15 min tests) (Data below D, E)(Figure S8D, F), which primarily assess responsiveness to a novel environment and exploration-related behavior rather than feeding anticipation. We have added this explanation to the manuscript.

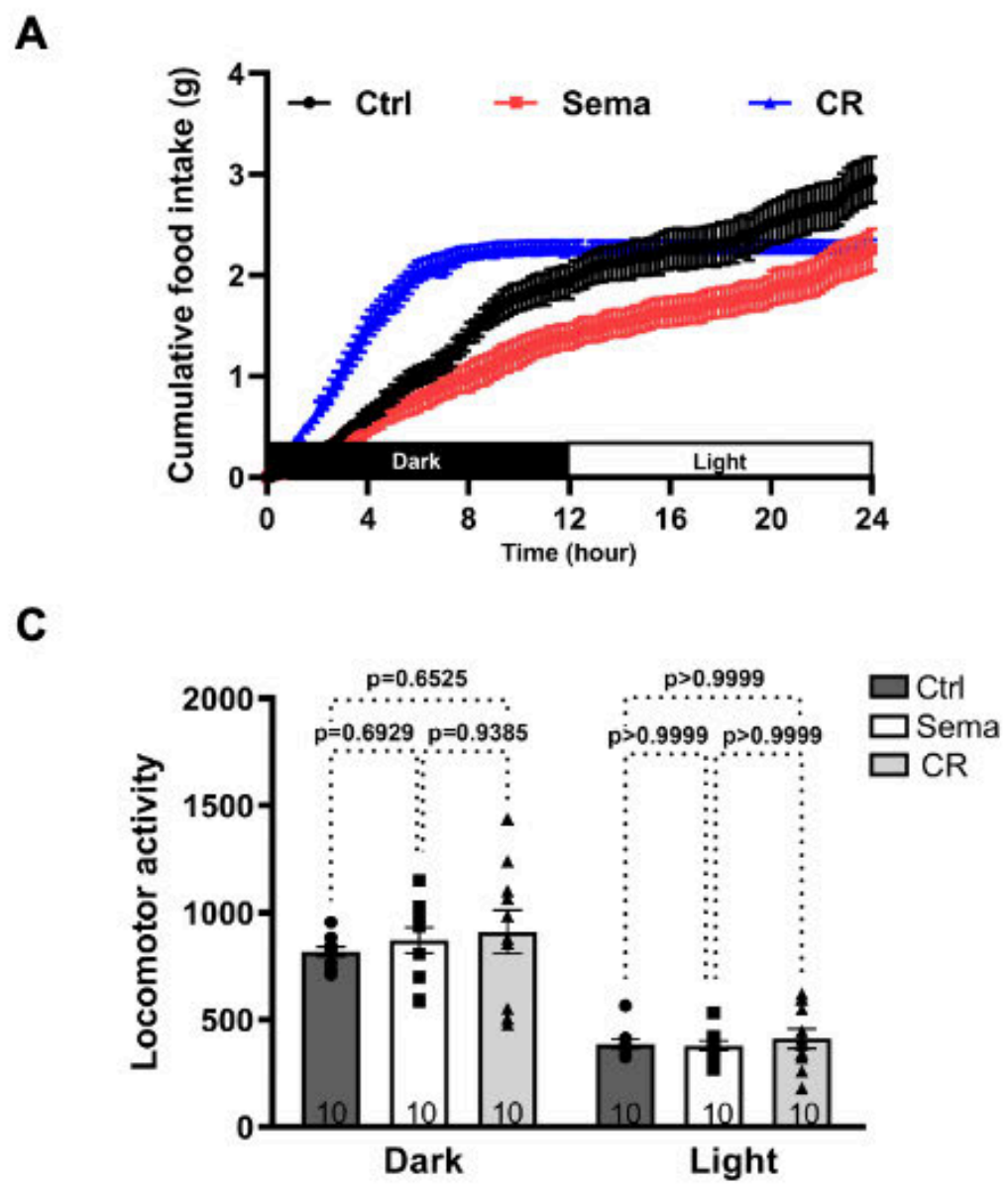

A longitudinal study of 20-month-old female C57BL/6 mice treated with vehicle, semaglutide, or calorie restriction for 5 months. Data shown are physiological measures after 2 months of treatment.
A-C, Measurements in metabolic cage. n=10.
A, Cumulative food intake.
B, C, Locomotor activity (B) and summarized by light and dark cycles (C).
D, Total movement in the open field test. n=10.
E, Total movement in the elevated plus maze test. n=10.

5. Fig. 1: Were the same 10 animals measured in the tests shown in Fig. 1b-q? Was this a separate group of animals or were they a subgroup of the animals used in the lifespan analysis? How were the animals selected if they were a subgroup of the animals used in the lifespan analysis? What was the total number of animals used in this study, including all tests performed and sample collection? Please describe this information in detail in methods or/and results section.

Response: The same 10 mice were used for all tests in Fig. 1b-q. This is a separate group of mice from those in the lifespan study. For the lifespan study, 39 mice received saline and 40 mice received semaglutide for the duration of the lifespan. For physiological, molecular, and cellular studies, a separate cohort was treated for 3 months. 10 pairs for physiological assessments, 5 pairs for neural stem cell studies requiring tissue fixation, and 6 pairs for other molecular and cellular analyses. The longitudinal study included a separate cohort of mice (10 mice per group) treated with

saline, semaglutide, or calorie restriction for 5 months. We have included this information in the Methods and the Results.

6. Fig. S1a: The authors show that after ~10 days on treatment, a reduction in body weight is observed in the semaglutide-treated animals compared to the control group. Was food consumption inhibited or reduced by the treatment with semaglutide? Please provide the food consumption trajectories in each experimental treatment, as this information is critical to understand the extent to which the reduction in body weight is explained by reduced feed intake or other mechanisms and, thus, assess the feasibility of the authors' hypothesis regarding that semaglutide can act as a calorie restriction mimetic. Ideally a pair-fed group or weight matched group should be involved to ascertain whether the effect is a drug effect or "simply" a caloric restriction effect.

Response: We thank the reviewer for raising these important points. Food intake data is including below and in Figure S1A, B, showing semaglutide reduced food intake by 24%.

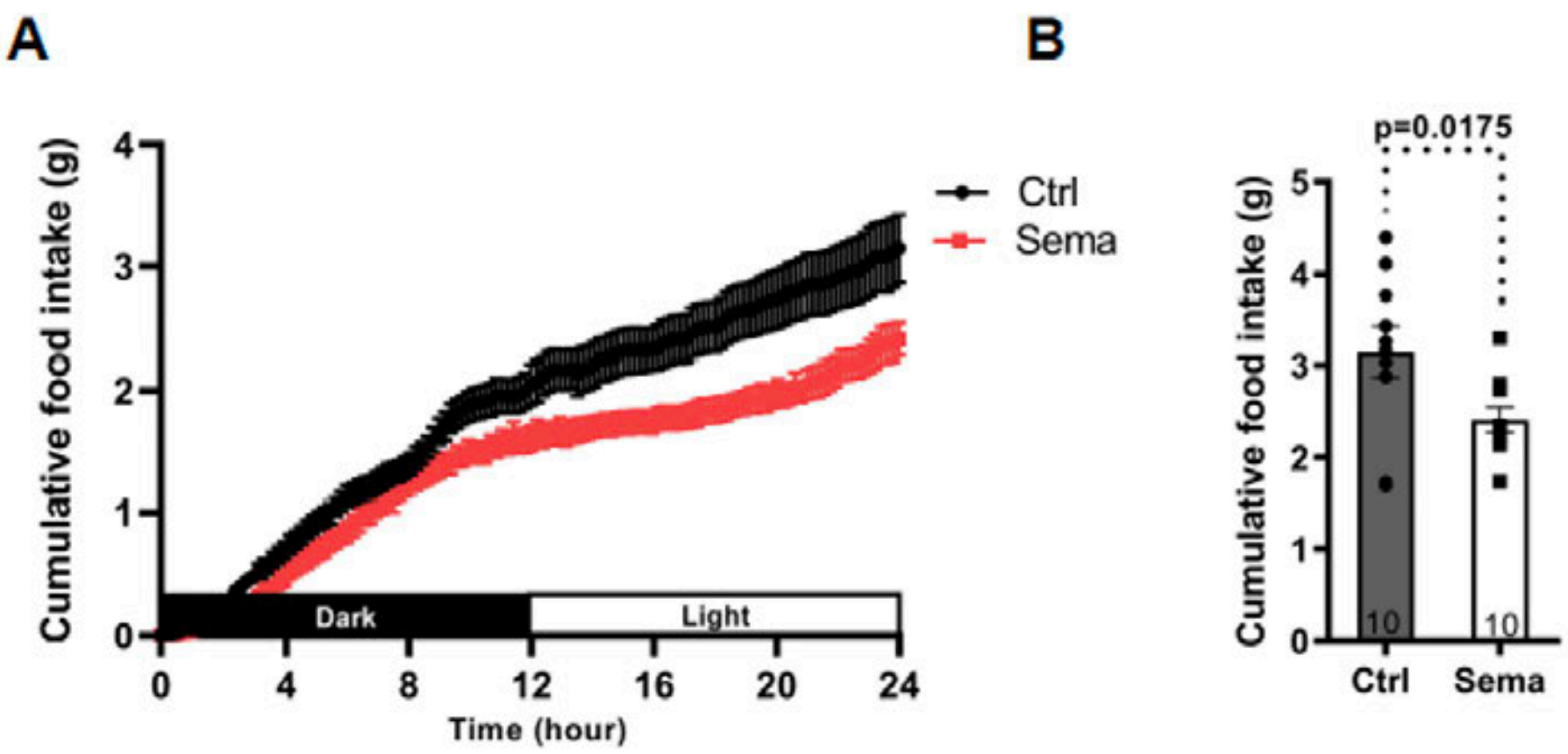

Comparison of 20-month-old female C57BL/6 mice treated with vehicle or semaglutide for 3 months in metabolic cage. Cumulative food intake (A) and summarized (B). n=10.

We agree that it is important to include a pair-fed group. To address this, we performed a longitudinal study in 20-month-old female C57BL/6 mice that were treated with saline, semaglutide, or matched (24%) calorie restriction for 5 months, and assessed at baseline and after 2 and 4 months of intervention. No differences were observed among groups at baseline (Data below)(Figure S7).

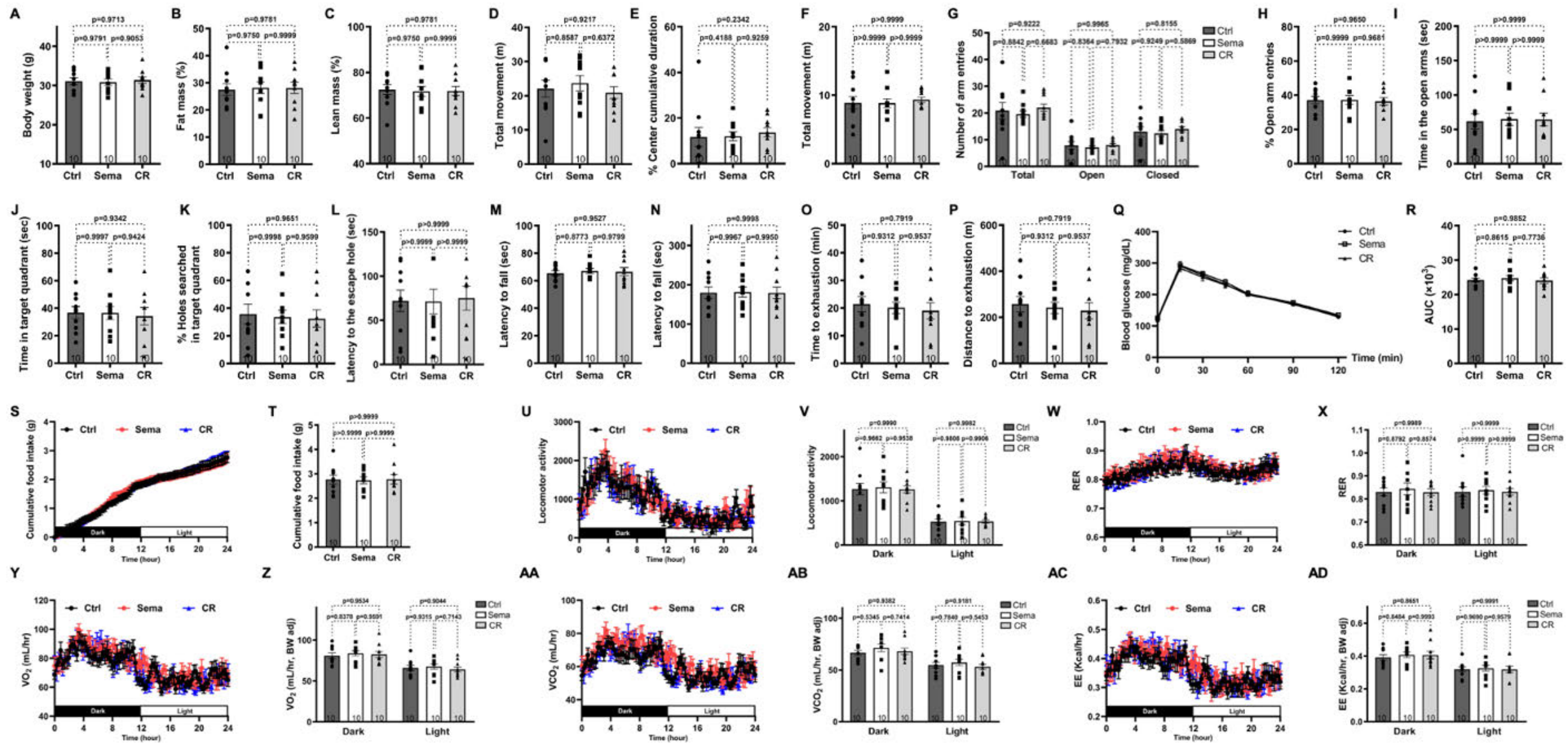

A longitudinal study of 20-month-old female C57BL/6 mice treated with vehicle, semaglutide, or calorie restriction for 5 months. Data shown are baseline physiological measures. n=10.
A, Body weight.
B, C, The percentage of fat mass (B) and lean mass (C) in the Echo MRI test.
D, E, Total movement (D) and the percentage of center cumulative duration (E) in the open field test.
F-I, Total movement (F), number of arm entries (G), the percentage of open arm entries (H), time in the open arms (I) in the elevated plus maze test.
J-L, Time in target quadrant (J), percentage of holes searched in target quadrant (K), latency to the escape hole (L) in the Barnes maze test.
M, Latency to fall in the rotarod test.
N, Latency to fall in the inverted screen test.
O, P, Time (O) and distance to exhaustion (P) in the treadmill exhaustion test.
Q, R, Blood glucose (Q) and the area under the curve (AUC) (R) in the glucose tolerance test.
S-AD, Measurements in metabolic cage.
S, T, Cumulative food intake (S) and summarized (T).
U, V, Locomotor activity (U) and summarized by light and dark cycles (V).
W, X, Respiratory exchange ratio (W) and summarized by light and dark cycles (X).
Y, Z, Oxygen consumption absolute value (Y) and summarized by light and dark cycles after adjustment using ANCOVA with body weight as a covariate (Z).
AA, AB, Carbon dioxide production absolute value (AA) and summarized by light and dark cycles after adjustment using ANCOVA with body weight as a covariate (AB).
AC, AD, Energy expenditure absolute value (AC) and summarized by light and dark cycles after adjustment using ANCOVA with body weight as a covariate (AD).

Both semaglutide treatment and calorie restriction led to comparable body weight and fat loss (Data below A-C)(Figure S10A-C).

A

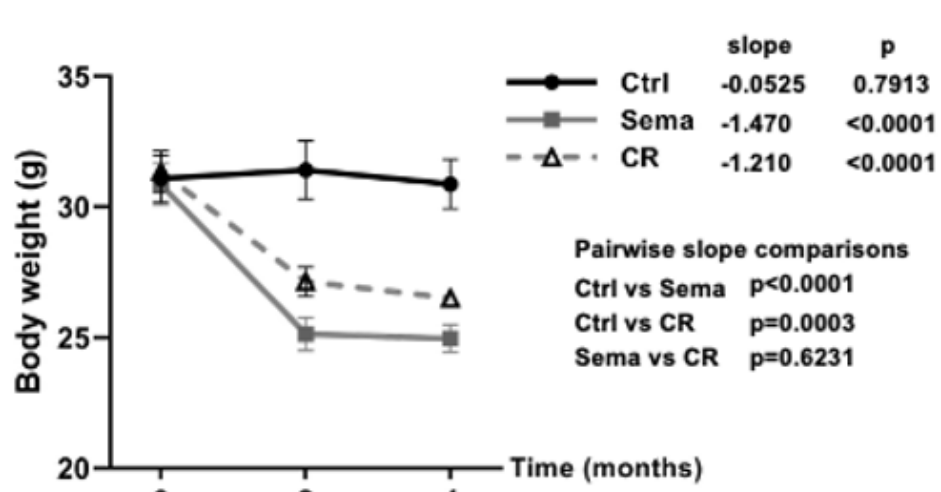

B

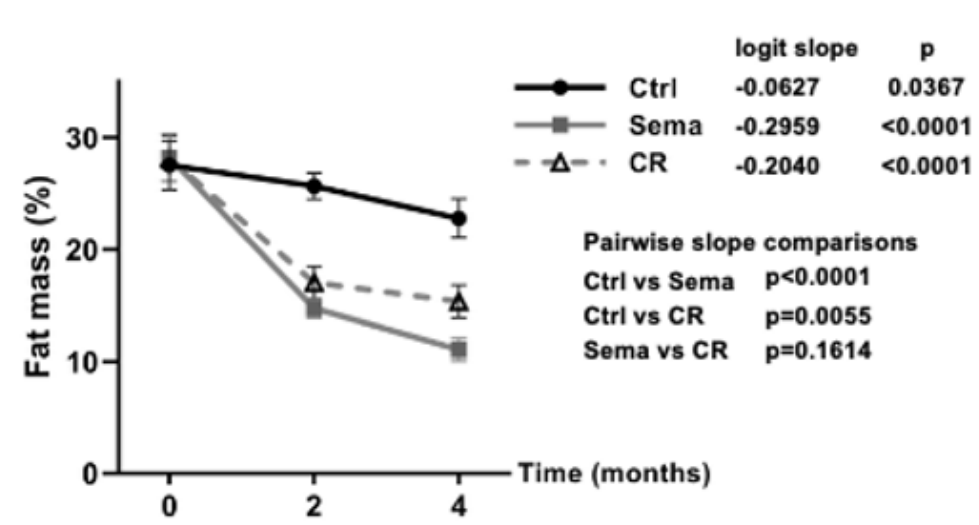

C

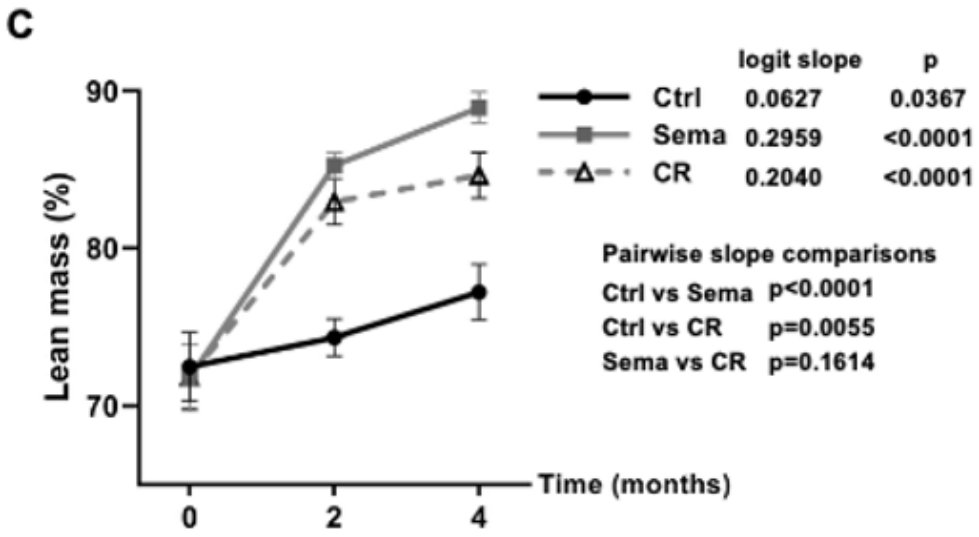

Comparison of 20-month-old female C57BL/6 mice treated with vehicle, semaglutide, or calorie restriction for 5 months. Mice were assessed at the baseline or after 2 or 4 months of treatment. n=10.
A, Body weight.
B, C, The percentage of fat mass (B) and lean mass (C) in the Echo MRI test.

Control mice exhibited progressive declines in locomotor activity and exploratory behavior in novel environments, motor coordination, muscle function, glucose control, and spatial memory over the course of the longitudinal study (Data below A-I)(Figure 5). Semaglutide treated mice showed significantly different trajectories from control mice across measures (Data below A-I)(Figure 5). Semaglutide treatment preserved baseline function and produced trajectories comparable to those of calorie restricted mice for total locomotor activity in the open field (Data below A)(Figure 5A) and elevated plus maze tests (Data below C)(Figure 5C), rotarod performance (Data below F)(Figure 5F), inverted screen performance (Data below G)(Figure 5G), and treadmill endurance (Data below H)(Figure 5H). Notably, semaglutide treatment led to improvements above baseline, and to more favorable trajectories than calorie restriction, in open field center time (Data below B)(Figure 5B), Barnes maze performance (Data below E)(Figure 5E), and glucose tolerance (Data below I)(Figure 5I). These findings indicate that GLP-1R activation late in life recapitulates many functional benefits of calorie restriction by attenuating age-associated decline, while conferring additional benefits in exploratory drive, spatial memory, and glucose control, suggesting effects beyond reduced calorie intake alone.

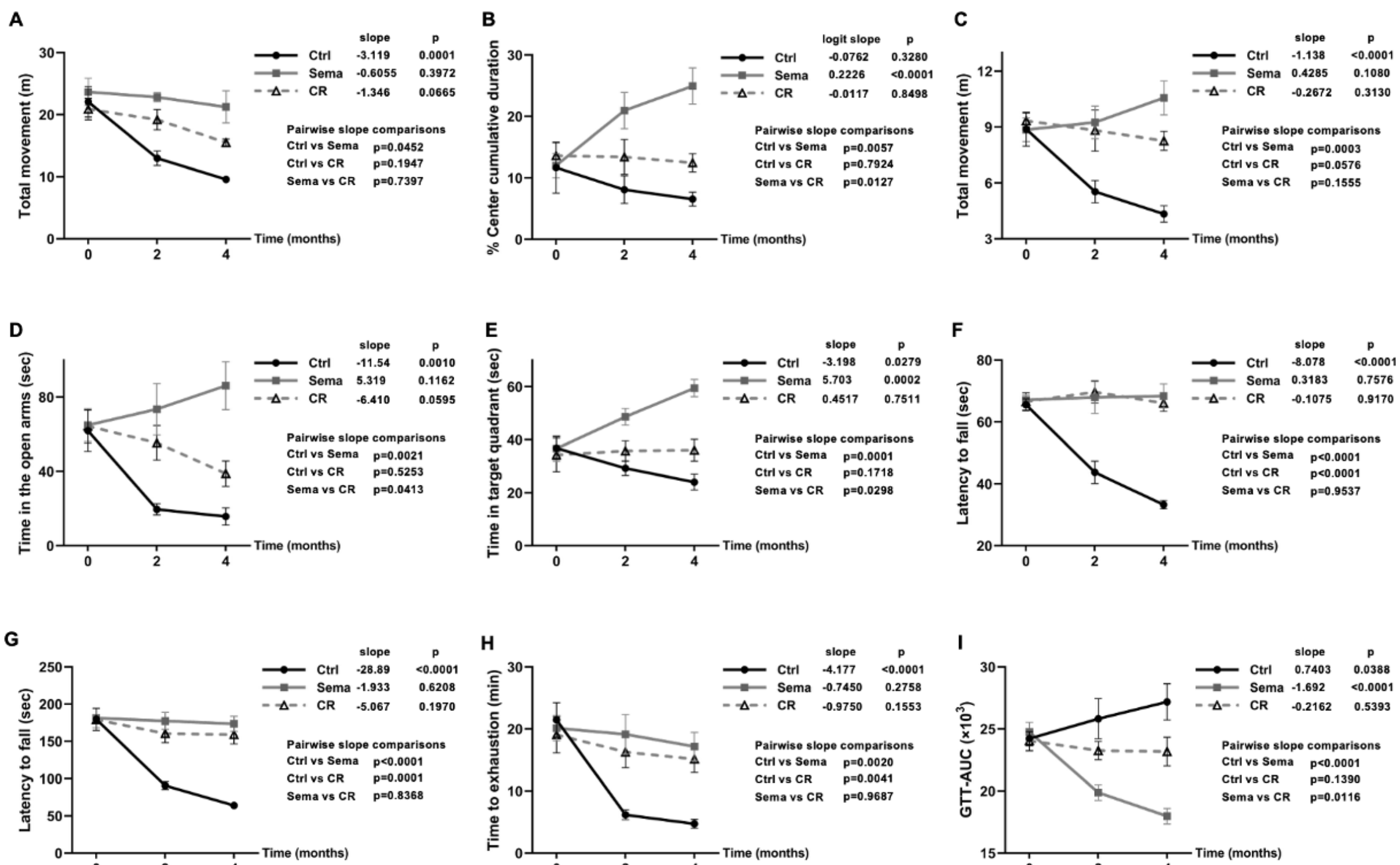

Comparison of 20-month-old female C57BL/6 mice treated with vehicle, semaglutide, or calorie restriction for 5 months. Mice were assessed at the baseline or after 2 or 4 months of treatment. n=10.
A, B, Total movement (A) and the percentage of center cumulative duration (B) in the open field test.
C, D, Total movement (C) and time in the open arms (D) in the elevated plus maze test.
E, Time in target quadrant in the Barnes maze test.
F, Latency to fall in the rotarod test.
G, Latency to fall in the inverted screen test.
H, Time to exhaustion in the treadmill exhaustion test.
I, The glucose tolerance test.

7. Fig. S1a: Could the authors provide the body weight trajectories of each experimental group before starting the treatment? Please indicate if the trajectories of body weight correspond to all animals included in the lifespan analysis or if it is a subgroup of animals. It is also important to note that for the age-range shown in this figure (600-780 days of age), it would be expected that control animals start to show a modest decline in body weight (https://doi.org/10.1016/j.cmet.2021.08.013). Discussing these results in light of the available literature for C57BL/6 female mice is strongly recommended. Also, why do the authors choose to show the body weight trajectories in that age-range instead of the entire age-range covered in the lifespan analysis (600-860 days of age)? Why isn't there a statistical analysis associated with these body weight trajectories?

Response: We thank the reviewer for this helpful suggestion. We have revised Fig. S1a (Data below)(New Figure S2A) and the corresponding legend/text as follows.

1. We have included body weight trajectories prior to treatment initiation in New Figure S2A (Data below). Baseline body weight did not differ between groups (p= 0.147).
2. The body weight trajectories in Fig. S1a (New Figure S2A) reflect all animals included in the lifespan cohort. This is now explicitly stated in the figure legend.
3. We agree that a modest late-life decline in body weight has been reported in aging C57BL/6 mice (Palliyaguru, Cell Metab 2021). However, other studies have documented stable body weight beyond 20 months (Fischer, Aging 2016; Ogiso, Exp Anim 2024; Johnson, Aging Pathobiol Ther 2023; Ge, Pathobiology of Aging & Age-related Diseases 2016; Pajski, J Gerontol A Biol Sci Med Sci 2024), consistent with our observations. We have included this discussion in the revised manuscript.
4. We are not including body weight data beyond 810 days because very few (4) control mice remained, making group-level summaries increasingly unstable and formal inference uninformative.
5. We have now added statistical analysis for the body weight data in the Figure and Table S3 for statistical analyses.

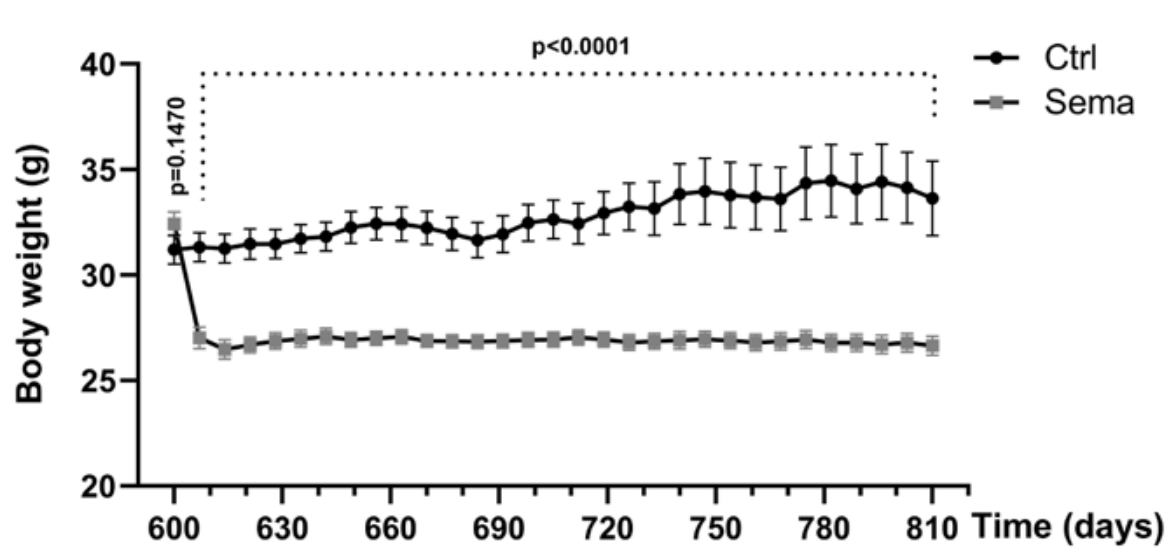

20-month-old female C57BL/6 mice were treated with vehicle or semaglutide for the duration of the lifespan. Body weight of mice in the lifespan study. n=39,40.

8. Fig. 1l,m: In parallel with the analyses of the absolute values of the rotarod and grip strength (sic) tests currently shown, please provide these data analyzed using ANCOVA including body weight as a covariate in the estimation of performance on these tests.

This will inform whether the physical performance improvement in the semaglutide group is independent or mediated by body weight/lean mass.

Response: To determine whether improvements in rotarod and inverted screen performance were independent of body weight, we performed ANCOVA with body weight included as a covariate. After adjustment for body weight, the semaglutide group retained a significant increase in latency to fall compared with controls (Data below A, B)(Figure S3H, I).

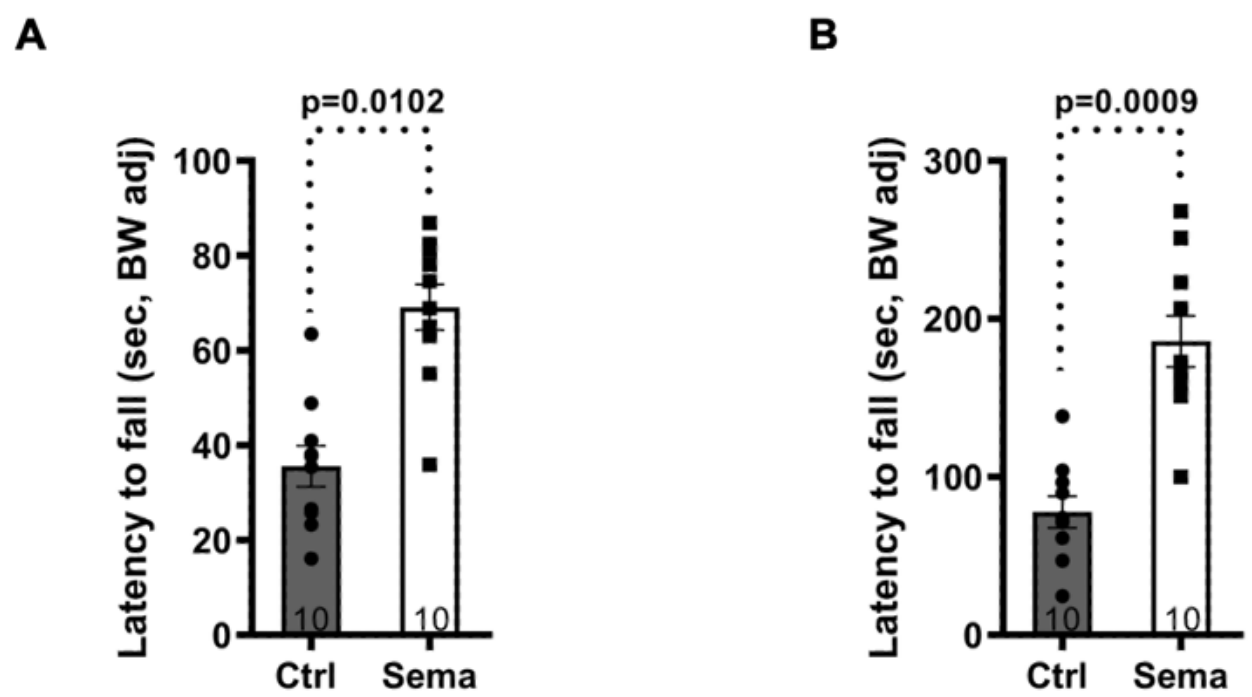

20-month-old female C57BL/6 mice were treated with vehicle or semaglutide for 3 months. n=10.
A, Latency to fall in the rotarod test after adjustment using ANCOVA with body weight as a covariate.
B, Latency to fall in the inverted screen test after adjustment using ANCOVA with body weight as a covariate.

9. Fig. S2: Based on the VO2 and energy expenditure values provided, mice treated with semaglutide would have increased metabolism. However, since the values are presented as ratios to body weight (Kcal/h/kg or ml/h/kg), the leaner semaglutide-treated animals may show apparently higher EE and VO2 only because of how this ratio is calculated and not because they are actually consuming more oxygen. Also, from a statistical standpoint, using the ratio Energy Expenditure/Body Weight can introduce bias if the relation between EE and BW includes an intercept different than zero (i.e., if it is not purely linear). Thus, it is recommended to present the absolute values of these outcomes (Kcal/h or ml/h) in parallel with data adjusted by lean mass (physiologically more relevant than body weight) or body weight as covariates through an appropriate statistical method such as ANCOVA (see for example DOI https://doi.org/10.1038/s41586-024-08026-3). Such method allows to accurately compare experimental groups with different body weight or lean mass without introducing the bias of the method that uses the ratios. On the other hand, in addition to the trajectories of food consumption, please provide the carbon dioxide production levels, the respiratory exchange ratio and locomotor activity obtained with the CLAMS system, since these are fundamental measures to interpret the energy balance and type of substrate being used (fat vs carbohydrate). In addition, the findings emerging from these variables should be discussed more in depth, as they are critical for the authors' hypothesis regarding that semaglutide acts as a calorie restriction mimetic. How do these results align to those observed by indirect calorimetry under calorie restriction?

(see for example: https://doi.org/10.1038/s41586-024-08026-3; https://doi.org/10.1016/j.cmet.2023.05.003).

Response: We thank the reviewer for this important point. As suggested by the reviewer, we now present absolute values for oxygen consumption, carbon dioxide production, and energy expenditure, and have additionally analyzed these variables using ANCOVA with body weight as a covariate, as described (Francesco, Nature 2024). We have also included data for food intake, respiratory exchange ratio, and locomotor activity. Semaglutide treatment reduced food intake by 24%, but there were no significant differences in adjusted oxygen consumption, adjusted carbon dioxide production, adjusted energy expenditure, respiratory exchange ratio, and locomotor activity between groups (Data below) (Figure S1).

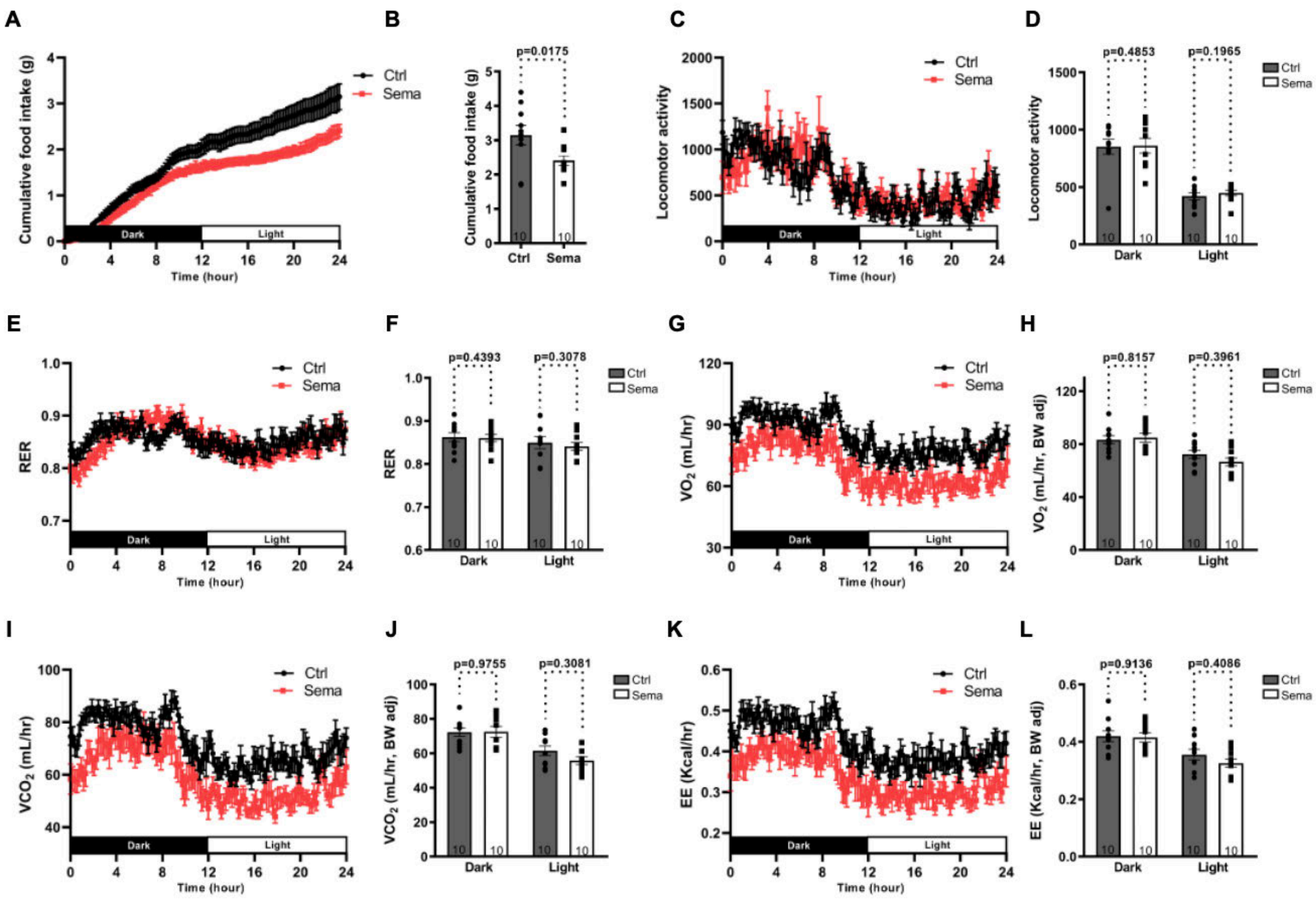

Comparison of 20-month-old female C57BL/6 mice treated with vehicle or semaglutide for 3 months in metabolic cage. n=10.
A, B, Cumulative food intake (A) and summarized (B).
C, D, Locomotor activity (C) and summarized by light and dark cycles (D).
E, F, Respiratory exchange ratio (E) and summarized by light and dark cycles (F).
G, H, Oxygen consumption absolute value (G) and summarized by light and dark cycles after adjustment using ANCOVA with body weight as a covariate (H).
I, J, Carbon dioxide production absolute value (I) and summarized by light and dark cycles after adjustment using ANCOVA with body weight as a covariate (J).
K, L, Energy expenditure absolute value (K) and summarized by light and dark cycles after adjustment using ANCOVA with body weight as a covariate (L).

To address the reviewer's point regarding comparison with calorie restriction, we also compared metabolic cage profiles of semaglutide treated mice and calorie restricted mice. Although the two groups had a similar daily food intake, their feeding patterns differed markedly (Data below A, B)(Figure S8S, T). Calorie restricted mice consumed

their daily food allotment soon after feeding and then underwent a prolonged fasting period, whereas semaglutide-treated mice consumed food more gradually throughout the day, consistent with appetite suppression. Correspondingly, calorie restricted mice showed an increase in locomotor activity late in the light cycle when anticipating the next feeding, consistent with foraging behavior, which was not observed in semaglutide treated mice (Data below C)(Figure S8U). Calorie restricted mice showed the expected dynamic changes in respiratory exchange ratio, with high values after feeding and lower values during the fasting phase (Data below E, F)(Figure S8W, X). In addition, calorie restricted mice showed comparable adjusted oxygen consumption, adjusted carbon dioxide production, adjusted energy expenditure during the dark cycle, but lower adjusted carbon dioxide production and trends toward lower adjusted oxygen consumption and adjusted energy expenditure during the light cycle (Data below G-L)(Figure S8Y-AD), consistent with the literature (Francesco, Nature 2024). These results indicate that semaglutide and calorie restriction produce a similar reduction in overall calorie intake through distinct mechanisms and physiological states. Calorie restriction is associated with hunger-driven temporal behavioral and metabolic adaptations, whereas semaglutide reduces intake in the context of suppressed appetite and is therefore not accompanied by the same feeding-associated rhythms.

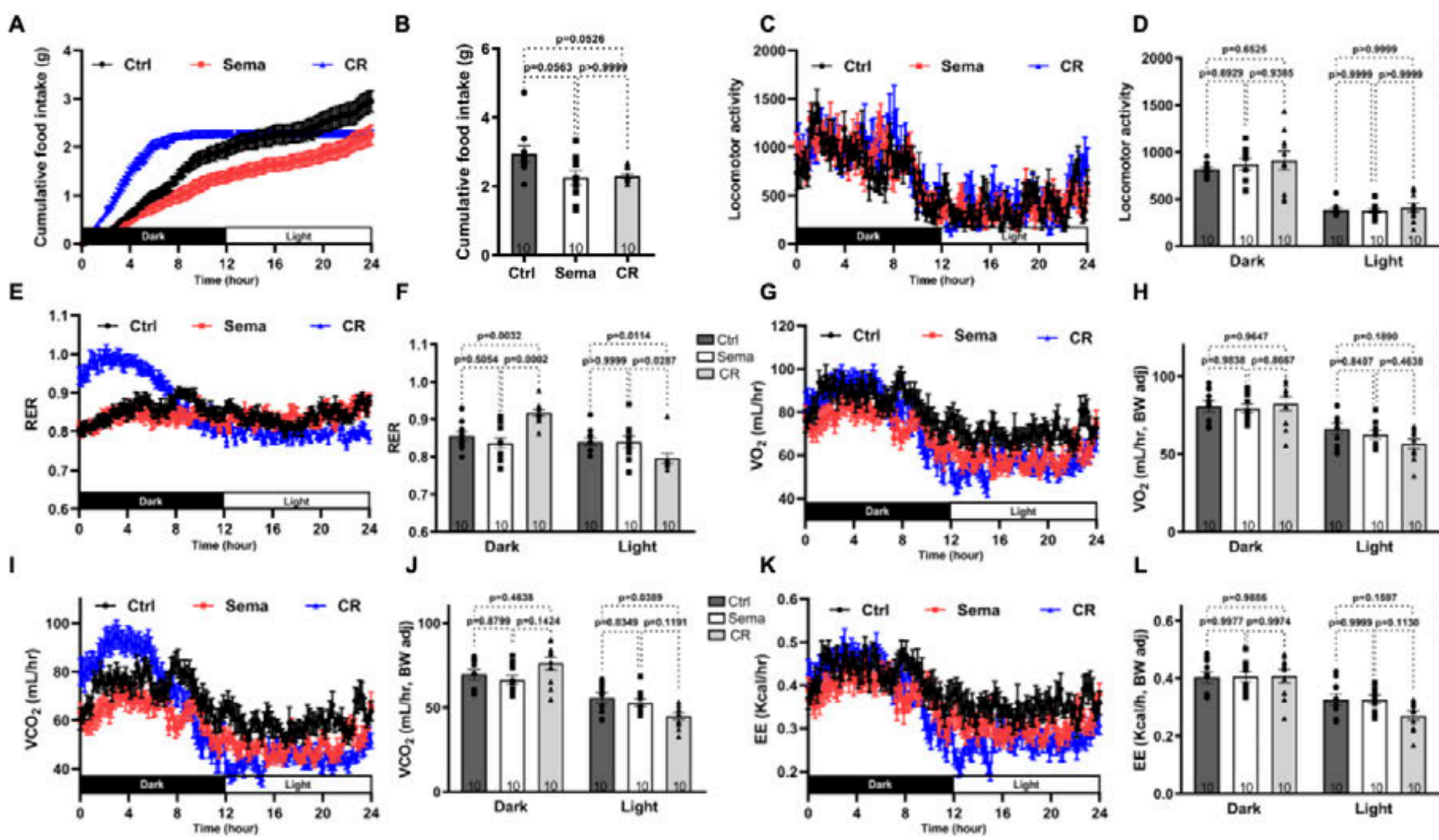

A longitudinal study of 20-month-old female C57BL/6 mice treated with vehicle, semaglutide, or calorie restriction for 5 months. Data shown are measures in metabolic cage after 2 months of treatment. n=10.
A, B, Cumulative food intake (A) and summarized (B).
C, D, Locomotor activity (C) and summarized by light and dark cycles (D).
E, F, Respiratory exchange ratio (E) and summarized by light and dark cycles (F).
G, H, Oxygen consumption absolute value (G) and summarized by light and dark cycles after adjustment using ANCOVA with body weight as a covariate (H).
I, J, Carbon dioxide production absolute value (I) and summarized by light and dark cycles after adjustment using ANCOVA with body weight as a covariate (J).
K, L, Energy expenditure absolute value (K) and summarized by light and dark cycles after adjustment using ANCOVA with body weight as a covariate (L).

10. Several statements and conclusions regarding the mechanisms and effects

proposed to be modulated by semaglutide are based on mRNA expression only (e.g., “GLP-1R activation improves mitochondrial function”). Modulation of gene expression levels by semaglutide is an important result indicative of its mechanism of action, but without functional measures (protein levels, enzymatic activity, among others), it is not possible to make firm conclusions on that regard. For example, in Fig. S7, the increased mRNA levels of Pgc1a, Nrf1, Tfam, Mfn1, Mfn2, ND1, ND4, Cytb, Cytc, Atp6 y Cox1 are indicative of improved mitochondrial functon(in terms of biogenesis, dynamics, respiratory capacity, etc.). However, to firmly state that mitochondrial function is improved it is necessary to confirm these findings with functional measures such as respiratory activity, ATP production, protein levels, etc. The same is valid for Figure S8, where the increased mRNA levels of Nrf2, Cat, Hmox1 and Nqo1 in the semaglutide group is an indicator that the antioxidant response is active and potentially improved, but these transcriptomic measures need to be complemented with protein levels and enzymatic activity (e.g., CAT, HO-1, NQO1, reduction of ROS and markers of oxidative stress).

Response: We thank the reviewer for this important point. We agree that changes in mRNA expression alone are insufficient to make definitive conclusions about mitochondrial functional capacity or oxidative stress response. As suggested by the reviewer, we performed functional assays to complement the mRNA expression results in Fig. S7 and Fig. S8 (New Figure S5). To assess mitochondrial function, we quantified ATP levels and found that semaglutide treatment increased ATP content (Data below A)(Figure S5G). To assess oxidative stress response, we measured cellular ROS using MitoSOX staining and found that semaglutide treatment reduced ROS levels (Data below B)(Figure S5H). Together, these functional readouts support the interpretation that the observed increases in mitochondrial and oxidative stress response transcripts are associated with improved mitochondrial energetic status and reduced oxidative stress in semaglutide-treated mice.

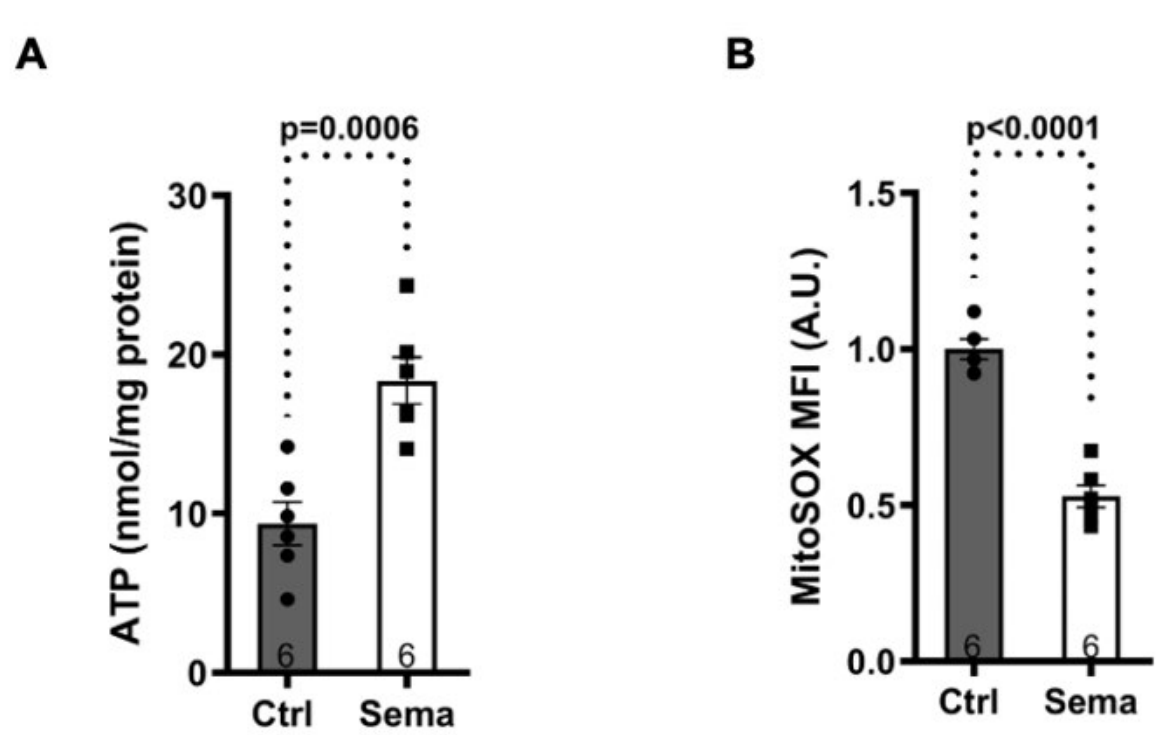

Comparison of 20-month-old female C57BL/6 mice treated with vehicle or semaglutide for 3 months.
A, Quantification of ATP in the muscle. n=6.
B, Quantification of flow cytometry analysis of MitoSOX staining of HSCs. n=6.

11. Minor comment: The color of the treatments in Fig. S5e,g is inverted with regards to the rest of the figures of the manuscript.

Response: We have changed the color for consistency.

Discussion section

1. The findings of this study should be discussed more in depth, both in terms of similarities and discrepancies with previous studies on GLP-1 receptor agonists and regarding the authors' hypothesis that semaglutide acts as a calorie restriction mimetic.

Response: We have expanded the Discussion section to put our study in the context of the existing literature. Preclinical studies in disease mouse models (Petersen, Nature 2024; Ma, Nature Communications 2024; Sourris, Kidney International 2024; Jara, Nature Medicine 2025; Zhang, Nature Aging 2025) and clinical studies (Garvey, Nature Medicine 2022; Weghuber, The New England Journal of Medicine 2022; Lincoff, The New England Journal of Medicine 2023; McGuire, The New England Journal of Medicine 2025; Apperloo, Nature Medicine 2025; Perkovic, The New England Journal of Medicine 2024; Sanyal, The New England Journal of Medicine 2025; Athauda, Lancet 2017; Meissner, The New England Journal of Medicine 2024) have revealed pleiotropic beneficial effects of GLP-1R activation, including improved glucose and weight control, and reduced cardiovascular, renal, hepatic, and neurodegenerative disease burden. We found that GLP-1R activation late in life improved physiological function and extended lifespan in an aging mouse model (20-month-old female C57BL/6) treated with a defined semaglutide regimen that reduced food intake by 24%.

We have also discussed how our data support the hypothesis that semaglutide acts as a calorie restriction mimetic. Our findings establish that GLP-1R activation late in life alleviates broadly aging-associated decline and phenocopies the molecular and physiological benefits of calorie restriction. In aged mice, semaglutide treatment extended lifespan, improved physiological function (Figure 1, S2, S3), reduced hallmarks of aging such as stem cell attrition, inflammation, cellular senescence, genomic instability, mitochondrial dysfunction, and loss of proteostasis (Figure 2, 3, S4-S6), and modulated the genetic regulators of aging and nutrient sensors in the same fashion as calorie restriction (Figure 4). Direct comparison of semaglutide treatment with matched calorie restriction further showed comparable effects across several aspects of aging-associated physiological decline (Figure 5A, C, F, G, H), consistent with the idea that GLP-1 medicines can act as calorie restriction mimetics. Because aging is the biggest risk factor for numerous chronic diseases and calorie restriction slows aging and ameliorates a broad spectrum of aging-associated diseases, our findings raise the possibility that GLP-1 medicines may influence a wide array of seemingly unrelated diseases by slowing aging.

Our study also reveals that GLP-1R activation exerts effects beyond those attributable to reduced calorie intake alone (Figure 5B, E, I). Whereas calorie restriction generally maintained exploratory drive, spatial memory, and glucose control near baseline levels,

semaglutide treatment significantly improved these outcomes above baseline, raising the possibility that GLP-1 medicines may not only slow but reverse aspects of aging-associated functional decline.

2. Do the authors recognize any limitations in their study? Are there any adverse effects of semaglutide?

Response: We thank the reviewer for raising this point. Our study was designed to test the effect of late-life GLP-1R activation on aging-associated decline at the molecular, cellular and organismal levels, and lifespan in an established mouse aging model (20-month-old C57BL/6) using a defined semaglutide regimen, enabling controlled assessment. Female mice were selected to minimize confounding effects of male aggression and injury, consistent with prior long-term aging studies (Francesco, Nature 2024). Within the endpoints monitored in this study, we did not observe adverse effects attributable to semaglutide. Importantly, while semaglutide is widely used clinically, whether GLP-1R activation modulates aging trajectories and lifespan in humans will require long-term clinical studies designed to evaluate aging-related outcomes in older populations. We have included this information in Discussion section of the manuscript.

Methods section

1. In the statistical analysis section, the authors indicate: “Mice were randomized to groups and analysis of mice and tissue samples were performed by investigators blinded to the treatment of the animals. Measurements were taken from distinct samples.” Please indicate if treatment groups had homogeneous body weights at randomization, the total number of animals used in the study, including all experiments, and the method to select the animals (if any) for the functional measures performed.

Response: This information is included in the Methods section. For the lifespan study, 39 mice received saline and 40 mice received semaglutide for the duration of the lifespan. For physiological, molecular, and cellular studies, a separate cohort was treated for 3 months. 10 pairs for physiological assessments, 5 pairs for neural stem cell studies requiring tissue fixation, and 6 pairs for other molecular and cellular analyses. For longitudinal study, a separate cohort was treated with saline, semaglutide, or calorie restriction for 5 months (10 mice per group).

There was no body weight difference at randomization in the experiments comparing mice treated with saline and semaglutide for lifespan (Data below A), physiological assessments (Data below B), neural stem cell studies (Data below C), and other molecular and cellular studies (Data below D). There was also no body weight difference at randomization in the longitudinal study comparing mice treated with saline, semaglutide, and calorie restriction (Data below E).

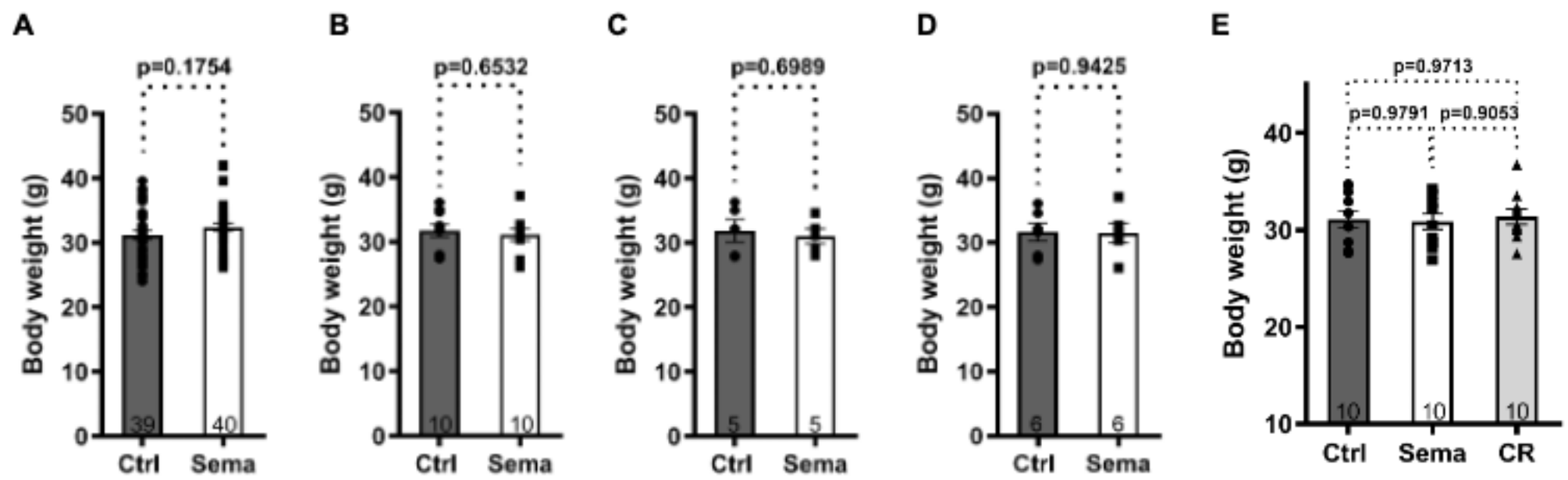

A, 20-month-old female C57BL/6 mice were treated with vehicle or semaglutide for the duration of the lifespan for lifespan study. Data shown are baseline body weight. n=39,40.
B-D, 20-month-old female C57BL/6 mice were treated with vehicle or semaglutide for 3 months for physiological assessments (B, n=10), neural stem cell studies (C, n=5), and other molecular and cellular analyses (D, n=6). Data shown are baseline body weight.
E, A longitudinal study of 20-month-old female C57BL/6 mice treated with vehicle, semaglutide, or calorie restriction for 5 months. Data shown are baseline body weight. n=10.

2. Please indicate in the methods section for how long the mice were acclimated to the same conditions after arrival at the facility and before starting the experimental treatments.

Response: We have included this information in the Methods section. “Mice were acclimated for a week after arrival at the facility and before starting the experimental treatments or baseline measurements”.

3. Please indicate in methods section how mice were housed (group-housed? single-housed?) as this factor greatly affects behavior towards food consumption and thermoregulation, which impact the overall energetic context, a key aspect for this study.

Response: We have provided this information in the Methods section. “As described (Francesco, Nature 2024), group housing is standard practice for calorie restriction studies. Mice were group-housed on a 12:12 h light:dark cycle at 25 °C. Control and semaglutide-treated groups had ad libitum access to food and water. Calorie restricted mice were provided with unlimited access to water and measured amounts of food daily. Competition for food was minimized by placing food directly into the bottom of the cage, allowing individual mice to get a pellet.”

4. Please provide all details describing how subcutaneous injections were performed (and corresponding reference of the protocol), as their short- and long-term effect on local inflammation, tissue damage, and ulceration may not be trivial for several of the biomarkers measured in this study as well as from an ethical standpoint. Did the operator rotate the injection sites throughout the study?

Response: We thank the reviewer for raising this important point. We have included this information in the Methods section. “For subcutaneous injection, we followed the standard operating protocol established by the animal care committee at the University of California, Berkeley. Briefly, subcutaneous injections were performed daily using

a 28-gauge needle in the dorsal subcutaneous region. To reduce discomfort, a new needle was used for each animal. To minimize local tissue irritation and the risk of chronic inflammation, the operator systematically rotated injection sites across the dorsal surface, ensuring no single site was used repeatedly in consecutive days. Animals were monitored daily for local reactions, including erythema, swelling, ulceration, or tissue damage. No significant adverse effects were observed at any time point".

5. What the authors call "Grip Strength Test" in literature is commonly referred to as "cage top test", "inverted screen test", "grid hanging test", "hanging test", "cage wire test" (see: DOI: 10.1126/science.aat3987; 10.1038/bonekey.2015.101; https://doi.org/10.1038/s41596-019-0256-1), among other synonyms. It is strongly recommended to refer to this test with one of these nomenclatures, since the "grip strength test" measures the force (itself) of the limbs obtained with a grip strength meter and, thus, it is not a latency to fall.

Response: We thank the reviewer for pointing this out. We have changed to inverted screen test.

6. Most (if not all) statistical analyses in the manuscript were performed through t-tests, which require that the assumptions of normality and homoscedasticity are satisfied. Did the authors test these assumptions for each variable? How? If so, this has not been described in the statistical analysis section and should be provided, indicating by which method this was achieved. Visual inspection (e.g., Fig 1H, Fig. 4e-Sirt5, to name just two) and the nature of some variables (counts, percentages) suggest that the assumptions of this test could have been violated, and for such cases it is strongly recommended to perform estimations using models that are appropriate for the natural distribution of the variables (e.g., Poisson distribution for variables that are counts), tests that allow heterogeneous variances between groups (e.g., Welch's t-test?) or non-parametric tests (e.g., Mann–Whitney U, Wilcoxon signed-rank) as needed. In addition, please provide the exact p-values as well as the coefficients of the statistics used for each test.

Response: We thank the reviewer for the important comment. We have reassessed all analyses. Data were tested for normality using the Shapiro-Wilk test. Normally distributed data were tested for homoscedasticity using F-test or Brown-Forsythe test. For two-group continuous data, non-normally distributed data were analyzed using the Mann-Whitney U test. Student's t-test was used for data with equal variances and Welch's t-test was used for data with unequal variances. For three-group continuous data, Kruskal-Wallis test was used for non-normally distributed data, followed by Dunn's multiple comparisons test, one-way ANOVA for normally distributed data with equal variances, followed by Tukey's multiple comparisons test, and Welch's ANOVA for normally distributed data with unequal variances, followed by Games-Howell's multiple comparisons test. Covariate-adjusted analyses were performed using ANCOVA. Count

data were analyzed using generalized linear models with Poisson or negative binomial distribution when there was overdispersion. Percentage data were analyzed using beta regression. GTT data were analyzed using two-way repeated-measures ANOVA followed by Sidak's multiple comparisons between groups at each time point. Lifespan data were analyzed using the log-rank test. Categorical distribution data were analyzed using the Fisher-Freeman-Halton exact test. Weekly body weight data were analyzed using linear mixed-effects models, followed by Holm-adjusted multiple comparisons between groups at each time point. Other longitudinal continuous data were analyzed using linear mixed-effects models to assess aging trajectories. Slopes for each group were determined and pairwise comparisons of slopes were performed with Tukey adjustment. Longitudinal percentage data were analyzed using beta mixed-effects models to assess aging trajectories, with pairwise comparisons of slopes performed using Tukey adjustment. The exact p-values and the coefficients are provided in Figures and Table S3.

Referee #3 (Remarks to the Author):

I co-reviewed this manuscript with one of the reviewers who provided the listed reports.

Referees' comments:

Referee #1 (Remarks to the Author):

This paper describes studies of a GLP-1 receptor agonist, semaglutide, on parameters of aging in mice. The underlying hypothesis driving this work is that effects of semaglutide to reduce feeding mimics the effects of dietary caloric restriction that has been shown to promote longevity. The principal findings reported are that: 1) semaglutide administration to 20 week old mice extended lifespan by ~ 14 % compared to control treated mice; 2) semaglutide mice performed significantly better in tests of aging such as locomotor activity, muscle strength, and cognition; 3) semaglutide improved glucose tolerance and respiratory capacity; 4) semaglutide had salutary effects on established cellular and tissue markers of aging including stem cell dynamics, proteostasis, inflammation and mitochondrial function. The authors conclude from these results that semaglutide phenocopies the effects of caloric restriction to extend the lifespan and healthspan of mice.

This is an interesting set of observations on a timely and potentially impactful topic. GLP-1-based drugs are available and more refined versions with greater potency and ease of use are in development. These compounds are highly effective for treating diabetes, mitigate several co-morbidities of obesity such as NAFLD and obstructive sleep apnea, and are now the most potent weight loss drugs on the market. The demonstration that chronic treatment with semaglutide extends lifespan and quality adds a novel and important therapeutic application. The data presented in this paper make this demonstration in a convincing manner. The experiments are straightforward, and most have been used in previous studies of aging and caloric restriction. The sample sizes are adequate and the key effect sizes (e.g. longevity, weight loss) are meaningful. This is a paper that will have broad interest.

Response: We thank the reviewer for their interest in our study and for describing it as “timely and potentially impactful”, noting it “adds a novel and important therapeutic application”, and that we “make this demonstration in a convincing manner”.

The short-coming of this research is that these outcomes are mostly predictable. It is well known that semaglutide reduces food intake and body weight in rodents, humans and non-human primates. Thus, it is not surprising that medical caloric restriction has many features of dietary caloric restriction. In fact, it would have been surprising if semaglutide did not share many hallmarks of chronic food restriction. The study is essentially descriptive, although the phenomenon in question is interesting. It seems likely that many of the outcome measures here are a function of negative energy balance, although it is possible that some were mediated directly at the GLP-1 receptor in specific tissues; this question is not explored. Different approaches could have added depth to the results by trying to identify differences between semaglutide and caloric restriction, e.g. a matched calory restriction comparator, or even the use of doses of semaglutide that do not cause substantial weight loss.

Response: We thank the reviewer for their thoughtful comment. While the pro-longevity effects of dietary calorie restriction are well established, the benefits are not universal. The effects of calorie restriction are influenced by individual characteristics, including factors such as age and health condition (Francesco, Nature 2024; Mitchell, Cell Metabolism 2016; Ingram & de Cabo, Ageing Res Rev 2017; Forster, FASEB J 2003; Lipman, Aging Clin Exp Res 1995).Therefore, the long-term consequences of GLP-1R activation cannot be assumed, especially when treatment is initiated late in life. Our study directly addresses this outstanding question. We have included this clarification in the introduction.

We thank the reviewer's suggestion to compare directly GLP-1R activation and matched calorie restriction. We performed a longitudinal study in 20-month-old mice that were treated with vehicle, semaglutide, or matched (24%) calorie restriction for 5 months, and assessed at baseline and after 2 and 4 months of treatment. Both semaglutide treatment and calorie restriction led to comparable body weight and fat loss (Data below A-C) (Extended Data Figure 10a-c).

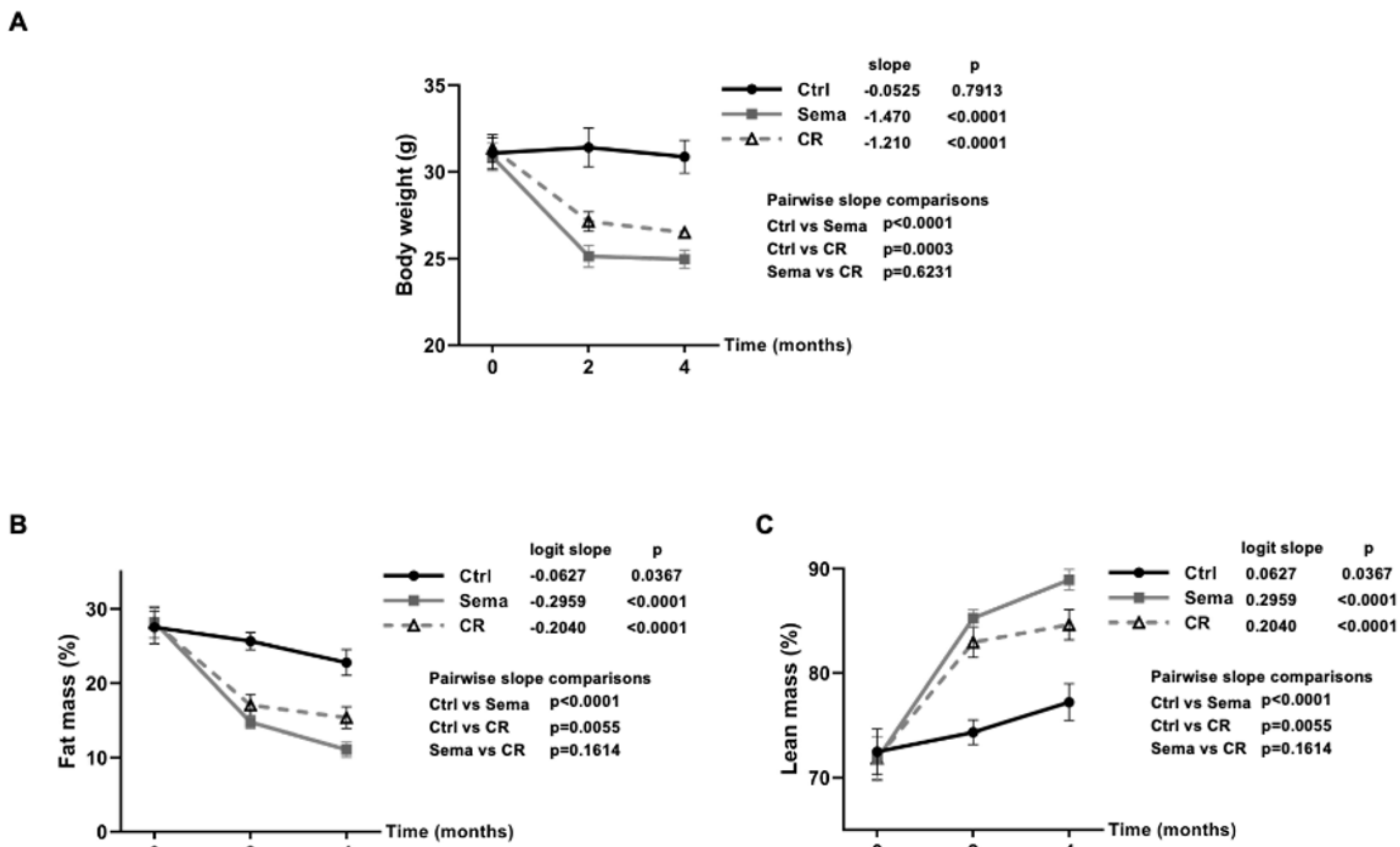

Comparison of 20-month-old female C57BL/6 mice treated with vehicle, semaglutide, or calorie restriction for 5 months. Mice were assessed at the baseline or after 2 or 4 months of treatment. n=10.
A, Body weight.
B, C, The percentage of fat mass (B) and lean mass (C) in the Echo MRI test.

Control mice exhibited progressive declines in locomotor activity and exploratory behavior in novel environments, motor coordination, muscle function, glucose control, and spatial memory over the course of the longitudinal study (Data below A-I)(Figure 5). Semaglutide treated mice showed significantly different trajectories from control mice across measures (Data below A-I)(Figure 5). Semaglutide treatment preserved baseline function and produced trajectories comparable to those of calorie restricted mice for total locomotor

activity in the open field (Data below A)(Figure 5a) and elevated plus maze tests (Data below C)(Figure 5c), rotarod performance (Data below F)(Figure 5f), inverted screen performance (Data below G)(Figure 5g), and treadmill endurance (Data below H)(Figure 5h). Notably, semaglutide treatment led to improvements above baseline, and to more favorable trajectories than calorie restriction, in open field center time (Data below B)(Figure 5b), Barnes maze performance (Data below E)(Figure 5e), and glucose tolerance (Data below I)(Figure 5i). These findings indicate that GLP-1R activation late in life recapitulates many functional benefits of calorie restriction by attenuating age-associated decline, while conferring additional benefits in exploratory drive, spatial memory, and glucose control, suggesting effects beyond reduced calorie intake alone.

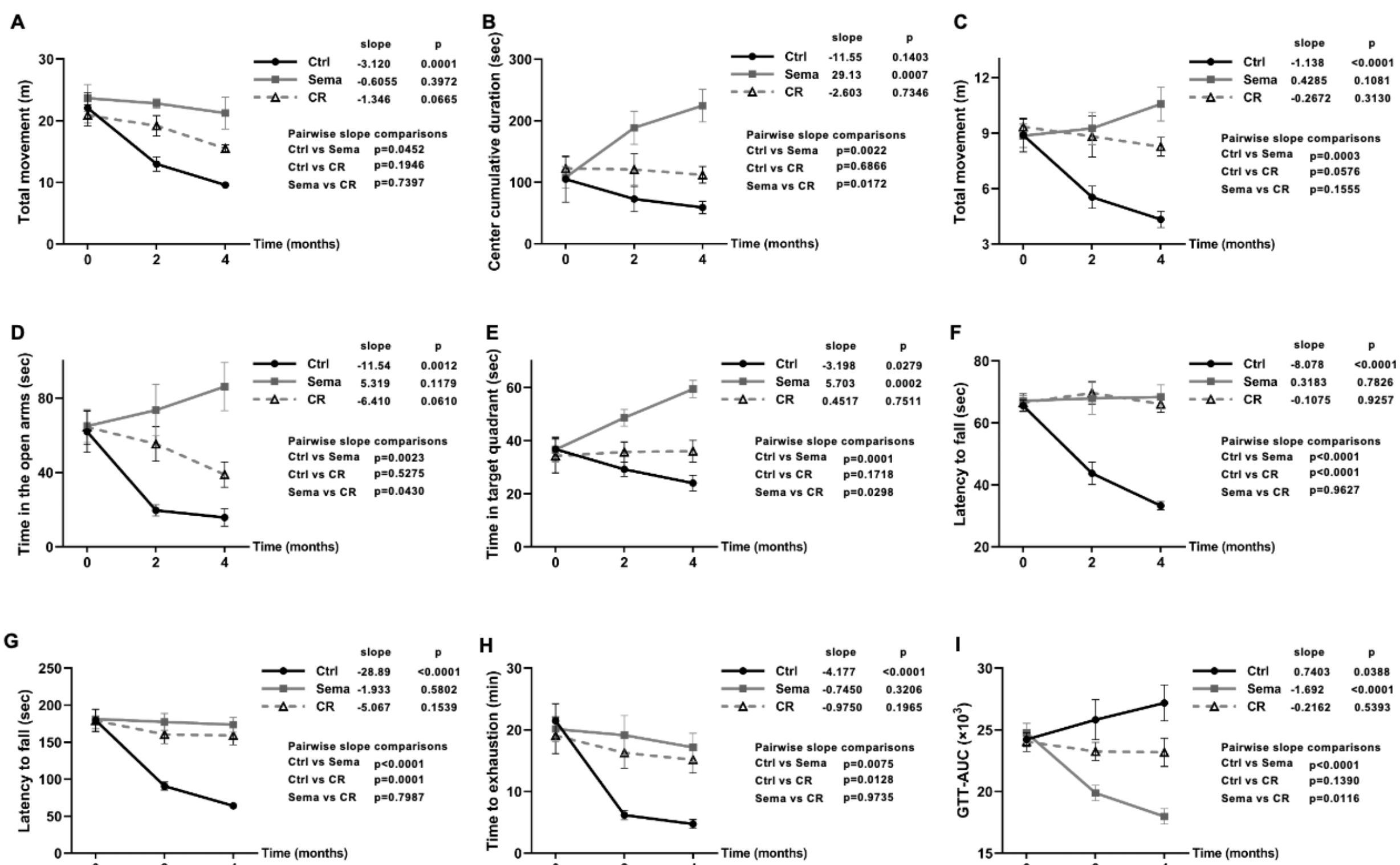

Comparison of 20-month-old female C57BL/6 mice treated with vehicle, semaglutide, or calorie restriction for 5 months. Mice were assessed at the baseline or after 2 or 4 months of treatment. n=10.
A, B, Total movement (A) and center cumulative duration (B) in the open field test.
C, D, Total movement (C) and time in the open arms (D) in the elevated plus maze test.
E, Time in target quadrant in the Barnes maze test.
F, Latency to fall in the rotarod test.
G, Latency to fall in the inverted screen test.
H, Time to exhaustion in the treadmill exhaustion test.
I, The glucose tolerance test.

As a minor point it was difficult to find a description of the semaglutide dose and treatment schedule in the text.

Response: We have included this information in “Methods” under “Mice”: “Mice received daily subcutaneous injection of 10 nmol/kg semaglutide or an equal volume of saline”.

[redacted]

Referee #2 (Remarks to the Author):

This study shows that daily subcutaneous injection of semaglutide (10 nmol/kg) in 20-month-old C57BL/6J female mice over three months modulates a range of core molecular, cellular, and functional metabolic biomarkers, indicating its potential to alleviate various hallmarks of aging. However, this requires further validation because many conclusions are based solely on mRNA expression levels, and functional products are not always measured. By continuing semaglutide injections until the end of the experimental period (about 28 months of age, which is the age at which the last control saline-injected animal died—quite young for this sex and strain), the authors demonstrate that this GLP-1 receptor agonist may extend median lifespan by approximately 13% in this sex and strain.

This work builds on earlier findings that show similar beneficial effects for semaglutide or other GLP-1 receptor activators, potentially indicating that this compound can also extend lifespan under “normal aging,” unlike previous studies that demonstrated this effect in mouse models of disease.
Overall, the manuscript is written clearly and is well-organized. The experiments designed, techniques used, and general methodology are solid. However, several aspects need revision, clarification, or expansion, both in terms of experimental or analytical validation and discussion enrichment, to effectively test the author’s hypothesis and support their main conclusion that semaglutide can serve as a calorie restriction mimetic, slowing aging and extending lifespan. These issues are outlined below.

Response: We thank the reviewer for pointing out the novel findings of our study under “normal aging” and that “the experiments designed, techniques used, and general methodology are solid”. We appreciate the constructive suggestions that have significantly improved our study.

General comments

1. It is recommended to define all abbreviations used throughout the manuscript at their first mention, regardless of how common they might be in the literature.

Response: We have defined abbreviations, as recommended.

2. It is recommended to indicate the sex and strain of the mice used in each and all figure legends.

Response: We have included sex and strain of the mice in all figure legends, as recommended.

Title section

1. The phrase “Slows Aging” may be too broad for this article because no longitudinal or baseline measures (before starting experimental treatments) have been taken for any variables except body weight. Additionally, healthspan cannot be assessed through endpoint analysis since it doesn't allow determination of when impairments begin. Furthermore, only “old” C57BL/6J female mice were used in this study, so the results cannot be generalized to other sexes, strains, or age groups. Therefore, the title should be rephrased to more accurately reflect what was actually studied.

Response: We thank the reviewer for raising these important points. We agree that claims regarding “slowing aging” should be supported by longitudinal measurements rather than endpoint analyses alone. To address this, we performed a longitudinal study in 20-month-old female C57BL/6 mice that were treated with saline, semaglutide, or matched (24%) calorie restriction for 5 months, and assessed at baseline and 2 and 4 months after intervention. No differences were observed among groups at baseline (Data below)(Extended Data Figure 7).

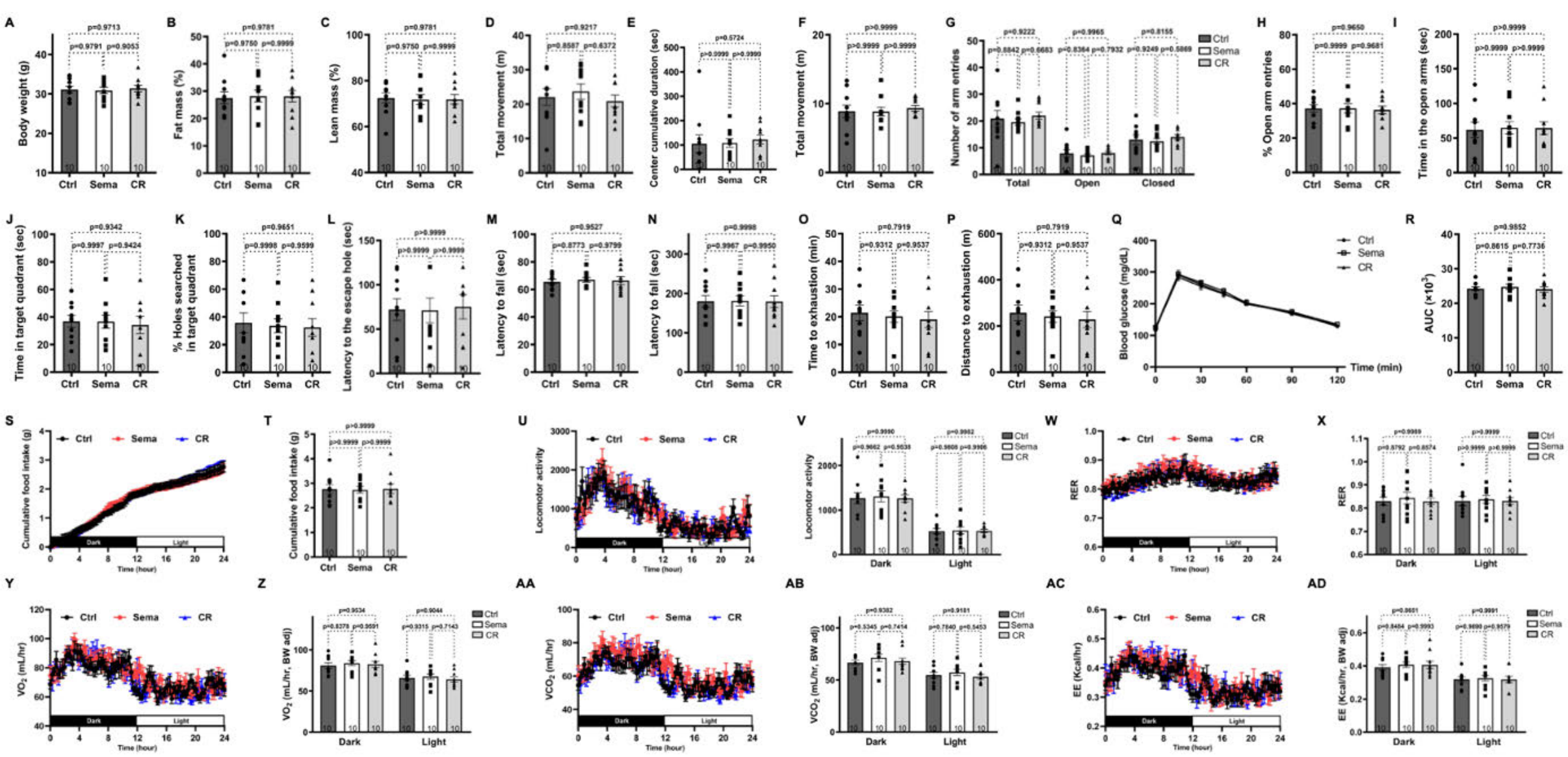

A longitudinal study of 20-month-old female C57BL/6 mice treated with vehicle, semaglutide, or calorie restriction for 5 months. Data shown are baseline physiological measures. n=10.
A, Body weight.
B, C, The percentage of fat mass (B) and lean mass (C) in the Echo MRI test.
D, E, Total movement (D) and center cumulative duration (E) in the open field test.
F-I, Total movement (F), number of arm entries (G), the percentage of open arm entries (H), time in the open arms (I) in the elevated plus maze test.
J-L, Time in target quadrant (J), percentage of holes searched in target quadrant (K), latency to the escape hole (L) in the Barnes maze test.
M, Latency to fall in the rotarod test.
N, Latency to fall in the inverted screen test.
O, P, Time (O) and distance to exhaustion (P) in the treadmill exhaustion test.
Q, R, Blood glucose (Q) and the area under the curve (AUC) (R) in the glucose tolerance test.
S-AD, Measurements in metabolic cage.
S, T, Cumulative food intake (S) and summarized (T).
U, V, Locomotor activity (U) and summarized by light and dark cycles (V).
W, X, Respiratory exchange ratio (W) and summarized by light and dark cycles (X).
Y, Z, Oxygen consumption absolute value (Y) and summarized by light and dark cycles after adjustment using ANCOVA with body weight as a covariate (Z).
AA, AB, Carbon dioxide production absolute value (AA) and summarized by light and dark cycles after adjustment using ANCOVA with body weight as a covariate (AB).
AC, AD, Energy expenditure absolute value (AC) and summarized by light and dark cycles after adjustment using ANCOVA with body weight as a covariate (AD).

Control mice exhibited progressive declines in locomotor activity and exploratory behavior in novel environments, motor coordination, muscle function, glucose control, and spatial memory (Data below A-I)(Figure 5). Semaglutide treated mice showed significantly different longitudinal trajectories from controls across measures, with some outcomes maintained at baseline levels and others significantly improved above

baseline (Data below A-I)(Figure 5). These findings indicate that late life GLP-1R activation attenuates age-associated physiological decline.

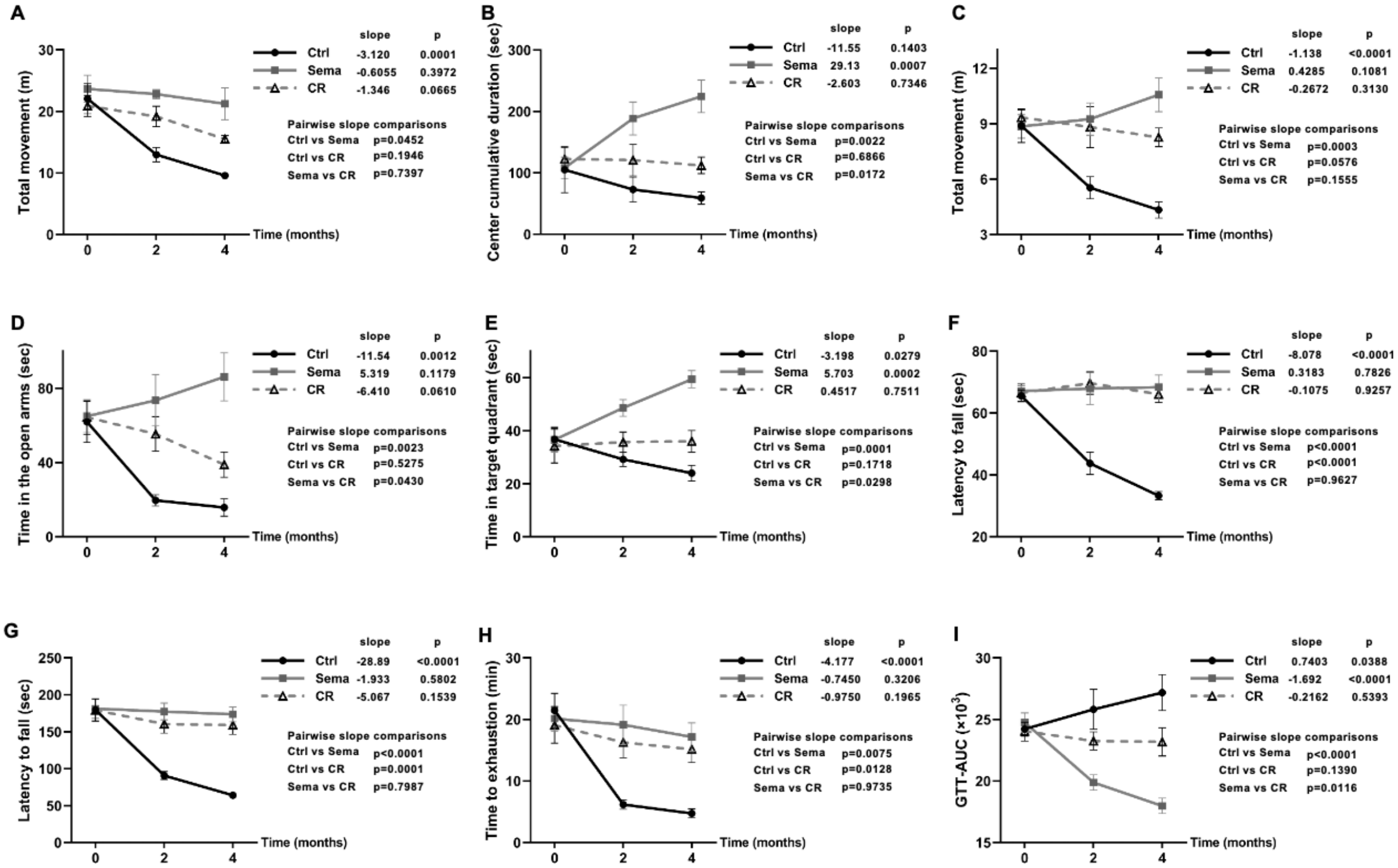

Comparison of 20-month-old female C57BL/6 mice treated with vehicle, semaglutide, or calorie restriction for 5 months. Mice were assessed at the baseline or after 2 or 4 months of treatment. n=10.
A, B, Total movement (A) and center cumulative duration (B) in the open field test.
C, D, Total movement (C) and time in the open arms (D) in the elevated plus maze test.
E, Time in target quadrant in the Barnes maze test.
F, Latency to fall in the rotarod test.
G, Latency to fall in the inverted screen test.
H, Time to exhaustion in the treadmill exhaustion test.
I, The glucose tolerance test.

All experiments were conducted in aged (20 months old) female C57BL/6 mice. The study was designed to test whether GLP-1R activation, a putative calorie restriction mimetic, can modify physiological decline and lifespan when initiated at advanced age. Because the effects of calorie restriction are influenced by individual characteristics, including factors such as age and health condition (Francesco, Nature 2024; Mitchell, Cell Metabolism 2016; Ingram & de Cabo, Ageing Res Rev 2017; Forster, FASEB J 2003; Lipman, Aging Clin Exp Res 1995), it is critical to determine how long-term use of GLP-1 medicines influences the aging process and lifespan, particularly when treatment is initiated late in life. Our study directly addresses this question. We have revised the title to reflect the scope of the study, emphasizing late-life intervention in mice.

Female mice were selected to minimize confounding effects of male aggression and injury, consistent with prior long-term aging studies (Francesco, Nature 2024). The Abstract, Methods, Figure Legends, and Discussion now clearly specify strain, sex, and age to ensure appropriate interpretation of the findings, and the title focuses on the biological question.

Abstract section

1. It is common practice to exclude references in the abstract of original articles, as their focus should be on what was done, the main results, and the conclusions, rather than on citing other work. I recommend removing references 1-11 from the abstract.

Response: Indeed, most journals require abstracts without references. However, Nature formatting guideline (https://www.nature.com/nature/for-authors/formatting-guide) states "articles start with a fully referenced summary paragraph, ideally of no more than 200 words, which is separate from the main text and avoids numbers, abbreviations, acronyms or measurements unless essential".

2. Please indicate the sex, strain, age, and number of mice used in this study, as well as the duration of treatment with semaglutide.

Response: We have included this information in the abstract. "Here, treatment of 20-month-old female C57BL/6 mice with semaglutide, a GLP-1R agonist, for three months improved physiological function, attenuated hallmarks of aging, and modulated nutrient sensors and conserved genetic regulators of aging. Continued treatment extended lifespan."

Introduction section

1. In the third sentence, the authors state: "Calorie restriction engages several evolutionarily conserved signaling pathways, such as sirtuins and insulin/IGF-1 signaling, to elicit a beneficial response." Please review the spelling of the word "illicit" (Did you mean "elicit"?).

Response: We thank the reviewer for pointing out the typo. We have corrected it.

Results section

1. At the beginning of the results section, the authors state: "We treated old (20 months old) mice daily with or without semaglutide, a GLP-1R agonist." Please specify what "treated" means (such as subcutaneous injection), as well as the sex and strain of the mice, and how long the animals received the semaglutide injections.

Response: We have added this information in the Results section. "We treated 20-month-old female C57BL/6 mice daily with either saline or semaglutide, a GLP-1R agonist, via subcutaneous injection. For lifespan study, treatment continued for the duration of the lifespan. For physiological, molecular, and cellular studies, a separate cohort was treated for three months."

2. Fig. 1a: The median and maximum lifespan of control saline-injected animals is quite short for this sex and strain (https://doi.org/10.1007/s11357-024-01226-

9; https://doi.org/10.1016/j.cmet.2021.08.013). Could the daily injections for approximately 260 days influence their normal lifespan? Alternatively, might this result be due to other factors, such as genetic or environmental conditions related to the facility where the experiment was conducted? Regardless, the lifespan results should be discussed in the context of existing literature. Ideally, the lifespan of the control saline-injected group should be compared with that of an intact control group kept under the same conditions but without saline injections. Additionally, the methods section describes conditions that classify an animal as severely moribund, justifying euthanasia. Please specify (1) how many animals experienced each of these conditions, (2) how these causes of death were distributed among the experimental groups (were they evenly distributed?), and (3) the ages at which these causes of death occurred. If animals that died from these reasons were included in the lifespan analysis, clarify whether they were censored or not. If censored, explain how this is represented in the Kaplan-Meier curves and accounted for in survival estimates. Also, state the statistical test used for survival analysis.

Response: We thank the reviewer for their insightful comment. Direct comparison from the National Institute on Aging (NIA) Interventions Testing Program (ITP) shows that lifespans differ substantially among the three ITP test sites (Harrison, Aging Cell 2013). As an example, site-specific medians for male control UM-HET3 mice were 704 days (UT), 807 days (TJL/JAX), and 924 days (UM), a difference of 220 days between UT and UM. This paper explicitly discusses that site-specific factors cause these large inter-site differences.

In the literature, median lifespans for C57BL/6 mice also vary widely: 550 days (Conti, Science 2006), 713 days (Baker, Nature 2016), 741 days (Latorre-Pellicer, Nature 2016), 750 days (Qu, Nature 2025), 760 days (Zhang, Nature 2023), 785 days (Mitchell, Cell Metabolism 2016). In the studies cited by the reviewer, medians were 805 days (Palliyaguru, Cell Metabolism 2021) and 867 days (Luciano, Geroscience 2024). Our observed median lifespan (742 days) falls comfortably within this reported range. We have included this discussion in the manuscript.

The distribution of endpoint categories was consistent with the literature, with high incidents of rectal prolapse for aging female C57BL/6 mice (Pettan-Brewer, Pathobiology of aging and age-related diseases 2011; Ogiso, Experimental Animals 2025), and did not differ significantly between groups (p=0.9431)(Data below A)(Extended Data Figure 3a). Age at death appeared to be delayed by semaglutide across several categories unrelated to tumor (Data below B-G)(Extended Data Figure 3b-g), consistent with a broader delay in age-associated physiological deterioration.

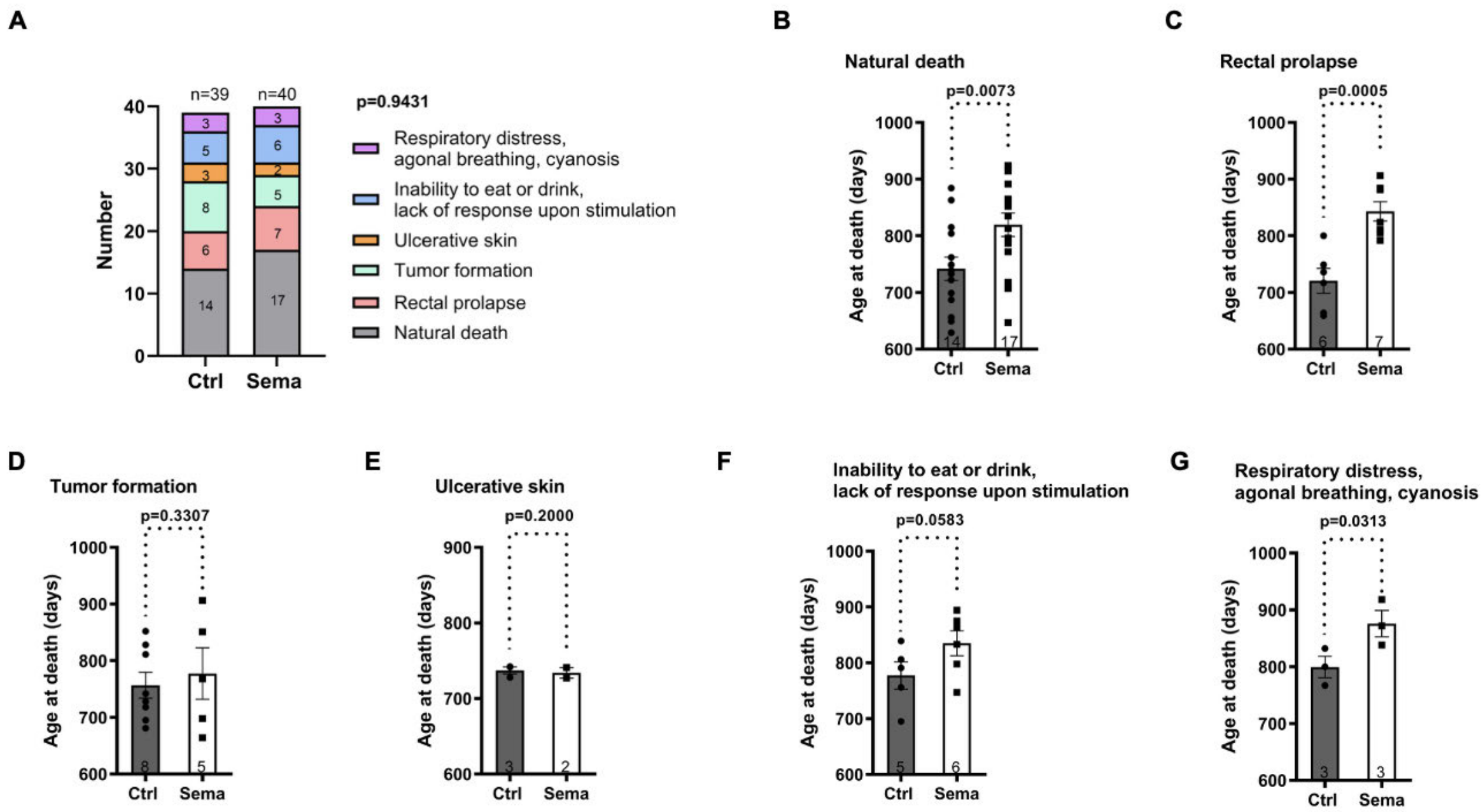

20-month-old female C57BL/6 mice were treated with vehicle or semaglutide.
A, Distribution of endpoint categories in the lifespan study. n=39,40.
B-G, Age at death for the indicated endpoint categories in the lifespan study. n=14, 17 (B), 6, 7 (C), 8, 5 (D), 3, 2 (E), 5, 6 (F), 3 (G).

We followed established practices for lifespan study. “Both mice euthanized or found dead were represented as deaths in the survival curves. Mice euthanized due to injuries unrelated to imminent death were treated as censored” (Francesco, Nature 2024). In our study, no mice were removed from the study for reasons unrelated to age-associated decline and censored in the survival analysis. Statistical significance was determined using the log-rank test. We have included this information in the Methods and Table S3 for statistical analyses.

3. Subsection “GLP-1R activation extends healthspan” and Fig. 1. As noted above, healthspan cannot be assessed through endpoint analysis and requires longitudinal data; otherwise, it is impossible to determine when the onset of impairments occurs. In addition, a major general limitation of the experimental design of this study is that baseline measures have not been performed. This precludes one from distinguishing whether the treatment with semaglutide actually delayed the onset of the impairments observed in the control animals and/or whether the animals in the semaglutide group were different from the control ones before initiating the treatment. At most, with this experimental design, the authors can state that “X” variable was improved by the treatment with semaglutide for 3 months (and acknowledging the limitation that they do not have baseline measurements). Therefore, both the title of the subsection “GLP-1R activation extends healthspan” and the last sentence of that subsection in which the authors state “Together, these data suggest that semaglutide treatment extends healthspan“ Statement “semaglutide treatment extends healthspan”, should be revised to accurately reflect their results.

Response: We thank the reviewer for raising these important points. We agree that it is important to perform longitudinal measurements rather than endpoint analyses alone. It is also critical to include baseline measurements. To address this, we performed a longitudinal study in 20-month-old female C57BL/6 mice that were treated with saline, semaglutide, or matched (24%) calorie restriction for 5 months, and assessed at baseline and after 2 and 4 months of intervention. No differences were observed among groups at baseline (Data below)(Extended Data Figure 7).

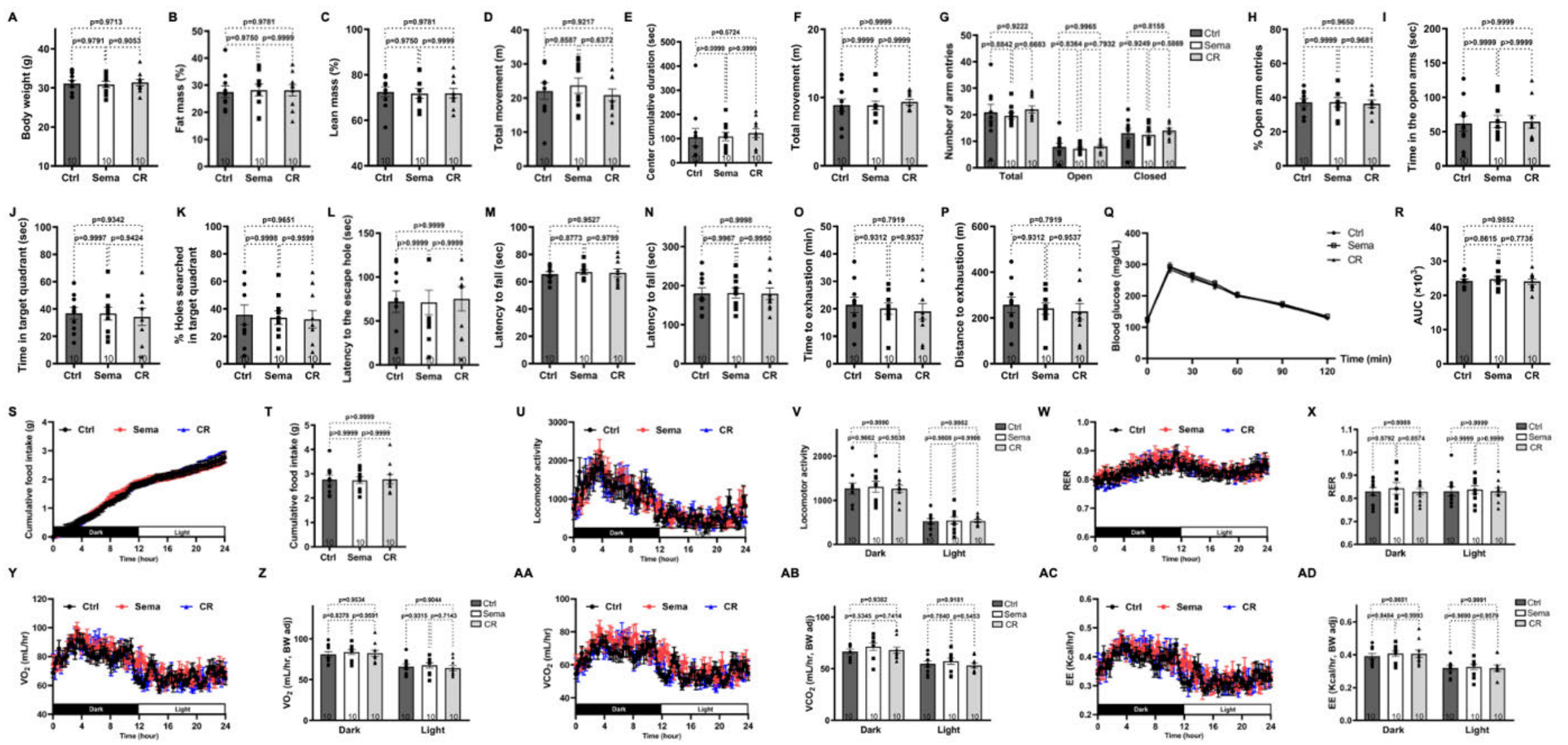

A longitudinal study of 20-month-old female C57BL/6 mice treated with vehicle, semaglutide, or calorie restriction for 5 months. Data shown are baseline physiological measures. n=10.
A, Body weight.
B, C, The percentage of fat mass (B) and lean mass (C) in the Echo MRI test.
D, E, Total movement (D) and center cumulative duration (E) in the open field test.
F-I, Total movement (F), number of arm entries (G), the percentage of open arm entries (H), time in the open arms (I) in the elevated plus maze test.
J-L, Time in target quadrant (J), percentage of holes searched in target quadrant (K), latency to the escape hole (L) in the Barnes maze test.
M, Latency to fall in the rotarod test.
N, Latency to fall in the inverted screen test.
O, P, Time (O) and distance to exhaustion (P) in the treadmill exhaustion test.
Q, R, Blood glucose (Q) and the area under the curve (AUC) (R) in the glucose tolerance test.
S-AD, Measurements in metabolic cage.
S, T, Cumulative food intake (S) and summarized (T).
U, V, Locomotor activity (U) and summarized by light and dark cycles (V).
W, X, Respiratory exchange ratio (W) and summarized by light and dark cycles (X).
Y, Z, Oxygen consumption absolute value (Y) and summarized by light and dark cycles after adjustment using ANCOVA with body weight as a covariate (Z).
AA, AB, Carbon dioxide production absolute value (AA) and summarized by light and dark cycles after adjustment using ANCOVA with body weight as a covariate (AB).
AC, AD, Energy expenditure absolute value (AC) and summarized by light and dark cycles after adjustment using ANCOVA with body weight as a covariate (AD).

Control mice exhibited progressive declines in locomotor activity and exploratory behavior in novel environments, motor coordination, muscle function, glucose control, and spatial memory (Data below A-I)(Figure 5). Semaglutide treated mice showed significantly different longitudinal trajectories from controls across measures, with some outcomes maintained at baseline levels and others significantly improved above baseline (Data below A-I)(Figure 5). These findings indicate that late life GLP-1R activation attenuates age-associated physiological decline.

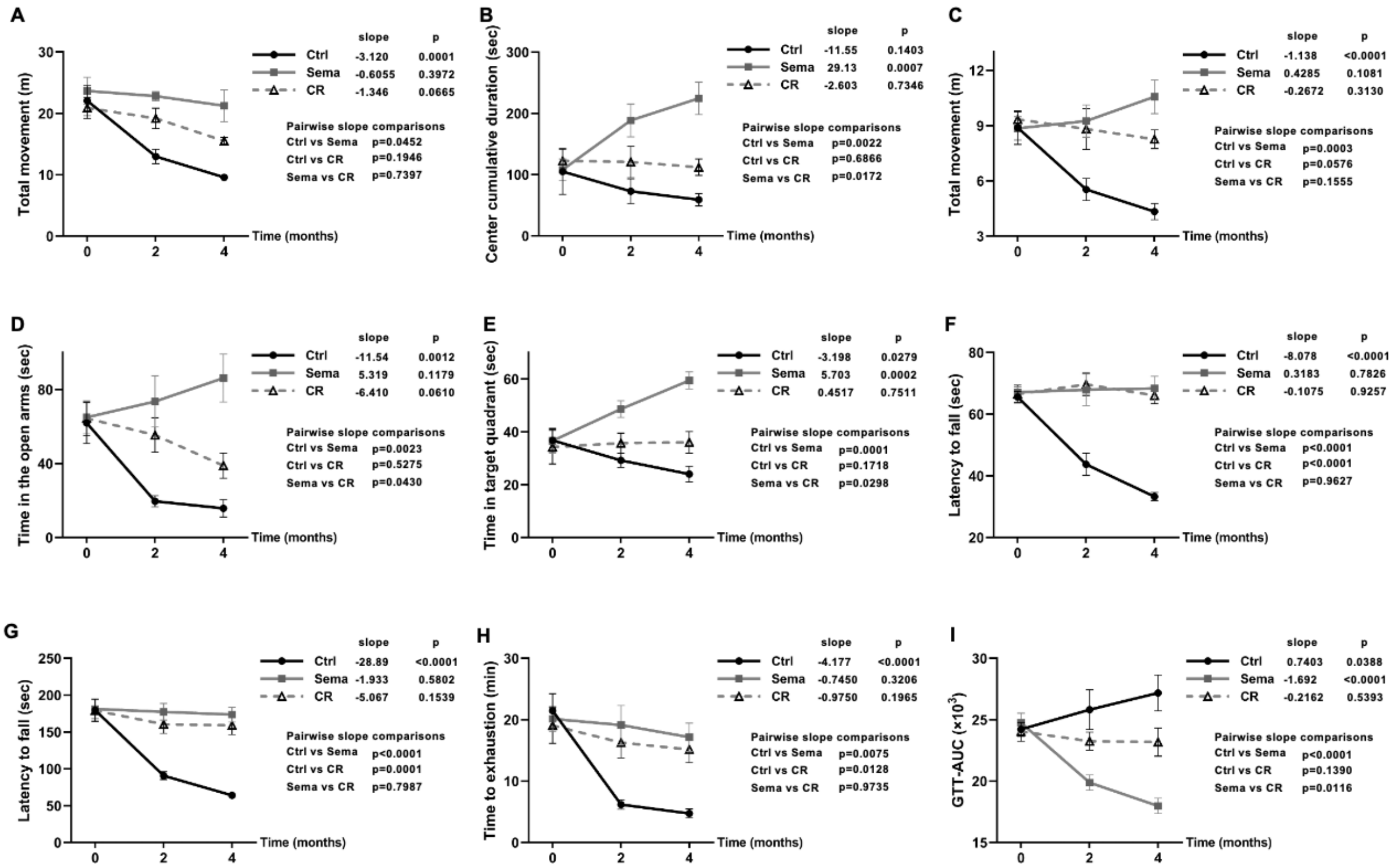

Comparison of 20-month-old female C57BL/6 mice treated with vehicle, semaglutide, or calorie restriction for 5 months. Mice were assessed at the baseline or after 2 or 4 months of treatment. n=10.
A, B, Total movement (A) and center cumulative duration (B) in the open field test.
C, D, Total movement (C) and time in the open arms (D) in the elevated plus maze test.
E, Time in target quadrant in the Barnes maze test.
F, Latency to fall in the rotarod test.
G, Latency to fall in the inverted screen test.
H, Time to exhaustion in the treadmill exhaustion test.
I, The glucose tolerance test.

4. Regarding the results of the behavioral test presented in Fig. 1. Could the increases in locomotor and exploratory behaviors in the semaglutide-treated mice be related with foraging behavior?

Response: We appreciate the reviewer’s thoughtful question. If the increased locomotor/exploratory behaviors reflected foraging driven by hunger, we would expect it to be most evident in calorie restricted mice and to track with food anticipation. Consistent with this, calorie restricted mice typically consumed their daily food allotment soon after it was provided and then underwent a prolonged fasting period (Data below A)(Extended Data Figure 8s) and showed increased activity later in the light cycle when anticipating the next feeding (Data below B)(Extended Data Figure 8u), which is consistent with foraging behavior. In contrast, semaglutide-treated mice had suppressed appetite and consumed their daily intake more gradually across the day (Data below A)(Extended Data Figure 8s), and they did not show the same end-of-day activity increase associated with food anticipation (Data below B)(Extended Data Figure 8u). Thus, the activity pattern in semaglutide-treated mice is not consistent with a hunger-driven foraging explanation.

There is a distinction between general home cage/metabolic cage activity and behavior in novelty-based assays. While overall locomotor activity in the metabolic cage was comparable between groups (Data below C)(Extended Data Figure 8v), both calorie restricted and semaglutide-treated mice showed increased activity in the open field and elevated plus maze (10–15 min tests) (Data below D, E)(Extended Data Figure 8d, f), which primarily assess responsiveness to a novel environment and exploration-related behavior rather than feeding anticipation. We have added this explanation to the manuscript.

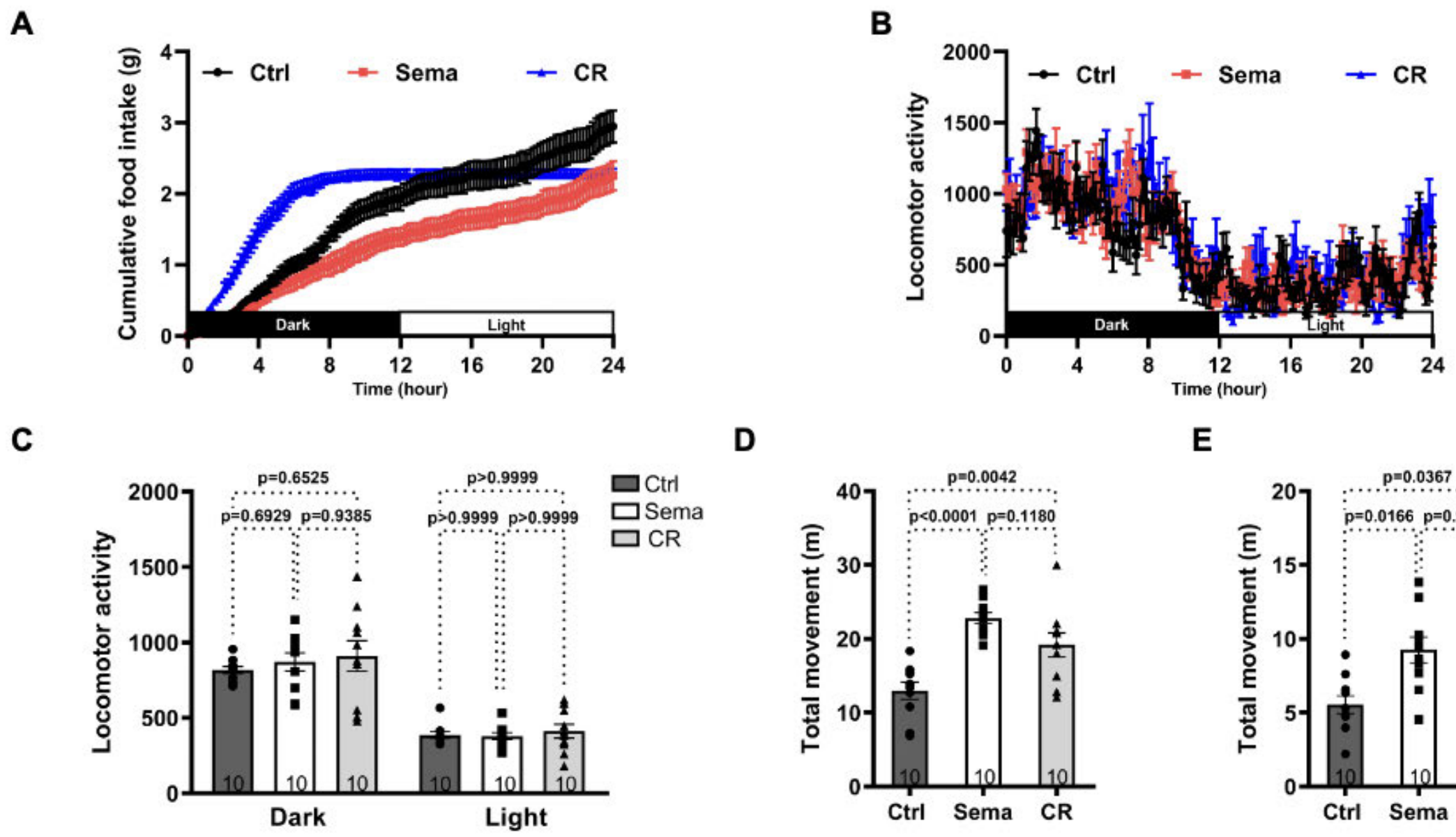

A longitudinal study of 20-month-old female C57BL/6 mice treated with vehicle, semaglutide, or calorie restriction for 5 months. Data shown are physiological measures after 2 months of treatment.
A-C, Measurements in metabolic cage. n=10.
A, Cumulative food intake.
B, C, Locomotor activity (B) and summarized by light and dark cycles (C).
D, Total movement in the open field test. n=10.
E, Total movement in the elevated plus maze test. n=10.

5. Fig. 1: Were the same 10 animals measured in the tests shown in Fig. 1b-q? Was this a separate group of animals or were they a subgroup of the animals used in the lifespan analysis? How were the animals selected if they were a subgroup of the animals used in the lifespan analysis? What was the total number of animals used in this study, including all tests performed and sample collection? Please describe this information in detail in methods or/and results section.

Response: The same 10 mice were used for all tests in Figure 1b-q. This is a separate group of mice from those in the lifespan study. For the lifespan study, 39 mice received saline and 40 mice received semaglutide for the duration of the lifespan. For physiological, molecular, and cellular studies, a separate cohort was treated for 3 months. 10 pairs for physiological assessments, 5 pairs for neural stem cell studies requiring tissue fixation, and 6 pairs for other molecular and cellular analyses. The longitudinal study included a separate cohort of mice (10 mice per group) treated with

saline, semaglutide, or calorie restriction for 5 months. We have included this information in the Methods and the Results.

6. Fig. S1a: The authors show that after ~10 days on treatment, a reduction in body weight is observed in the semaglutide-treated animals compared to the control group. Was food consumption inhibited or reduced by the treatment with semaglutide? Please provide the food consumption trajectories in each experimental treatment, as this information is critical to understand the extent to which the reduction in body weight is explained by reduced feed intake or other mechanisms and, thus, assess the feasibility of the authors' hypothesis regarding that semaglutide can act as a calorie restriction mimetic. Ideally a pair-fed group or weight matched group should be involved to ascertain whether the effect is a drug effect or "simply" a caloric restriction effect.

Response: We thank the reviewer for raising these important points. Food intake data is including below and in Extended Data Figure 1a, b, showing semaglutide reduced food intake by 24%.

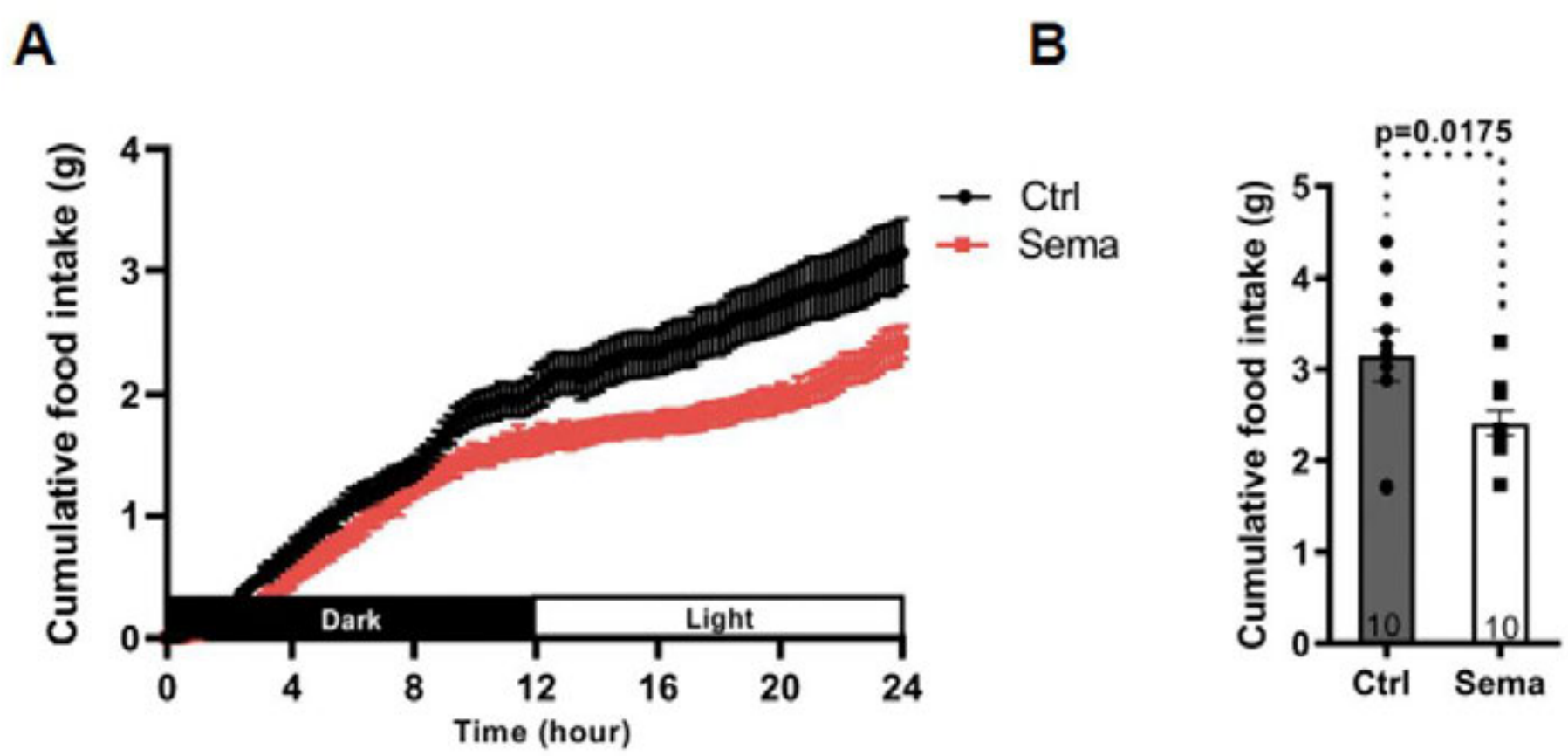

Comparison of 20-month-old female C57BL/6 mice treated with vehicle or semaglutide for 3 months in metabolic cage. Cumulative food intake (A) and summarized (B). n=10.

We agree that it is important to include a pair-fed group. To address this, we performed a longitudinal study in 20-month-old female C57BL/6 mice that were treated with saline, semaglutide, or matched (24%) calorie restriction for 5 months, and assessed at baseline and after 2 and 4 months of intervention. No differences were observed among groups at baseline (Data below)(Extended Data Figure 7).

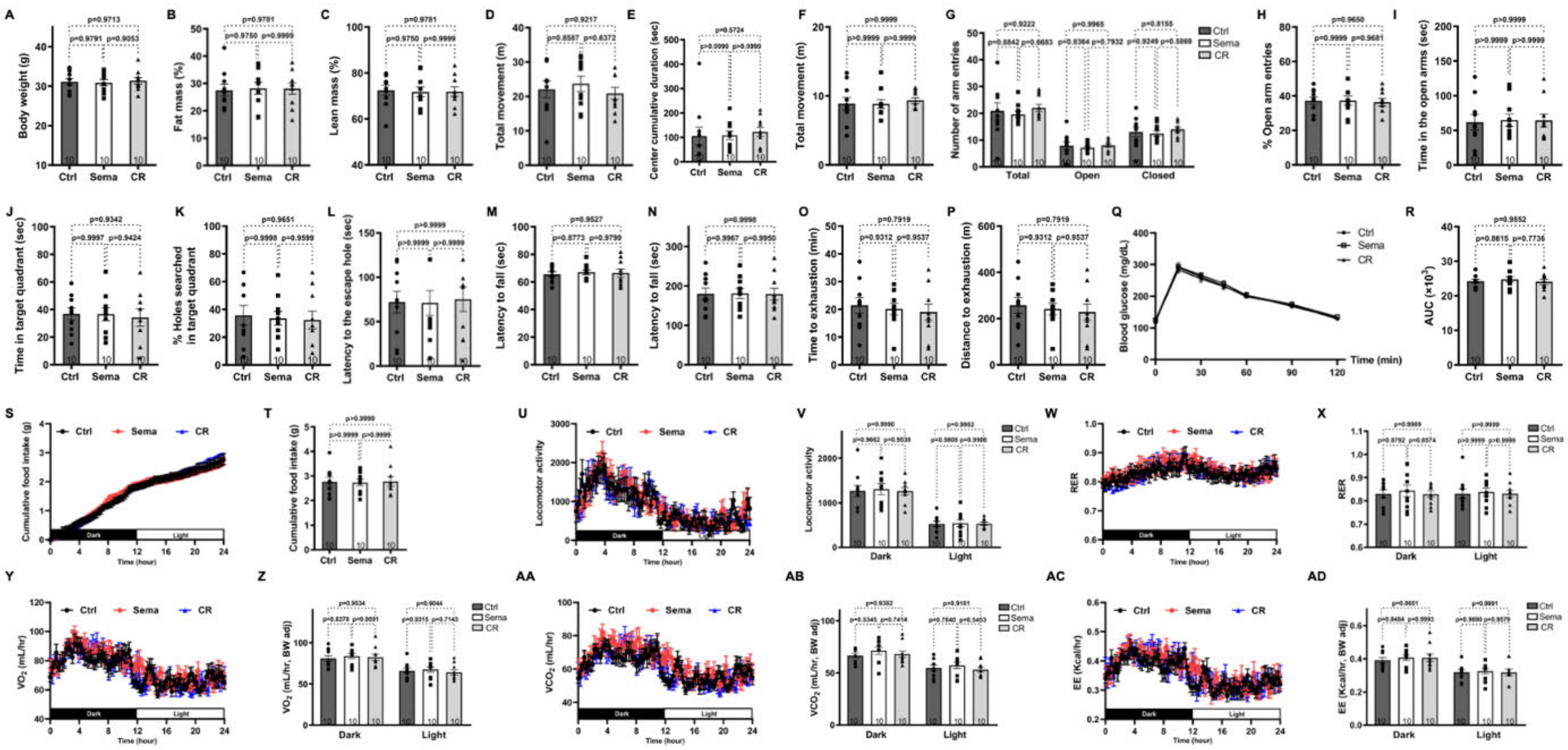

A longitudinal study of 20-month-old female C57BL/6 mice treated with vehicle, semaglutide, or calorie restriction for 5 months. Data shown are baseline physiological measures. n=10.
A, Body weight.
B, C, The percentage of fat mass (B) and lean mass (C) in the Echo MRI test.
D, E, Total movement (D) and center cumulative duration (E) in the open field test.
F-I, Total movement (F), number of arm entries (G), the percentage of open arm entries (H), time in the open arms (I) in the elevated plus maze test.
J-L, Time in target quadrant (J), percentage of holes searched in target quadrant (K), latency to the escape hole (L) in the Barnes maze test.
M, Latency to fall in the rotarod test.
N, Latency to fall in the inverted screen test.
O, P, Time (O) and distance to exhaustion (P) in the treadmill exhaustion test.
Q, R, Blood glucose (Q) and the area under the curve (AUC) (R) in the glucose tolerance test.
S-AD, Measurements in metabolic cage.
S, T, Cumulative food intake (S) and summarized (T).
U, V, Locomotor activity (U) and summarized by light and dark cycles (V).
W, X, Respiratory exchange ratio (W) and summarized by light and dark cycles (X).
Y, Z, Oxygen consumption absolute value (Y) and summarized by light and dark cycles after adjustment using ANCOVA with body weight as a covariate (Z).
AA, AB, Carbon dioxide production absolute value (AA) and summarized by light and dark cycles after adjustment using ANCOVA with body weight as a covariate (AB).
AC, AD, Energy expenditure absolute value (AC) and summarized by light and dark cycles after adjustment using ANCOVA with body weight as a covariate (AD).

Both semaglutide treatment and calorie restriction led to comparable body weight and fat loss (Data below A-C)(Extended Data Figure 10a-c).

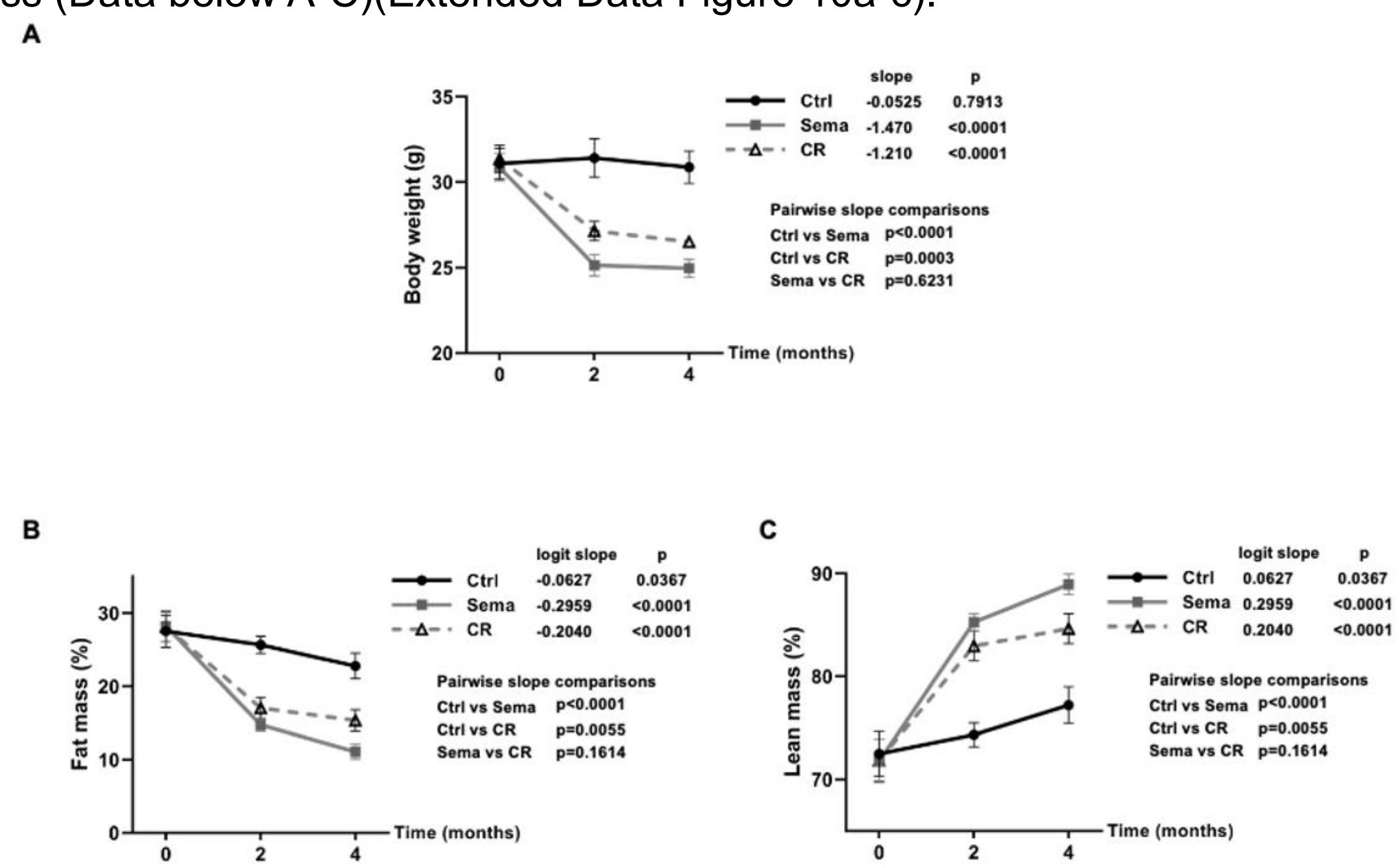

Comparison of 20-month-old female C57BL/6 mice treated with vehicle, semaglutide, or calorie restriction for 5 months. Mice were assessed at the baseline or after 2 or 4 months of treatment. n=10.
A, Body weight.
B, C, The percentage of fat mass (B) and lean mass (C) in the Echo MRI test.

Control mice exhibited progressive declines in locomotor activity and exploratory behavior in novel environments, motor coordination, muscle function, glucose control, and spatial memory over the course of the longitudinal study (Data below A-I)(Figure 5). Semaglutide treated mice showed significantly different trajectories from control mice across measures (Data below A-I)(Figure 5). Semaglutide treatment preserved baseline function and produced trajectories comparable to those of calorie restricted mice for total locomotor activity in the open field (Data below A)(Figure 5a) and elevated plus maze tests (Data below C)(Figure 5c), rotarod performance (Data below F)(Figure 5f), inverted screen performance (Data below G)(Figure 5g), and treadmill endurance (Data below H)(Figure 5h). Notably, semaglutide treatment led to improvements above baseline, and to more favorable trajectories than calorie restriction, in open field center time (Data below B)(Figure 5b), Barnes maze performance (Data below E)(Figure 5e), and glucose tolerance (Data below I)(Figure 5i). These findings indicate that GLP-1R activation late in life recapitulates many functional benefits of calorie restriction by attenuating age-associated decline, while conferring additional benefits in exploratory drive, spatial memory, and glucose control, suggesting effects beyond reduced calorie intake alone.

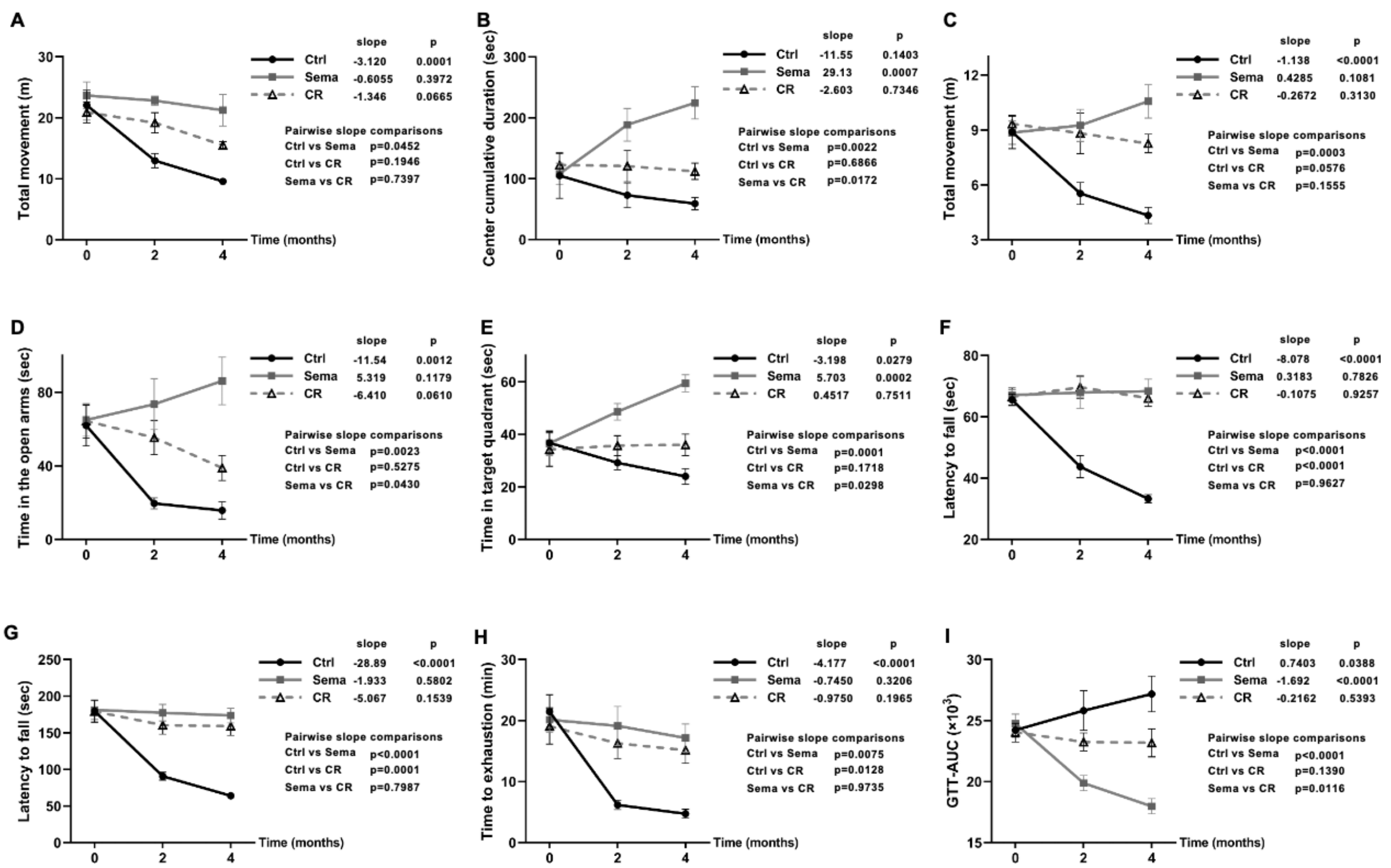

Comparison of 20-month-old female C57BL/6 mice treated with vehicle, semaglutide, or calorie restriction for 5 months. Mice were assessed at the baseline or after 2 or 4 months of treatment. n=10.
A, B, Total movement (A) and center cumulative duration (B) in the open field test.
C, D, Total movement (C) and time in the open arms (D) in the elevated plus maze test.
E, Time in target quadrant in the Barnes maze test.
F, Latency to fall in the rotarod test.
G, Latency to fall in the inverted screen test.
H, Time to exhaustion in the treadmill exhaustion test.
I, The glucose tolerance test.

7. Fig. S1a: Could the authors provide the body weight trajectories of each experimental group before starting the treatment? Please indicate if the trajectories of body weight

correspond to all animals included in the lifespan analysis or if it is a subgroup of animals. It is also important to note that for the age-range shown in this figure (600-780 days of age), it would be expected that control animals start to show a modest decline in body weight (https://doi.org/10.1016/j.cmet.2021.08.013). Discussing these results in light of the available literature for C57BL/6 female mice is strongly recommended. Also, why do the authors choose to show the body weight trajectories in that age-range instead of the entire age-range covered in the lifespan analysis (600-860 days of age)? Why isn't there a statistical analysis associated with these body weight trajectories?

Response: We thank the reviewer for this helpful suggestion. We have revised Fig. S1a (Data below)(New Extended Data Figure 2a) and the corresponding legend/text as follows.

1. We have included body weight trajectories prior to treatment initiation in New Extended Data Figure 2a (Data below). Baseline body weight did not differ between groups (p= 0.147).
2. The body weight trajectories in Fig. S1a (New Extended Data Figure 2a) reflect all animals included in the lifespan cohort. This is now explicitly stated in the figure legend.
3. We agree that a modest late-life decline in body weight has been reported in aging C57BL/6 mice (Palliyaguru, Cell Metab 2021). However, other studies have documented stable body weight beyond 20 months (Fischer, Aging 2016; Ogiso, Exp Anim 2024; Johnson, Aging Pathobiol Ther 2023; Ge, Pathobiology of Aging & Age-related Diseases 2016; Pajski, J Gerontol A Biol Sci Med Sci 2024), consistent with our observations. We have included this discussion in the revised manuscript.
4. We are not including body weight data beyond 810 days because very few (4) control mice remained, making group-level summaries increasingly unstable and formal inference uninformative.
5. We have now added statistical analysis for the body weight data in the Figure and Table S3 for statistical analyses.

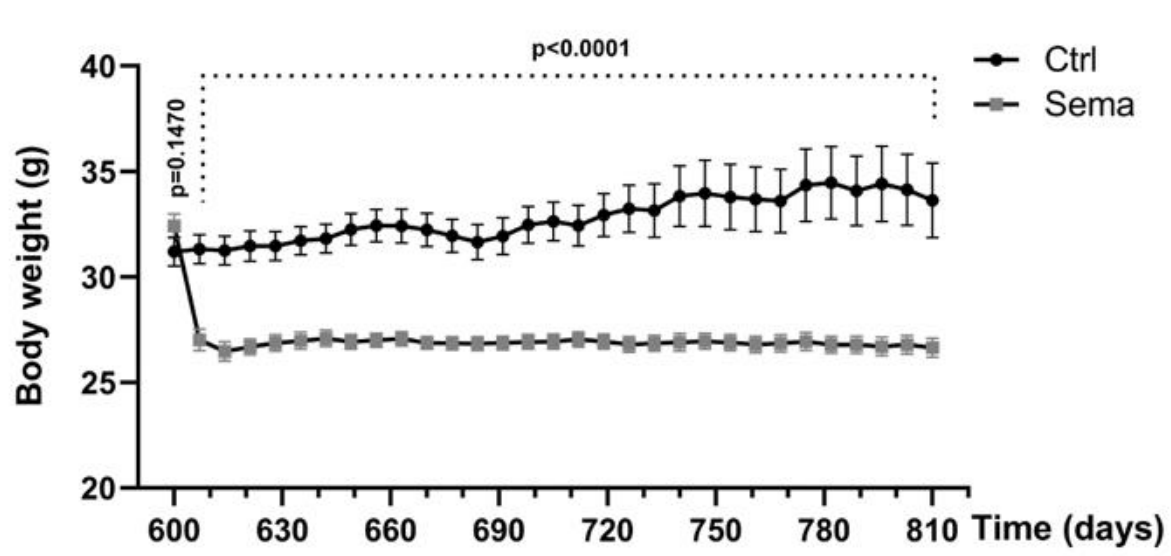

20-month-old female C57BL/6 mice were treated with vehicle or semaglutide for the duration of the lifespan. Body weight of mice in the lifespan study. n=39,40.

8. Fig. 1l,m: In parallel with the analyses of the absolute values of the rotarod and grip strength (sic) tests currently shown, please provide these data analyzed using ANCOVA including body weight as a covariate in the estimation of performance on these tests. This will inform whether the physical performance improvement in the semaglutide group is independent or mediated by body weight/lean mass.

Response: To determine whether improvements in rotarod and inverted screen performance were independent of body weight, we performed ANCOVA with body weight included as a covariate. After adjustment for body weight, the semaglutide group retained a significant increase in latency to fall compared with controls (Data below A, B)(Extended Data Figure 3h, i).

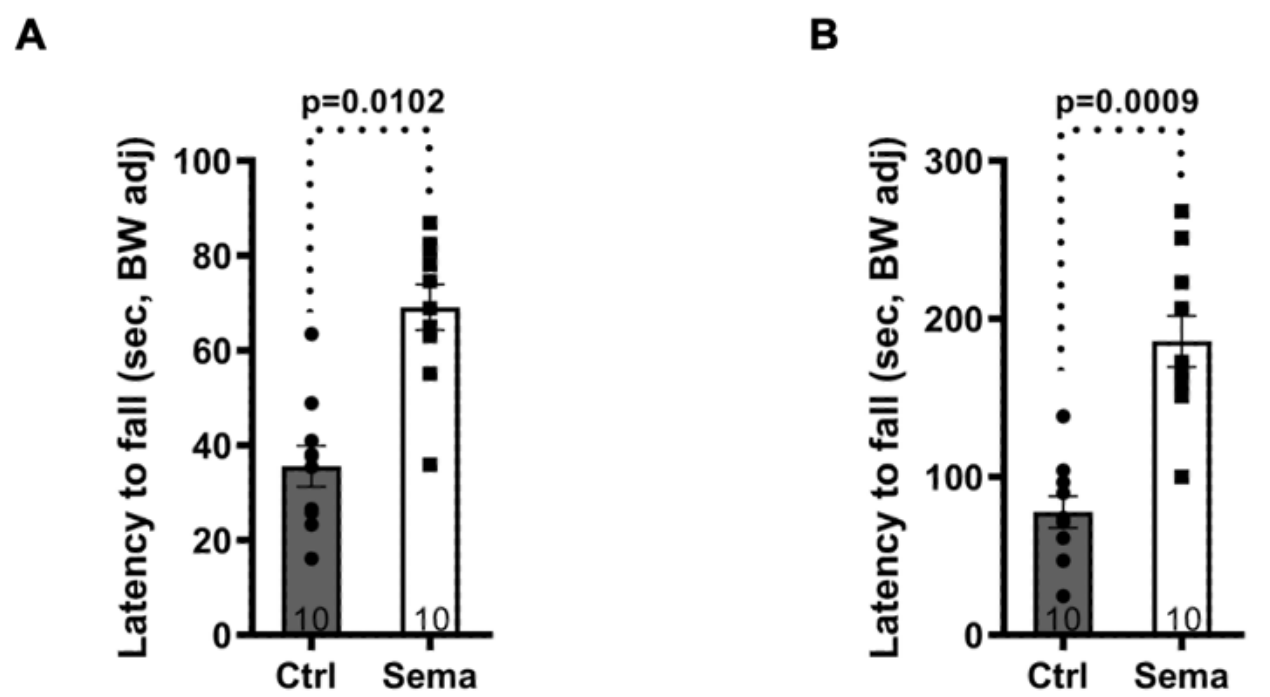

20-month-old female C57BL/6 mice were treated with vehicle or semaglutide for 3 months. n=10.
A, Latency to fall in the rotarod test after adjustment using ANCOVA with body weight as a covariate.
B, Latency to fall in the inverted screen test after adjustment using ANCOVA with body weight as a covariate.

9. Fig. S2: Based on the VO2 and energy expenditure values provided, mice treated with semaglutide would have increased metabolism. However, since the values are presented as ratios to body weight (Kcal/h/kg or ml/h/kg), the leaner semaglutide-treated animals may show apparently higher EE and VO2 only because of how this ratio is calculated and not because they are actually consuming more oxygen. Also, from a statistical standpoint, using the ratio Energy Expenditure/Body Weight can introduce bias if the relation between EE and BW includes an intercept different than zero (i.e., if it is not purely linear). Thus, it is recommended to present the absolute values of these outcomes (Kcal/h or ml/h) in parallel with data adjusted by lean mass (physiologically more relevant than body weight) or body weight as covariates through an appropriate statistical method such as ANCOVA (see for example DOI https://doi.org/10.1038/s41586-024-08026-3). Such method allows to accurately compare experimental groups with different body weight or lean mass without introducing the bias of the method that uses the ratios. On the other hand, in addition to the trajectories of food consumption, please provide the carbon dioxide production levels, the respiratory exchange ratio and locomotor activity obtained with the CLAMS system, since these are fundamental measures to interpret the energy balance and type of substrate being used (fat vs carbohydrate). In addition, the findings emerging from these variables should be discussed more in depth, as they are critical for the authors' hypothesis regarding that semaglutide acts as a calorie restriction mimetic. How do these results align to those observed by indirect calorimetry under calorie restriction? (see for example: https://doi.org/10.1038/s41586-024-08026-3; https://doi.org/10.1016/j.cmet.2023.05.003).

Response: We thank the reviewer for this important point. As suggested by the reviewer, we now present absolute values for oxygen consumption, carbon dioxide production, and energy expenditure, and have additionally analyzed these variables using ANCOVA with body weight as a covariate, as described (Francesco, Nature 2024). We have also included data for food intake, respiratory exchange ratio, and locomotor activity. Semaglutide treatment reduced food intake by 24%, but there were no significant differences in adjusted oxygen consumption, adjusted carbon dioxide production, adjusted energy expenditure, respiratory exchange ratio, and locomotor activity between groups (Data below) (Extended Data Figure 1).

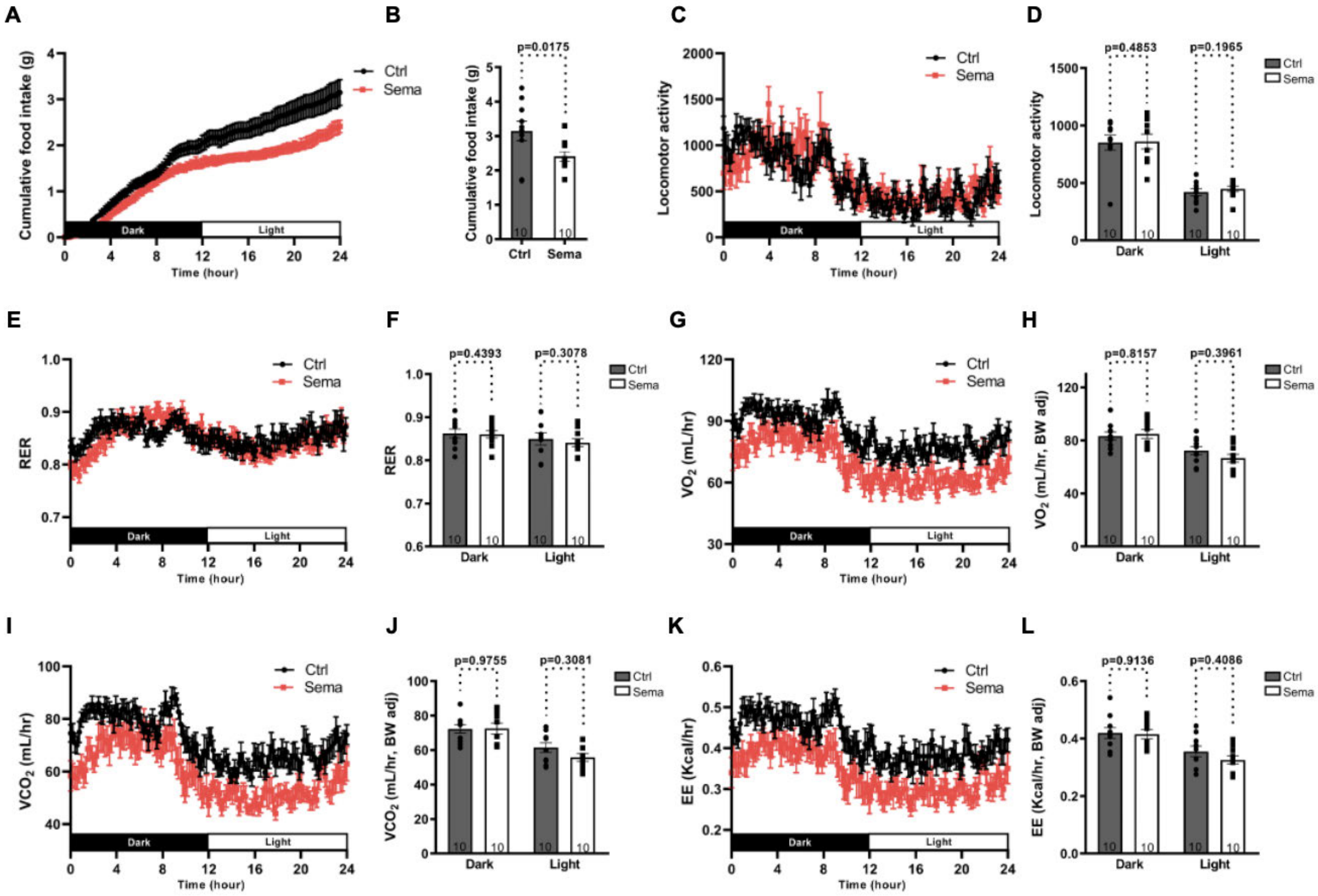

Comparison of 20-month-old female C57BL/6 mice treated with vehicle or semaglutide for 3 months in metabolic cage. n=10.
A, B, Cumulative food intake (A) and summarized (B).
C, D, Locomotor activity (C) and summarized by light and dark cycles (D).
E, F, Respiratory exchange ratio (E) and summarized by light and dark cycles (F).
G, H, Oxygen consumption absolute value (G) and summarized by light and dark cycles after adjustment using ANCOVA with body weight as a covariate (H).
I, J, Carbon dioxide production absolute value (I) and summarized by light and dark cycles after adjustment using ANCOVA with body weight as a covariate (J).
K, L, Energy expenditure absolute value (K) and summarized by light and dark cycles after adjustment using ANCOVA with body weight as a covariate (L).

To address the reviewer's point regarding comparison with calorie restriction, we also compared metabolic cage profiles of semaglutide treated mice and calorie restricted mice. Although the two groups had a similar daily food intake, their feeding patterns differed markedly (Data below A, B)(Extended Data Figure 8s, t). Calorie restricted mice consumed their daily food allotment soon after feeding and then underwent a prolonged fasting period, whereas semaglutide-treated mice consumed food more gradually throughout the day, consistent with appetite suppression. Correspondingly, calorie

restricted mice showed an increase in locomotor activity late in the light cycle when anticipating the next feeding, consistent with foraging behavior, which was not observed in semaglutide treated mice (Data below C)(Extended Data Figure 8u). Calorie restricted mice showed the expected dynamic changes in respiratory exchange ratio, with high values after feeding and lower values during the fasting phase (Data below E, F)(Extended Data Figure 8w, x). In addition, calorie restricted mice showed comparable adjusted oxygen consumption, adjusted carbon dioxide production, adjusted energy expenditure during the dark cycle, but lower adjusted carbon dioxide production and trends toward lower adjusted oxygen consumption and adjusted energy expenditure during the light cycle (Data below G-L)(Extended Data Figure 8y-ad), consistent with the literature (Francesco, Nature 2024). These results indicate that semaglutide and calorie restriction produce a similar reduction in overall calorie intake through distinct mechanisms and physiological states. Calorie restriction is associated with hunger-driven temporal behavioral and metabolic adaptations, whereas semaglutide reduces intake in the context of suppressed appetite and is therefore not accompanied by the same feeding-associated rhythms.

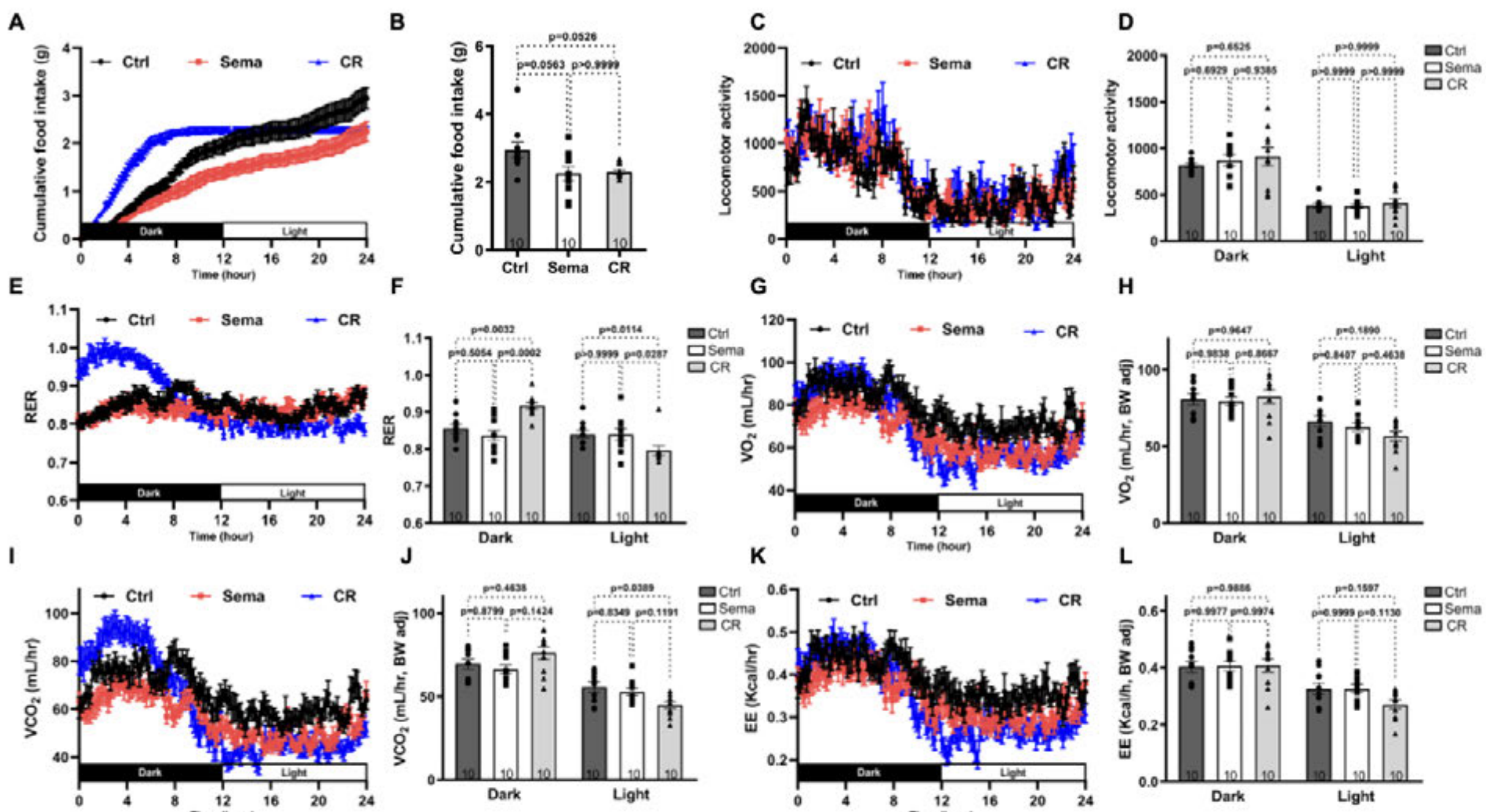

A longitudinal study of 20-month-old female C57BL/6 mice treated with vehicle, semaglutide, or calorie restriction for 5 months. Data shown are measures in metabolic cage after 2 months of treatment. n=10.
A, B, Cumulative food intake (A) and summarized (B).
C, D, Locomotor activity (C) and summarized by light and dark cycles (D).
E, F, Respiratory exchange ratio (E) and summarized by light and dark cycles (F).
G, H, Oxygen consumption absolute value (G) and summarized by light and dark cycles after adjustment using ANCOVA with body weight as a covariate (H).
I, J, Carbon dioxide production absolute value (I) and summarized by light and dark cycles after adjustment using ANCOVA with body weight as a covariate (J).
K, L, Energy expenditure absolute value (K) and summarized by light and dark cycles after adjustment using ANCOVA with body weight as a covariate (L).

10. Several statements and conclusions regarding the mechanisms and effects proposed to be modulated by semaglutide are based on mRNA expression only (e.g., “GLP-1R activation improves mitochondrial function”). Modulation of gene expression levels by semaglutide is an important result indicative of its mechanism of action, but

without functional measures (protein levels, enzymatic activity, among others), it is not possible to make firm conclusions on that regard. For example, in Fig. S7, the increased mRNA levels of Pgc1a, Nrf1, Tfam, Mfn1, Mfn2, ND1, ND4, Cytb, Cytc, Atp6 y Cox1 are indicative of improved mitochondrial functon(in terms of biogenesis, dynamics, respiratory capacity, etc.). However, to firmly state that mitochondrial function is improved it is necessary to confirm these findings with functional measures such as respiratory activity, ATP production, protein levels, etc. The same is valid for Figure S8, where the increased mRNA levels of Nrf2, Cat, Hmox1 and Nqo1 in the semaglutide group is an indicator that the antioxidant response is active and potentially improved, but these transcriptomic measures need to be complemented with protein levels and enzymatic activity (e.g., CAT, HO-1, NQO1, reduction of ROS and markers of oxidative stress).

Response: We thank the reviewer for this important point. We agree that changes in mRNA expression alone are insufficient to make definitive conclusions about mitochondrial functional capacity or oxidative stress response. As suggested by the reviewer, we performed functional assays to complement the mRNA expression results in Fig. S7 and Fig. S8 (New Extended Data Figure 5). To assess mitochondrial function, we quantified ATP levels and found that semaglutide treatment increased ATP content (Data below A)(Extended Data Figure 5g). To assess oxidative stress response, we measured cellular ROS using MitoSOX staining and found that semaglutide treatment reduced ROS levels (Data below B)(Extended Data Figure 5h). Together, these functional readouts support the interpretation that the observed increases in mitochondrial and oxidative stress response transcripts are associated with improved mitochondrial energetic status and reduced oxidative stress in semaglutide-treated mice.

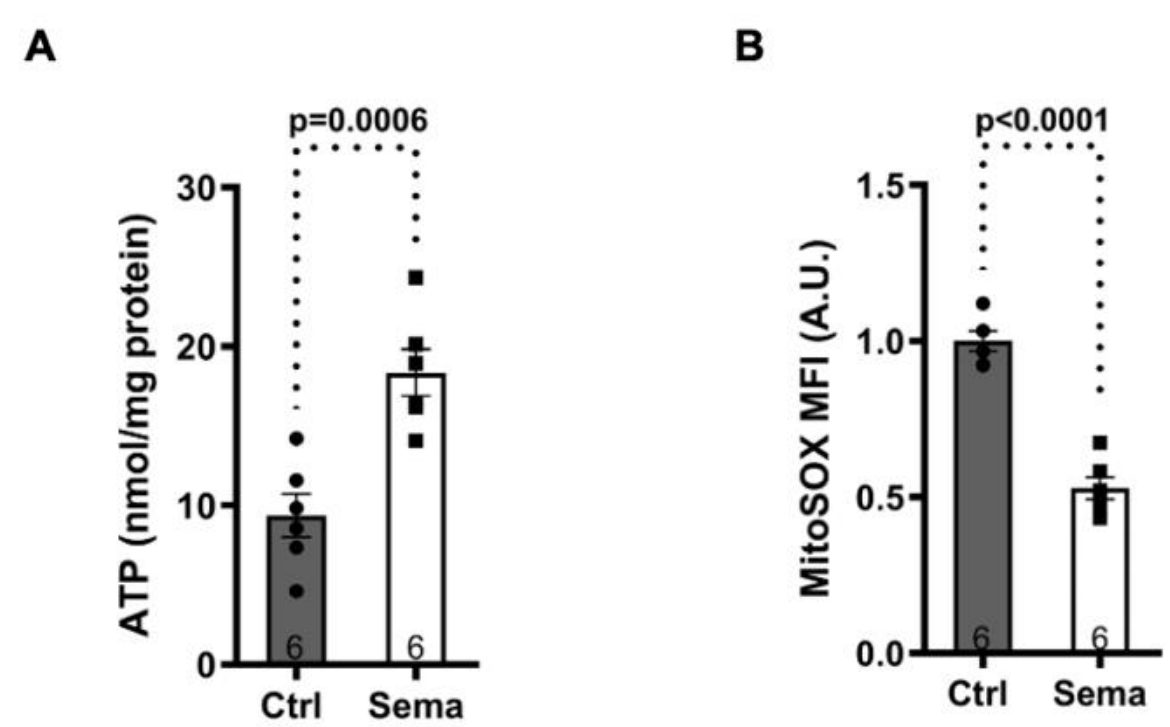

Comparison of 20-month-old female C57BL/6 mice treated with vehicle or semaglutide for 3 months.
A, Quantification of ATP in the muscle. n=6.
B, Quantification of flow cytometry analysis of MitoSOX staining of HSCs. n=6.

11. Minor comment: The color of the treatments in Fig. S5e,g is inverted with regards to the rest of the figures of the manuscript.

Response: We have changed the color for consistency.

Discussion section

1. The findings of this study should be discussed more in depth, both in terms of similarities and discrepancies with previous studies on GLP-1 receptor agonists and regarding the authors' hypothesis that semaglutide acts as a calorie restriction mimetic.

Response: We have expanded the Discussion section to put our study in the context of the existing literature. Preclinical studies in disease mouse models (Petersen, Nature 2024; Ma, Nature Communications 2024; Sourris, Kidney International 2024; Jara, Nature Medicine 2025; Zhang, Nature Aging 2025) and clinical studies (Garvey, Nature Medicine 2022; Weghuber, The New England Journal of Medicine 2022; Lincoff, The New England Journal of Medicine 2023; McGuire, The New England Journal of Medicine 2025; Apperloo, Nature Medicine 2025; Perkovic, The New England Journal of Medicine 2024; Sanyal, The New England Journal of Medicine 2025; Athauda, Lancet 2017; Meissner, The New England Journal of Medicine 2024) have revealed pleiotropic beneficial effects of GLP-1R activation, including improved glucose and weight control, and reduced cardiovascular, renal, hepatic, and neurodegenerative disease burden. We found that GLP-1R activation late in life improved physiological function and extended lifespan in an aging mouse model (20-month-old female C57BL/6) treated with a defined semaglutide regimen that reduced food intake by 24%.

We have also discussed how our data support the hypothesis that semaglutide acts as a calorie restriction mimetic. Our findings establish that GLP-1R activation late in life alleviates broadly aging-associated decline and phenocopies the molecular and physiological benefits of calorie restriction. In aged mice, semaglutide treatment extended lifespan, improved physiological function (Figure 1, Extended Data Figure 2, 3), reduced hallmarks of aging such as stem cell attrition, inflammation, cellular senescence, genomic instability, mitochondrial dysfunction, and loss of proteostasis (Figure 2, 3, Extended Data Figure 4-6), and modulated the genetic regulators of aging and nutrient sensors in the same fashion as calorie restriction (Figure 4). Direct comparison of semaglutide treatment with matched calorie restriction further showed comparable effects across several aspects of aging-associated physiological decline (Figure 5a, c, f, g, h), consistent with the idea that GLP-1 medicines can act as calorie restriction mimetics. Because aging is the biggest risk factor for numerous chronic diseases and calorie restriction slows aging and ameliorates a broad spectrum of aging-associated diseases, our findings raise the possibility that GLP-1 medicines may influence a wide array of seemingly unrelated diseases by slowing aging.

Our study also reveals that GLP-1R activation exerts effects beyond those attributable to reduced calorie intake alone (Figure 5b, e, i). Whereas calorie restriction generally maintained exploratory drive, spatial memory, and glucose control near baseline levels, semaglutide treatment significantly improved these outcomes above baseline, raising the possibility that GLP-1 medicines may not only slow but reverse aspects of aging-associated functional decline.

2. Do the authors recognize any limitations in their study? Are there any adverse effects of semaglutide?

Response: We thank the reviewer for raising this point. Our study was designed to test the effect of late-life GLP-1R activation on aging-associated decline at the molecular, cellular and organismal levels, and lifespan in an established mouse aging model (20-month-old C57BL/6) using a defined semaglutide regimen, enabling controlled assessment. Female mice were selected to minimize confounding effects of male aggression and injury, consistent with prior long-term aging studies (Francesco, Nature 2024). Within the endpoints monitored in this study, we did not observe adverse effects attributable to semaglutide. Importantly, while semaglutide is widely used clinically, whether GLP-1R activation modulates aging trajectories and lifespan in humans will require long-term clinical studies designed to evaluate aging-related outcomes in older populations. We have included this information in Discussion section of the manuscript.

Methods section

1. In the statistical analysis section, the authors indicate: “Mice were randomized to groups and analysis of mice and tissue samples were performed by investigators blinded to the treatment of the animals. Measurements were taken from distinct samples.” Please indicate if treatment groups had homogeneous body weights at randomization, the total number of animals used in the study, including all experiments, and the method to select the animals (if any) for the functional measures performed.

Response: This information is included in the Methods section. For the lifespan study, 39 mice received saline and 40 mice received semaglutide for the duration of the lifespan. For physiological, molecular, and cellular studies, a separate cohort was treated for 3 months. 10 pairs for physiological assessments, 5 pairs for neural stem cell studies requiring tissue fixation, and 6 pairs for other molecular and cellular analyses. For longitudinal study, a separate cohort was treated with saline, semaglutide, or calorie restriction for 5 months (10 mice per group).

There was no body weight difference at randomization in the experiments comparing mice treated with saline and semaglutide for lifespan (Data below A), physiological assessments (Data below B), neural stem cell studies (Data below C), and other molecular and cellular studies (Data below D). There was also no body weight difference at randomization in the longitudinal study comparing mice treated with saline, semaglutide, and calorie restriction (Data below E).

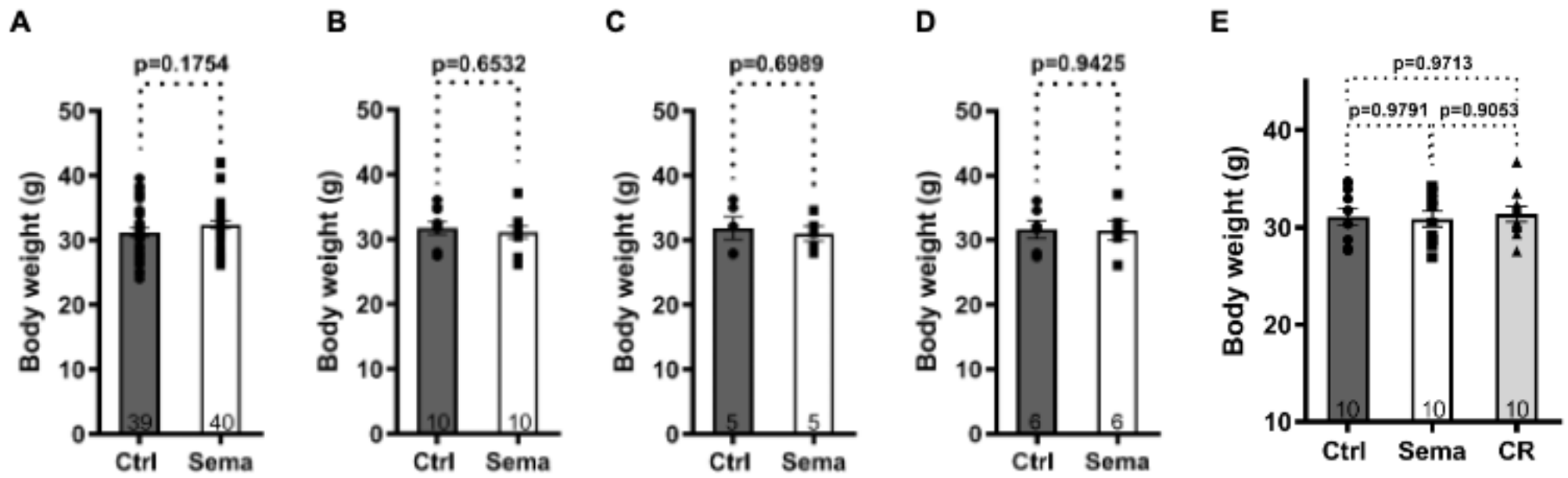

A, 20-month-old female C57BL/6 mice were treated with vehicle or semaglutide for the duration of the lifespan for lifespan study. Data shown are baseline body weight. n=39,40.
B-D, 20-month-old female C57BL/6 mice were treated with vehicle or semaglutide for 3 months for physiological assessments (B, n=10), neural stem cell studies (C, n=5), and other molecular and cellular analyses (D, n=6). Data shown are baseline body weight.
E, A longitudinal study of 20-month-old female C57BL/6 mice treated with vehicle, semaglutide, or calorie restriction for 5 months. Data shown are baseline body weight. n=10.

2. Please indicate in the methods section for how long the mice were acclimated to the same conditions after arrival at the facility and before starting the experimental treatments.

Response: We have included this information in the Methods section. “Mice were acclimated for a week after arrival at the facility and before starting the experimental treatments or baseline measurements”.

3. Please indicate in methods section how mice were housed (group-housed? single-housed?) as this factor greatly affects behavior towards food consumption and thermoregulation, which impact the overall energetic context, a key aspect for this study.

Response: We have provided this information in the Methods section. “As described (Francesco, Nature 2024), group housing is standard practice for calorie restriction studies. Mice were group-housed on a 12:12 h light:dark cycle at 25 °C. Control and semaglutide-treated groups had ad libitum access to food and water. Calorie restricted mice were provided with unlimited access to water and measured amounts of food daily. Competition for food was minimized by placing food directly into the bottom of the cage, allowing individual mice to get a pellet.”

4. Please provide all details describing how subcutaneous injections were performed (and corresponding reference of the protocol), as their short- and long-term effect on local inflammation, tissue damage, and ulceration may not be trivial for several of the biomarkers measured in this study as well as from an ethical standpoint. Did the operator rotate the injection sites throughout the study?

Response: We thank the reviewer for raising this important point. We have included this information in the Methods section. “For subcutaneous injection, we followed the standard operating protocol established by the animal care committee at the University of California, Berkeley. Briefly, subcutaneous injections were performed daily using a 28-gauge needle in the dorsal subcutaneous region. To reduce discomfort, a new

needle was used for each animal. To minimize local tissue irritation and the risk of chronic inflammation, the operator systematically rotated injection sites across the dorsal surface, ensuring no single site was used repeatedly in consecutive days. Animals were monitored daily for local reactions, including erythema, swelling, ulceration, or tissue damage. No significant adverse effects were observed at any time point".

5. What the authors call "Grip Strength Test" in literature is commonly referred to as "cage top test", "inverted screen test", "grid hanging test", "hanging test", "cage wire test" (see: DOI: 10.1126/science.aat3987; 10.1038/bonekey.2015.101; https://doi.org/10.1038/s41596-019-0256-1), among other synonyms. It is strongly recommended to refer to this test with one of these nomenclatures, since the "grip strength test" measures the force (itself) of the limbs obtained with a grip strength meter and, thus, it is not a latency to fall.

Response: We thank the reviewer for pointing this out. We have changed to inverted screen test.

6. Most (if not all) statistical analyses in the manuscript were performed through t-tests, which require that the assumptions of normality and homoscedasticity are satisfied. Did the authors test these assumptions for each variable? How? If so, this has not been described in the statistical analysis section and should be provided, indicating by which method this was achieved. Visual inspection (e.g., Fig 1H, Fig. 4e-Sirt5, to name just two) and the nature of some variables (counts, percentages) suggest that the assumptions of this test could have been violated, and for such cases it is strongly recommended to perform estimations using models that are appropriate for the natural distribution of the variables (e.g., Poisson distribution for variables that are counts), tests that allow heterogeneous variances between groups (e.g., Welch's t-test?) or non-parametric tests (e.g., Mann–Whitney U, Wilcoxon signed-rank) as needed. In addition, please provide the exact p-values as well as the coefficients of the statistics used for each test.

Response: We thank the reviewer for the important comment. We have reassessed all analyses. Data were tested for normality using the Shapiro-Wilk test. Normally distributed data were tested for homoscedasticity using F-test or Brown-Forsythe test. For two-group continuous data, non-normally distributed data were analyzed using the Mann-Whitney U test. Student's t-test was used for data with equal variances and Welch's t-test was used for data with unequal variances. For three-group continuous data, Kruskal-Wallis test was used for non-normally distributed data, followed by Dunn's multiple comparisons test, one-way ANOVA for normally distributed data with equal variances, followed by Tukey's multiple comparisons test, and Welch's ANOVA for normally distributed data with unequal variances, followed by Games-Howell's multiple comparisons test. Covariate-adjusted analyses were performed using ANCOVA. Count data were analyzed using generalized linear models with Poisson or negative binomial distribution when there was overdispersion. Percentage data were analyzed using beta

regression. GTT data were analyzed using two-way repeated-measures ANOVA followed by Sidak’s multiple comparisons between groups at each time point. Lifespan data were analyzed using the log-rank test. Categorical distribution data were analyzed using the Fisher-Freeman-Halton exact test. Weekly body weight data were analyzed using linear mixed-effects models, followed by Holm-adjusted multiple comparisons between groups at each time point. Other longitudinal continuous data were analyzed using linear mixed-effects models to assess aging trajectories. Slopes for each group were determined and pairwise comparisons of slopes were performed with Tukey adjustment. Longitudinal percentage data were analyzed using beta mixed-effects models to assess aging trajectories, with pairwise comparisons of slopes performed using Tukey adjustment. The exact p-values and the coefficients are provided in Figures and Table S3.

Referee #3 (Remarks to the Author):

I co-reviewed this manuscript with one of the reviewers who provided the listed reports.
